# Mining of Cloned Disease Resistance Gene Homologs (CDRHs) in *Brassica* Species and *Arabidopsis thaliana*

**DOI:** 10.3390/biology11060821

**Published:** 2022-05-26

**Authors:** Aldrin Y. Cantila, Ting X. Neik, Soodeh Tirnaz, William J. W. Thomas, Philipp E. Bayer, David Edwards, Jacqueline Batley

**Affiliations:** 1 School of Biological Sciences, The University of Western Australia, Perth 6009, Australia; aldrin.cantila@research.uwa.edu.au (A.Y.C.); soodeh.tirnaz@uwa.edu.au (S.T.); william.thomas@research.uwa.edu.au (W.J.W.T.); philipp.bayer@uwa.edu.au (P.E.B.); dave.edwards@uwa.edu.au (D.E.); 2 Sunway College Kuala Lumpur, Bandar Sunway 47500, Selangor, Malaysia; tingxiang@gmail.com

**Keywords:** Brassicaceae, cloned genes, disease resistance homologs, oilseed mustard

## Abstract

**Simple Summary:**

Developing cultivars with resistance genes (*R* genes) is an effective strategy to support high yield and quality in *Brassica* crops. The availability of clone *R* gene and genomic sequences in *Brassica* species and *Arabidopsis thaliana* provide the opportunity to compare genomic regions and survey *R* genes across genomic databases. In this paper, we aim to identify genes related to cloned genes through sequence identity, providing a repertoire of species-wide related *R* genes in *Brassica* crops. The comprehensive list of candidate *R* genes can be used as a reference for functional analysis.

**Abstract:**

Various diseases severely affect *Brassica* crops, leading to significant global yield losses and a reduction in crop quality. In this study, we used the complete protein sequences of 49 cloned resistance genes (*R* genes) that confer resistance to fungal and bacterial diseases known to impact species in the Brassicaceae family. Homology searches were carried out across *Brassica napus, B. rapa, B. oleracea, B. nigra, B. juncea, B. carinata* and *Arabidopsis thaliana* genomes. In total, 660 cloned disease *R* gene homologs (CDRHs) were identified across the seven species, including 431 resistance gene analogs (RGAs) (248 nucleotide binding site-leucine rich repeats (NLRs), 150 receptor-like protein kinases (RLKs) and 33 receptor-like proteins (RLPs)) and 229 non-RGAs. Based on the position and distribution of specific homologs in each of the species, we observed a total of 87 CDRH clusters composed of 36 NLR, 16 RLK and 3 RLP homogeneous clusters and 32 heterogeneous clusters. The CDRHs detected consistently across the seven species are candidates that can be investigated for broad-spectrum resistance, potentially providing resistance to multiple pathogens. The *R* genes identified in this study provide a novel resource for the future functional analysis and gene cloning of Brassicaceae *R* genes towards crop improvement.

## 1. Introduction

The Brassicaceae family consists of 44 tribes, 372 genera and 4060 species [1,2]. Among these, there are two prominent genera: *Arabidopsis*, which contains the model organism *A. thaliana*, and *Brassica*, which contains species such as *B. napus, B. oleracea*, *B. nigra*, *B. rapa*, *B. carinata*, and *B. juncea,* which are cultivated as a source of vegetables, condiments and oil [3,4,5,6].

The Brassicaceae ancestral genome has undergone three rounds of whole genome duplication/triplication, leading to the evolution of *Arabidopsis* and *Brassica* species [7,8,9,10]. A lineage separation occurred between the 2 genera~29.50 million years ago (MYA) [11], followed by the divergence of the *Brassica* A, B, and C sub-genomes. The diploid *B. nigra* (BB, 2n = 16) diverged from *B. rapa* (AA, 2n = 20) and *B. oleracea* (CC, 2n = 18)~6.2 to 7.9 MYA [9,12] and the divergence between the A and C sub-genomes occurred 4.6 MYA later [13]. Interspecific hybridisation, followed by polyploidisation, in *Brassica* species resulted in 3 allotetraploids; *B. juncea* (AABB, 2n = 4x = 36), which evolved~0.039–0.055 MYA [14], *B. carinata* (BBCC, 2n = 4x = 34) which evolved~0.047 MYA [11], and *B. napus* (AACC, 2n = 4x = 38), being the most recent species to evolve some~7500 years ago [15]. The gene content of these allopolyploids represents a history of gene loss and duplication due to polyploidisation and whole genome duplication [16,17].

The large demand and intensified cultivation of *Brassica* crops have made them vulnerable to abiotic and biotic stresses, particularly to diseases. While the most common control methods for managing pathogens are specific cultural practises and chemical application, the deployment of disease-resistant crops is more environmentally friendly and cost-effective. *Brassica* crops have two types of disease resistance: qualitative and quantitative. While quantitative relies on several minor genes with partial resistance expressed at the later crop stages, qualitative resistance is governed by major genes or resistance genes (*R* genes), largely expressed in the early crop stages through to maturity. Both resistance types are useful, however, qualitative is widely utilised in *Brassica* cultivar development because its effect is easily manifested and can be easily identified at the cotyledon stage. For instance, a set of differential blackleg isolates containing avirulence (*Avr*) genes is used to screen *R* genes in *B. napus* lines via the assessment of a hypersensitive response (HR) observed in the cotyledons [18,19].

Resistance gene analogs (RGAs) play an important role in host resistance [20] and are generally categorized into three main classes, nucleotide-binding site -leucine rice repeats (NLRs), receptor-like protein kinases (RLKs), and receptor-like proteins (RLPs). The NLR family, which is the most common class of RGAs, carries cytoplasmic receptors for recognising specific pathogens and are involved in effector-triggered plant immunity (ETI) [21,22,23,24]. RLKs and RLPs are involved in pattern-triggered immunity (PTI), which relies on pattern recognition receptors (PRRs) to elicit the first line of defence by recognising pathogen elicitors [25,26].

Examining gene homology among plant species is important to obtain the possible functions of a gene. Several studies have exploited gene homology for crop improvement. For instance, the homolog of an *A. thaliana R* gene, *At_NDR1*, was cloned and functionally characterised in *Coffea arabica*, conferring *R*-gene-mediated resistance to coffee leaf rust caused by *Hemileia vastatrix* [27]. Further, homologs of the *Triticum aestivum Mla* gene, *TmMla* in *Hordeum vulgare*, *Sr33* in *Secale cereale*, and *Sr50* in *Aegilops tauschii* were introgressed in *T. aestivum*, providing disease resistance [28,29,30].

Here, we used the sequences of 49 cloned *R* genes with a confirmed function against fungal and bacterial diseases to identify cloned disease resistance gene homologs (CDRHs) across six *Brassica* crops and *A. thaliana*. The evolutionary events including the loss, retention, and diversification of RGA domains in the CDRHs were also investigated. The outcome of this study could facilitate the identification and cloning of functional RGAs and their application in *Brassica* breeding programs towards disease resistance improvement.

## 2. Materials and Methods

### 2.1. Collection of Gene and Genomic Data

A comprehensive search was conducted to identify cloned *R* genes that provide qualitative resistance to fungal and bacterial diseases in all six *Brassica* species and *Arabidopsis*. A total of 49 cloned *R* genes were identified and included in this study (42 in *Arabidopsis* and 7 in the *Brassica* species) based on the following 3 criteria: (1) has a known gene-for-gene interaction with a corresponding pathogen *Avr* gene or (2) confers resistance in the form of a HR, indicating that it is involved in a gene-for-gene interaction or (3) acts as a helper or accessory gene necessary for the gene-for-gene interaction (Table 1). The complete protein (amino acid, aa) sequence of each gene was extracted from the UniProtKb (https://www.uniprot.org/uniprot/, accessed on 10 October 2020) [31] or NCBI (https://www.ncbi.nlm.nih.gov/, accessed on 10 October 2020) website (Table 1). The genome used for each of the seven species is listed in Table 2.

### 2.2. Homolog Identification and Classification

To perform the homology search, the protein sequence of each of the cloned genes was aligned across the seven genomes using translated Basic Local Alignment Search Tool (tBLASTn) using CoGeBlast [93]. Following the criteria used by previous studies identifying homologous genes in plants, tBLASTn hits with an E value range outside E0 to E-45 [94,95,96] or which did not have >70% similarity [96,97,98,99] were removed from further analyses. Since the smallest reference gene used in the study, *At_Rpw8.1*, has 148 aa [75], any tBLASTn hits with <148 aa, were also removed from further analyses.

The list of predicted RGAs derived from the RGAugury pipeline [100] in *A. thaliana*, *B. rapa*, *B. nigra*, *B. oleracea, B. juncea* and *B. napus* were extracted from a previous study [101] and used to classify homologs. The RGAugury pipeline was also used to predict *B. carinata* RGAs in this study. The total number of CDRHs and their RGA classification including Nucleotide-binding site (N), Coiled-coil (CC)-NBS-Leucine rich repeats (LRR) (CNL), CC-NBS (CN), NBS-LRR (NL), Toll/Interleukin-1 receptor (TIR)-NBS-LRR (TNL), TIR-NBS (TN), TIR with unknown domains (TX), NLR with other domains (Other-NLR), Receptor-like kinase protein (RLK) with LRR (LRR-RLK), RLK with Lysin motif (LsyM) (LysM-RLK), RLK with other receptor (Other-RLK), Receptor-like protein (RLP) with LRR (LRR-RLP) and RLP with LysM (LysM-RLP) were identified for each species. We further classified the RGAs according to whether they had the same predicted domain to their homologous counterpart, or whether it was different.

Homolog types, such as paralog (homologous genes within the same species) or ortholog (homologous genes in different species), were also determined for each of the 49 cloned *R* genes. Paralogs were further classified as tandem, when a paralog exists within 5 Mb of the cloned *R* gene, or segmented, when a paralog is >5 Mb away from the cloned gene or the paralog is located on another chromosome [102]. Lastly, genes that were homologous to two or more cloned RGAs were also identified.

### 2.3. Gene Cluster Analysis

Two types of gene clusters were identified in this study. The first was a homogenous RGA cluster which is defined as a cluster with at least 2 or more (but no more than 8) RGAs of the same class, either NLR, RLK or RLP, located within a 200 kb region on the same chromosome [103,104]. The second was a heterogeneous cluster which refers to clusters containing different classes of RGAs or containing both an RGA and a Non-RGA (for example, a homolog that has not been identified using the RGAugury pipeline).

## 3. Results

### 3.1. Distribution of CDRHs

**Figure 1:**
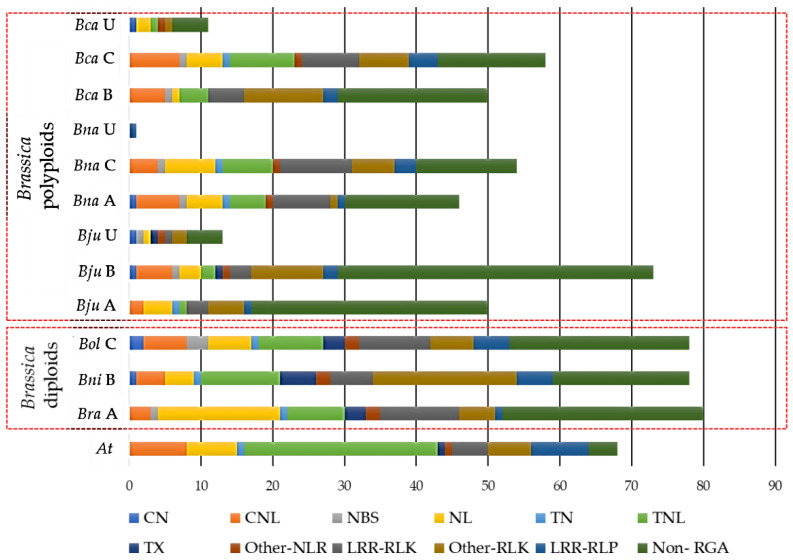
Distribution of cloned disease resistance gene homologs (CDRHs) with their resistance gene analogs (RGA) classes/subclasses (CN = coiled-coil (CC)-nucleotide-binding site (NBS), CNL = CC-NBS-leucine rice repeats (LRR), NL = NBS-LRR, TN = Toll/Interleukin-1 receptor (TIR)-LRR, TNL = Toll/Interleukin-1 receptor (TIR)-NBS-LRR, TX = Toll/Interleukin-1 receptor (TIR) with other domains, Other-NLR= NBS-LRR with other domains, LRR-RLK= LRR-receptor-like kinase proteins (RLK), Other-RLK = RLK with other domains, LRR-RLP = LRR-receptor-like proteins) and Non-RGA (CDRHs identified without RGA domain based on the RGAugury pipeline) in *Brassica rapa (Bra)*, *B. nigra (Bni)*, *B. oleracea (Bol)*, *B. juncea (Bju)*, *B. napus (Bna)*, *B. carinata (Bca)*, and *Arabidopsis thaliana (At)*. A, B, C, and U (unplaced) refer to the genome/sub-genome of *Brassica* species.

We used the sequences of the 49 cloned *R* genes to obtain CDRHs across the 7 species (Table 2). A total of 660 CDRHs, including 248 NLRs, 150 RLKs, 33 RLPs and 229 Non-RGAs (genes without RGA-related domains) were identified (Figure 1, Tables S1 and S2)*. B. juncea* contained the highest number of CDRHs with 136, followed by *B. carinata* with 119, *B. napus* with 101, *B. rapa* with 80, *B. oleracea* and *B. nigra* with 78, and *A. thaliana* with 68 (Figure 1). The total CDRHs identified in *Brassica* polyploids was 356, with an average of 119, while the total CDRHs identified in *Brassica* diploids was 236, with an average of 79 (Figure 1). On the other hand, *A. thaliana* contained less CDRHs, 68, in comparison to *Brassica* species with an average of 99 CDRHs (Figure 1).

The individual sub-genomes of each of the polyploid species contained fewer homologs and RGAs of the cloned genes than their respective A, B and C genome *Brassica* progenitors. *B. napus* and *B. juncea* had 46 CDRHs (30 RGAs, 16 non-RGAs) and 50 CDRHs (17 RGAs, 33 non-RGAs), respectively, while their A sub-genome had 80 CDRHs (52 RGAs, 28 non-RGAs). *B. juncea* and *B. carinata* had 73 CDRHs (29 RGAs, 44 non-RGAs) and 50 CDRHs (29 RGAs, 21 non-RGAs), respectively, while their B sub-genome progenitor had 78 CDRHs (59 RGAs, 19 non-RGAs) (Table S2). *B. carinata* and *B. napus* had 58 CDRHs (43 RGAs, 15 non-RGAs) and 54 CDRHs (40 RGAs, 14 non-RGAs), respectively, while their C sub-genome progenitor had 78 CDRHs (53 RGAs, 25 non-RGAs) (Table S2).

The total number of CDRHs for each disease was counted in this study. For the bacterial disease, the cloned *R* genes against *Pseudomonas syringae*, the causal agent of black leaf spot (BLS) disease, (*At_ADR1*, *At_BAK1, At_FLS2, At_NDR1, At_NRG1a, At_NRG1b, At_PBS1, At_RIN4, At_RLP30, At_RLP32, At_RPM1, At_RPS2, At_RPS4, At_RPS5, At_RRS1,* and *At_SOBIR1*) had the highest number of CDRHs; 239 (153 RGAs, 86 non-RGAs) (Table 3 and Table 4, Table S1). *B. napus* had the highest number of RGAs related to BLS resistance, 33, followed by 31 and 25 RGAs in *B. carinata* and *B. juncea*, respectively. RGAs related to BLS resistance were most observed in the C genome/sub-genome with 49 RGAs, followed by 43 and 42 RGAs in A and B genome/sub-genome, respectively. For the clone *R* gene *At_RLP1* against *Xanthomonas* spp., the causal agent of black rot (BR), 6 RGAs were only obtained in *Brassica* C genome (Table S1).

For the fungal disease, the cloned *R* genes against *Leptosphaeria maculans,* the causal agent of blackleg (BL) disease, (*Bna_MAPk, Bna_LepR3/Rlm2, Bna_Rlm9/4/7, At_RLM1a, At_RLM1b,* and *At_RLM3*), had a total of 165 CDRHs (86 RGAs, 79 non-RGAs) (Table 3 and Table 4, Table S1). Of these, 20 RGAs were found in *B. nigra*, followed by 16 RGAs in *B. carinata* (8 in B sub-genome, 7 in C sub-genome, and 1 unplaced) and 15 RGAs in *B. juncea* (4 in A sub-genome, 9 in B sub-genome, and 2 unplaced). On the other hand, the cloned *R* genes against *Albugo candida,* the causal agent of white rust (WR) disease, (*Bju_WRR1, At_RAC1, At_WRR4a, At_WRR4b, At_WRR8, At_WRR9* and *At_WRR12*), 123 CDRHs (102 RGAs, 21 non-RGAs) were identified (Table 3 and Table 4, Table S1). Of these RGAs, 22 were observed in *B. napus* (13 in the A sub-genome and 8 in the C sub-genome), followed by 19 RGAs in *B. rapa* (Table S1). A total of 109 CDRHs (95 RGAs, 14 non-RGAs) were identified to be related with cloned *R* genes against fungal pathogen *Hyaloperonospora arabidopsidis*, the causal agent of downy mildew (DM) disease, (*At_ADR1*, *NRG1a, NRG1b, At_RLP42, At_RPP1, At_RPP2a, At_RPP2B, At_RPP4, At_RPP5,* *At_RPP7, At_RPP8, At_RPP13,* and *At_RPP39*) (Table 3 and Table 4, Table S1). Of these, the 27 RGAs obtained in *A. thaliana* was the highest number of RGAs observed compared to the RGAs in other studied species (Table S1). On the other hand, the cloned *R* genes against *Plasmodiophora brassicae* (also a fungus)*,* the causal agent of clubroot (CR) disease, (*Bra_Crr1a* and *cRa/cRb*) had a total of 48 CDRHs (38 RGAs) (Table 3 and Table 4, Table S1). *B. napus* and *B. oleracea* had the highest counts with 9 RGAs (4 in the A sub-genome and 5 in the C sub-genome) and 8 RGAs, respectively. While *B. nigra* had 7 RGAs, *B. rapa* had 6 RGAs (Table S1).

We recorded a total of 75 CDRHs, including 60 RGAs, for the cloned *R* genes (*At_BAK1, At_RLP23, At_RLP30,* and *At_SOBIR1*) against fungal pathogen *Sclerotinia sclerotiorum*, the causal agent of Sclerotinia stem rot (SSR) disease (Table 3 and Table 4, Table S1). The 22 and 18 RGAs in the C and A genome/sub-genomes of the *Brassica* species, respectively, were higher compared to that of other genome/sub-genomes and *A. thaliana* (Table S1). For the cloned *R* genes against *Fusarium oxysporum* (also a fungus), the causal agent of Fusarium wilt (FW) disease (*Bol_FocBo1, At_RFO1, At_RFO2,* and *At_RFO3*), 50 CDRHs (34 RGAs, 16 non-RGAs) were obtained (Table 3 and Table 4, Table S1). *B. carinata* with 9 RGAs (4 in the B sub-genome and 5 in the C sub-genome) had the highest number, while *B. juncea* with 2 RGAs (1 in each B sub-genome and unplaced contigs) had the lowest RGA count across the studied species (Table S1).

The cloned *R* genes against fungal pathogens *Erysiphe cichoracearum* the causal agent of powdery mildew (PW) (*At_RPW8.1, At_RPW8.2* and *At_ADR1*) and *Botrytis cinerea* the causal agent of grey mould (GM) (*At_RLP42* and *At_RLM3*) were observed as having 9 CDRHs (all RGAs) and 5 CDRHs (3 RGAs, 2 non-RGAs), respectively (Table 4 and Table S1). Only the *Brassica* B and C genomes had RGAs with 3 and 2 genes, respectively for PW resistance (Table S1), while *A. thaliana* contained all 3 RGAs for GM resistance (Table S1).

### 3.2. Identification of CDRH Types

This study identified 68 CDRHs that are homologous to more than 1 of the cloned *R* genes (Table S1). Of these, 12 RGAs were previously identified and functionally characterised disease resistance genes such as *At_NRG1a, At_NRG1b*, *At_RAC1, At_WRR4b, At_WRR9, At_RLM1a, At_RPP4, At_RPP5, At_RPP2a, At_WRR8, Bra_Crr1a,* and *Bra_cRa/cRb* (Table S1). For instance, *At_WRR4b,* a WR *R* gene, and *At_RLM1b*, a BL *R* gene, were homologous to each other in this study. This was also the case with *At_RPP2a*, a DM *R* gene, and *Bol_FocBo1*, a FW *R* gene.

For the paralogous CDRHs, a total of 62 paralogs, including 43 tandem (69%) and 18 segmented (31%), were observed to the cloned *R* genes in *A. thaliana* (*At_ADR1, At_BAK1, At_FLS2, At_NDR1, NRG1a, NRG1b, At_PBS1, At_RAC1, At_RIN4, At_RFO1, At_RFO2, At_RFO3, At_RLM1a, At_RLM1b, At_RLM3, At_RPM1, At_RPP1, At_RPP2a, At_RPP2b, At_RPP4, At_RPP5, At_RPP8, At_RPP13, At_RPP39, At_RPS2, At_RPS4, At_RPS5, At_Rpw8.1, At_Rpw8.2, At_RRS1, At_SOBIR1, At_WRR4a, At_WRR4b, At_WRR8, At_WRR9,* and *At_WRR12*) (Table 3). On the other hand, 23 paralogs, including 19 segmented (83%) and 4 tandem (17%), were observed to the cloned *R* genes in *Brassica* species (*Bju_WRR1, Bna_MAPk, Bra_Crr1a, Bra_cRa/cRb, Bol_FocBo1, Bna_LepR3/Rlm2*, and *Bna_Rlm9/4/7*) (Table 3).

In terms of RGA domain retention and losses, this study found that 431 and 229 out of 660 CDRHs have retained (as RGA) and lost (as Non-RGA) resistance domains and motifs from the original gene, respectively (Table 3 and Table 4). In some cases, the RGA class of CDRHs tend to be different from their corresponding cloned gene because the RGA domain has been contracted/truncated. For example, *At_RPP8* encoding a CNL, had 4 NL and 1 CN CDRHs, while *At_WRR8* encoding a TNL, had 1 TN, 1 NL, and 1 NBS CDRHs (Table 3 and Table 4). *At_RPP8* and its CDRHs had a common NBS domain, while *At_WRR8* and its CDRHs had a common domain of either TIR, NBS or LRR (Table 3 and Table 4). On the other hand, 2 CDRHs did not have a common RGA domain with their homologous cloned *R* gene. These included *Bra_cRa/cRb* (TNL) and *Bju_WRR1* (CNL), which both had at least one Other-RLK CDRH (Table 3 and Table 4).

### 3.3. Identifying Clusters of CDRHs across Brassica Crops and Arabidopsis thaliana

**Figure 2:**
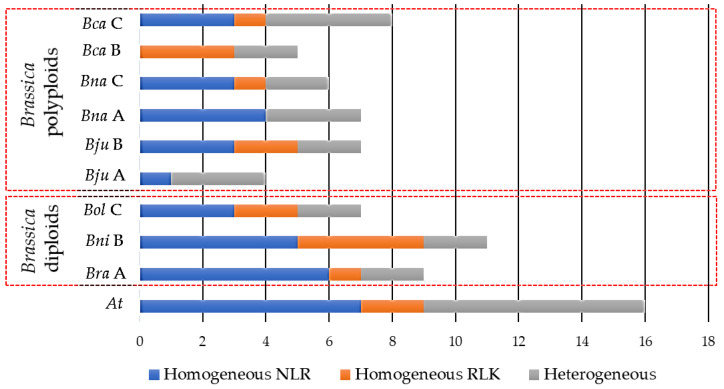
Distribution of gene clusters of cloned disease resistance homologs in *Brassica rapa* (*Bra*), *B. nigra* (*Bni*), *B. oleracea* (*Bol*), *B. juncea* (*Bju*), *B. napus* (*Bna*), *B. carinata* (*Bca*), and *Arabidopsis thaliana (At)*. NLR is nucleotide-binding site (NBS)- leucine rice repeats (LRR) while an RLK is receptor-like kinase proteins. A, B, and C refer to the genome/sub-genome of *Brassica* species.

The position of CDRHs across chromosomes of each studied species creates an opportunity to determine whether these genes form clusters. This study identified a total of 87 RGA clusters across the seven species, with the highest number of clusters observed in *A. thaliana,* 21, followed by 14, 13, 12, 11, 9, and 7 clusters in *B. carinata, B. napus*, *B. nigra, B. juncea, B. rapa*, and *B. oleracea,* respectively (Figure 2). *B. carinata, B. napus* and *B. nigra* had the highest total number of CDRH RGAs with 78, 71, and 59, respectively, while *B. rapa* and *B. oleracea* had the lowest total numbers of CDRH RGAs with 52 and 53, respectively (Table 3).

Across the studied species, 55 homogeneous RGA clusters, including 36 NLR, 16 RLK and 3 RLP homogeneous clusters, were identified (Figure 2). Within the homogeneous cluster, the cloned *R* genes *At_BAK1*, *Bra_cRa/cRb, At_FLS2, Bna_LepR3/Rlm2, At_NDR1*, *At_NRG1a,* *At_NRG1b, At_RFO1, At_RFO2, At_RLM1a, At_RLM1b, At_RPP1, At_RPP2a, At_RPP2b, At_RPP4, At_RPP5, At_RPP39, At_RPS4, At_RRS1, At_WRR4a, At_WRR4b*, *At_WRR8* and *At_WRR9* were observed to form a cluster either to their corresponding tandem paralog/s or to other functionally characterised *R* gene/s (Table S1). On the other hand, 32 heterogeneous RGA clusters were obtained (Figure 2). Aside from having cluster members with different RGA domains, a different heterogenous cluster with RGA and non-RGA members was observed (Table S1). However, the non-RGA may have a resistance-related function or partial RGA domain structure (missing some of the key domains). For example, in this study, a non-RGA AT3G20590 which is a non-specific disease resistance protein-like gene [105] clustered with the *At_NDR1* gene. Furthermore, a non-RGA, B08g104510.1, which is a mitogen-activated protein kinase [91] clustered with NLRs Bo8g104700.1, Bo8g104710.1 and Bo8g104730.1 (Table S1). Finally, the non-RGA BnaA02g24430D, which has an LRR domain [15], clustered with BnaA02g24440D, a LRR-RLP.

## 4. Discussion

RGAs are the most important genes that need to be discovered and cloned for the improvement of *Brassica* crop disease resistance. The availability of *Brassica* genomic resources, along with the model species *A. thaliana*, and the aid of computational and bioinformatic tools have led to their widespread identification. Across *A. thaliana* and *Brassica* species, an approach utilising homology can reveal associations between functionally characterised *R* genes and RGAs, and how each species’ genetic repertoire differs (for example, RGA and non-RGA content).

The larger number of total CDRHs in *Brassica* polyploids over *Brassica* diploids is likely due to polyploidisation [11,14,15]. It has previously been shown that the total number of genes, RGAs and glucosinolate-related genes in *Brassica* polyploids were higher than in the *Brassica* diploid/progenitor species [11,101]. The number of DNA transposable elements, a major factor in plant genome expansion [106], was also found to be higher in polyploid *B. napus* compared to the diploids *B. rapa* and *B. oleracea* [107], which could likely be the case for *B. carinata* and *B. juncea* when compared to their corresponding diploid progenitors. On the other hand, the fewer counts of CDRHs in *Arabidopsis* than *Brassica* species could probably be due to whole genome triplication events which did not happen in ancestral *Arabidopsis* while it occurred in ancestral *Brassica* [7,108]. As a result, it is expected that the increased genome size of *Brassicas* also increased their gene number compared to *A. thaliana* [11]. Furthermore, *Brassica* crops undergone long history of extensive breeding to improve disease resistance which may have led to an increase in their RGA content [109].

The fewer RGAs in the individual sub-genomes of the *Brassica* polyploids compared to their diploid genome progenitor that we found in this study is consistent with the other *Brassica* RGA studies [11,96,109,110,111,112]. Duplicated disease *R* genes or RGAs are favourably lost in the sub-genomes of polyploid *Brassicas* after a duplication event compared to their diploid genome progenitors [109,110,113,114]. This event was also observed in other species such as legumes [115], maize [116,117], and wheat [118]. In *B. napus*, the loss of RGAs is thought to be a result of homoeologous exchange between the A and C sub-genomes [107,119].

The total number of CDRHs, particularly the RGAs, per disease was also determined. Limited genetic resistance towards BLS, PW, and GM disease has been identified in *Brassica* species, making the RGAs obtained here a valuable starting point for future studies to explore potential BLS, PW, and GM *R* genes. For WR, it has been reported that *B. rapa* and the A sub-genome of *B. napus* are a good source of resistance [120,121,122]. The majority of markers associated with WR resistance that have been utilised for resistance exploration were also derived from the A-genome [80,123,124,125,126]. For BL, previous investigations showed that B-genome *Brassica* species have high levels of phenotypic resistance to BL compared to *Brassicas* containing the A and C genomes, and *A. thaliana* [127,128,129]. However, the association of phenotypic BL resistance to the identified RGAs in this study is yet to be confirmed. Another, QTL against FW and BR have previously been identified in *Brassica* C genome/sub-genome [89,130,131,132,133,134].

Among Brassicaceae species, *R* genes conferring resistance to DM have only been cloned in *A. thaliana* [63,64,65,66,69,70,71,79], while other *R* genes and quantitative trait loci (QTL) identified in *B. rapa* and *B. oleracea* are yet to be functionally confirmed [135,136,137,138,139]. For CR, recent studies have also shown QTL to be associated with resistance in the A and C genome of *Brassica* species [140,141,142,143,144]. *B. nigra* have also been noted with high phenotypic resistance to CR pathotypes in Canada [145], however, the RGAs obtained here need to be functionally verified with phenotypic CR resistance. Lastly, for SSR, *Brassica* A and C genomes have both been reported to harbour QTL linked to possible resistance against the disease [146,147,148,149].

Our results showed that there are a considerable number of CDRHs throughout *Brassica* crops and *A. thaliana*. CDRHs, especially those with resistance domains (RGAs), play important roles in disease resistance responses, and their subsequent application in breeding programs will help to improve disease resistance. However, RGAs are not the only genes that may confer disease resistance in *Brassica* crops and this is particularly true for diseases whose resistance response is quantitatively controlled, such as SSR [147]. Therefore, the non-RGAs identified in this study may be useful, but still need further analyses and confirmation.

A CDRH can be homologous to more than one cloned *R* gene because some of the genes may share the same resistance domains. Considerable number of collinear genes were obtained between *Arabidopsis* and *Brassica* species as they originated from one ancestral species [11,150]. It is also possible that the homology to one more gene could imply multiple resistance function. The *At_RPP8* gene, *causing* resistance to DM disease, was later found to contain two alleles; *HRT* and *RCY1,* which confer resistance to turnip crinkle virus and yellow strain cucumber mosaic virus, respectively [151,152,153]. The *At_RRS1* gene, initially associated with the avirulence gene *popP2,* which triggers resistance against *Ralstonia solanacearum* [154,155], was later found to also mediate a resistant response against *P. syringae* and *Colletotrichum higginsianum* [77]. However, this assumption needs thorough investigation and multiple functional characterisation to be confirmed.

The large number of tandem duplicates or paralogs in *A. thaliana* over *Brassica* species is consistent with findings in previous studies [13,156,157]. Tandem duplication may have occurred more frequently in *A. thaliana* because its ancestors did not undergo whole genome triplication, hence there was no extensive genome fractionation [108]. Conversely, the large number of segmented paralogs in *Brassica* species over *A. thaliana* could also be due to genome fractionation and block reshuffling which separated the homologous RGAs during the process of these evolutionary events [108,158]. Segmented paralogs act as gene-buffers in forms of structural variation such as copy number variation (CNV), which has been found abundantly in *B. napus* and *B. oleracea*, and is associated with SSR, CR and BL resistance [109,112].

Homology analysis is useful in elucidating gene gains and losses, and verifying retained resistance domains or function of genes [159]. From an ancestral gene, homologs could undergo neofunctionalisation, subfunctionalisation or duplication-degeneration-complementation (DDC), non-functionalisation or pseudogenisation, escape from adaptive conflict (EAC) and other routes involving gene dosage and redundancy [160,161,162,163,164,165,166]. In plants, RGAs are prone to rapid gene expansion during evolutionary events, as well as gene loss and contraction, as they respond to environmental stress such as disease pressure [167,168]. Nevertheless, truncated RGAs such as NL and TN have been cloned and functionally characterised with disease resistance in *A. thaliana* [53,61,62]. While a NBS gene has been reported as a signalling component in disease resistance [20], genes with TX domains were able to interact with different *R* and *Avr* genes to elicit disease resistance in *A. thaliana* [169,170], and CC domain has been reported as a candidate for the blackleg *R* genes *Rlm1, LepR2,* and *LepR4* in *B. oleracea* [171,172,173].

In gene clustering, the greater number of clusters in *A. thaliana* could possibly be due to its smaller genome size, compared to the *Brassica* species (Table 2), where the position of the genes or RGAs tend to be closer to each other. However, between *Brassica* species, the presence of RGAs could be a factor to gene clustering as it was observed in this study that the higher the total number of CDRHs with an RGA domain in each species, the higher the likelihood that these specific RGAs were part of a gene clusters.

Earlier studies have suggested homogeneous gene cluster may have evolved via tandem duplication [174,175]. The existence of tandem paralogs is yet to be functionally confirmed, however, their co-existence with cloned genes in a cluster suggests a “balancing” model in which genetic variation in disease resistance is maintained despite the presence of selection pressure [176]. In previous *A. thaliana* studies, a NLR *Suppressor of Non-expressor of Pathogenesis-Related Genes 1-1, CONSTITUTIVE 1 (SNC1)* requires its co-clustered NLR *SIDEKICK SNC1 1 (SIKIC1)*, *SIKIC2* and *SIKIC3* to mediate defence signalling [177], while the NLRs *Chilling Sensitive 1* (*CHS1)* or *TN2* pairs to *Suppressors of CHS (CHS1 or CHS2) gene 3* (*SOC3)* to monitor the homeostasis of *Senescence-Associated E3 Ubiquitin Ligase 1 (SAUL1)* [178], which is a positive regulator of PTI in plants [179]. Thus, clustering of these RGAs may be maintaining variation in disease resistance but when selection pressure occurs, RGAs could act as either accessory or helper or sensor needed in disease resistance [180,181].

The “birth and death” model could also be the fate of the RGAs in a gene cluster. The “birth and death” model indicates that when a RGA function is overcome by a pathogen, the duplication process facilitates DNA sequence exchanges of homologous genes via cross-over, leading to sequence mispairing, loss of the original sequence, converting the gene, and eventually generating a novel RGA with possible altered pathogen specificities [182]. The emergence of cloned genes *At_RPP13* in *A. thaliana*, *Pm3* in wheat, *L* in flax and *elF4E* in capsicum was said to follow the “birth and death” model [183,184,185,186]. The same mechanism of “birth and death” has likely occurred within a blackleg resistance gene *Bna_Rlm9/4/7* which contains three alleles *Rlm9*, *Rlm4* and *Rlm7* on chromosome A07 of *B. napus* [85,187] because their corresponding avirulence genes have been found to have an epistatic interaction, indicating an evolutionary arms race between the host and pathogen [188,189,190].

For the heterogeneous clusters, this clustering occurs because of random ectopic recombination, chromosomal translocation, gene transposition and co-localisation of the genes [191,192,193]. However, the genes with different domains in a cluster is yet to be functionally confirmed. The same can be said for homogenous clusters, where there is a high chance that the distribution and position of CDRHs is not random; however, this assumption requires further research for confirmation.

## 5. Conclusions

The identification of RGAs throughout the genome and underlying QTL is one of the breakthroughs that has accelerated disease resistance improvement in crops. While the process of identifying and functionally testing *R* genes has shortened, QTLs can have numerous candidates which results in time consuming validation. Hence, the use of cloned *R* gene sequences to search for RGA homologs can provide a basis for narrowing down candidates for functional characterisation.

The findings in this study can also be useful in studying the evolution and mechanisms of resistance in these genes, which can later help to guide appropriate crop methodologies to develop disease resistant and resilient *Brassica* cultivars. Additionally, gene-specific markers from these specific RGAs can be used as diagnostic markers in determining *Brassica* lines with disease resistance and possibly explore new QTL not only in *Brassica* species but in other members of the Brassicaceae family.

## Figures and Tables

**Table 1:** The 49 cloned *R* genes from *Arabidopsis thaliana (At)*, *Brassica juncea (Bju)*, *Brassica napus (Bna)* and *Brassica rapa (Bra)* used for homology searches.

Gene	RGA Type	Avirulence Gene (Pathogen)	GenBank ID	Source
*At_ADR1*	RNL	unknown *(Hyaloperonospora arabidopsidis ^F^*, *Erysiphe cichoracearum ^F^, Pseudomonas syringae ^B^)*	Q9FW44 ^U^	[32,33,34]
*At_BAK1*	LRR-RLK	*AvrPto* and *AvrPtoB* *(P. syringae)*, unknown but interacts with *RLP23/SOBIR1* interaction *(Sclerotinia sclerotiorum* *^F^)*	Q94F62 ^U^	[35,36,37,38]
*At_FLS2*	LRR-RLK	*AvrPto1 (P. syringae)*	Q9FL28 ^U^	[39,40]
*At_NDR1*	TM	*AvrRpt2 (P. syringae)*	O48915 ^U^	[41]
*At_NGR1a*	RNL	unknown *(Albugo candida ^F^, H. arabidopsidis*, and *P. syringae)*	Q9FKZ1 ^U^	[33,34]
*At_NGR1b*	RNL		Q9FKZ0 ^U^	
*At_PBS1*	STK	*AvrPphB (P. syringae)*	Q9FE20 ^U^	[42]
*At_RAC1*	TNL	unknown *(A. candida)*	Q6QX58 ^U^	[43]
*At_RIN4*	CC	* AvrB, AvrRPM1 * and *AvrRpt2* *(P. syringae)*	Q8GYN5 ^U^	[44,45,46,47,48]
*At_RFO1*	Other-RLK	unknown *(Fusarium oxysporum matthioli* *^F^)*	Q8RY17 ^U^	[49]
*At_RFO2*	LRR-RLP	unknown *(F. oxysporum matthioli)*	Q9SHI4 ^U^	[50]
*At_RFO3*	Other-RLK	unknown *(F. oxysporum matthioli)*	Q9LW83 ^U^	[51]
*At_RLM1a*	TNL	unknown *(Leptosphaeria maculans ^F^)*	Q9CAK1 ^U^	[52]
*At_RLM1b*	TNL	unknown *(L. maculans)*	F4I594 ^U^	
*At_RLM3*	TN	unknown *(L. maculans, Botrytis cinerea ^F^, Alternaria brassicicola ^F^* and *A. brassicae* *^F^)*	Q9FT77 ^U^	[53]
*At_RLP1*	LRR-RLP	unknown *(Xanthomonas* spp. *^B^)*	Q9LNV9	[54,55]
*At_RLP23*	LRR-RLP	unknown but interacts with *BAK1/SOBIR1 (S. sclerotiorum)*	O48849	[38,56]
*At_RLP30*	LRR-RLP	unknown *(P. syringae)*, interacts with *Sclerotinia culture filtrate elicitor 1 (SCFE1)/BAK1/SOBIR1 (S. sclerotiorum)*	Q9MA83	[57,58]
*At_RLP32*	LRR-RLP	unknown but interacts with *BAK1/SOBIR1 (P. syringae)*	Q9M9X0	[59]
*At_RLP42*	LRR-RLP	unknown but interacts with *SOBIR1 (B.cinerea* and *H. arabidopsidis)*	Q9LJS0	[60]
*At_RPM1*	NL	*AvrRPM1* or *AvrB (P. syringae)*	Q39214 ^U^	[61,62]
*At_RPP1*	TNL	*ATR1^NdWsB^ (H. arabidopsidis)*	F4J339 ^U^	[63]
*At_RPP2a*	TNL	unknown but interacts with *RPP2b (H. arabidopsidis)*	F4JT78 ^U^	[64]
*At_RPP2b*	TNL	unknown but interacts with *RPP2a (H. arabidopsidis)*	F4JT80 ^U^	
*At_RPP4*	TNL	*ATR4* *(H. arabidopsidis)*	F4JNA9 ^U^	[65]
*At_RPP5*	TNL	*ATR5 (H. arabidopsidis)*	F4JNB7 ^U^	[66]
*At_RPP7*	NL	unknown *(H. arabidopsidis)*	Q8W3K0 ^U^	[67,68]
*At_RPP8*	CNL	*AvrRPP8 (H. arabidopsidis)*	Q8W4J9 ^U^	[69]
*At_RPP13*	CNL	*ATR13 (H. arabidopsidis)*	Q9M667 ^U^	[70]
*At_RPP39*	CNL	*ATR39-1 (H. arabidopsidis)*	H9BPR9 ^U^	[71]
*At_RPS2*	NL	*AvrRpt2 (P. syringae)*	Q42484 ^U^	[72] [73]
*At_RPS4*	TNL	*AvrRPS4 (P. syringae)*	Q9XGM3 ^U^	
*At_RPS5*	TNL	*AvrPphB (P. syringae)*	O64973 ^U^	[74]
*At_Rpw8.1*	RNL	unknown *(E. cichoracearum)*	Q9C5Z7 ^U^	[75]
*At_Rpw8.2*	RNL	unknown *(E. cichoracearum)*	Q9C5Z6 ^U^	
*At_RRS1*	TNL	*AvrRPS4 (P. syringae), popP2 (Ralstonia solanacearum ^B^),* unknown *(Colletotrichum higginsianum ^F^)*	P0DKH5 ^U^	[76,77]
*At_SOBIR1*	LRR-RLK	unknown but interacts *FLS2 (P. syringae),* unknown but interacts with *BAK1/SOBIR1 (S. sclerotiorum)*	Q9SKB2	[35,38]
*At_WRR4a*	TNL	unknown *(A. candida)*	Q9C7X0 ^U^	[78]
*At_WRR4b*	TNL	unknown *(A. candida)*	MK034466 ^N^	[79]
*At_WRR8*	TNL	unknown *(A. candida)*	MK034463 ^N^	
*At_WRR9*	NL	unknown *(A. candida)*	MK034464 ^N^	
*At_WRR12*	TNL	unknown *(A. candida)*	MK034462 ^N^	
*Bju_WRR1*	CNL	unknown *(A. candida)*	A0A5C1IWT6 ^U^	[80]
*Bna_MPK9*	Other-RLK	*AvrLm1 (L. maculans)*	A0A078IFE9 ^U^	[81]
*Bna_LepR3/Rlm2*	LRR-RLP	*AvrLm1 (LepR3)* and *AvrLm2 (Rlm2) (L. maculans)*	I7C3X3 ^U^/ A0A0B5L618 ^U^	[82,83]
*Bna_Rlm9/4/7*	Other-RLK	*AvrLm5-9 (Rlm9)* and *AvrLm4-7 (Rlm4/7) (L. maculans)*	CDX67982.1 ^N^	[84,85]
*Bra_cRa/cRb*	TNL	unknown *(Plasmodiophora brassicae ^F^)*	M5A8J3 ^U^	[86,87]
*Bra_Crr1a*	TNL	unknown *(P. brassicae)*	AB605024.1 ^N^	[88]
*Bol_FocBo1*	TNL	unknown *(F. oxysporum* f. sp. *Conglutinans* *^F^)*	BAQ21734.1 ^N^	[89]

*^F^* = fungus, *^B^* = bacteria, RGA = resistance gene analog, TNL = Toll/Interleukin-1 receptor (TIR)-nucleotide binding site (NBS)-leucine rich repeats (LRR), TM = transmembrane, STK = Serine/threonine-specific protein kinase, Other-RLK = receptor-like kinase protein with other receptor, LRR-RLP= receptor-like proteins with LRR, TN = TIR-NBS, CNL = coiled-coil (CC)-NBS-LRR, NL = NBS-LRR, RNL = resistance to powdery mildew 8 (Rpw8)-NBS-LRR, ^U^ = https://www.uniprot.org/uniprot/, accessed on 10 October 2020) website, ^N^ = https://www.ncbi.nlm.nih.gov/ (accessed on 10 October 2020).

**Table 2:** Seven Brassicaceae species with their corresponding genome version and size used in this study.

Species	Genome Version (Size)	Source	
*Arabidopsis thaliana*	TAIR10 (119 Mbp)	https://www.arabidopsis.org/, accessed on 27 December 2020	[90]
*Brassica carinata*	zd-1 v1.0 (1087 Mbp)	http://brassicadb.bio2db.com/, accessed on 10 April 2021	[11]
*Brassica juncea*	Tumida T84-66 v1.5 (937 Mbp)	http://brassicadb.org/, accessed on 27 December 2020	[14]
*Brassica napus*	Darmor bzh v4.1 (850 Mbp)	http://brassicadb.org/, accessed on 27 December 2020	[15]
*Brassica nigra*	DH YZ12151 (402 Mbp)	http://brassicadb.org/, accessed on 27 December 2020	[14]
*Brassica oleracea*	TO100 v2.1 (488 Mbp)	http://brassicadb.org/, accessed on 27 December 2020	[91]
*Brassica rapa*	Chiifu-401-42 v3.0 (353 Mbp)	http://bigd.big.ac.cn/gwh, accessed 27 December 2020	[92]

**Table 3:** Cloned genes in *Brassica* crops and *Arabidopsis thaliana* and their corresponding paralogs (cloned disease *R* gene homolog type).

Cloned Gene (RGA Domain)		Paralog					
		T	S	RGA *		Non-RGA	Total
				Same	Different		
*Brassica* species	*Bju_WRR1* (CNL)	0	1	0	1 TX	0	1
	*Bna_MAPk* (Other-RLK)	0	8	0	0	8	8
	*Bra_cRa/cRb* (TNL)	3	1	1 TNL	1 NL, 2 TX	0	4
	*Bra_Crr1a* (TNL)	1	2	1 TNL	2 TX	1	3
	*Bol_FocBo1* (TNL)	0	0	0	0	0	0
	*Bna_LepR3/Rlm2* (LRR-RLP)	0	1	1 LRR-RLP	0	0	1
	*Bna_Rlm9/4/7* (Other-RLK)	0	6	3 Other-RLK	0	3	6
	Total	4	19	6	6	14	23
*Arabidopsis thaliana*	*At_ADR1* (NL)	0	2	2 NL	0	0	2
	*At_BAK1* (LRR-RLK)	0	4	4 LRR-RLK	0	0	4
	*At_FLS2* (LRR-RLK)	0	0	0	0	0	0
	*At_NDR1* (TM)	1	0	0	0	1	1
	*At_NRG1a* (RNL)	1	0	0	1 NL	0	1
	*At_NRG1b* (RNL)	1	0	0	1 NL	0	1
	*At_PBS1* (STK)	0	0	0	0	0	0
	*At_RAC1* (TNL)	0	4	3 TNL	1 TN	0	4
	*At_RFO1* (Other-RLK)	0	0	0	0	0	0
	*At_RFO2* (LRR-RLP)	0	1	0	1 LRR-RLK	0	1
	*At_RFO3* (Other-RLK)	0	1	1 Other-RLK	0	0	1
	*At_RIN4* (CC)	0	0	0	0	0	0
	*At_RLM1a* (TNL)	0	0	0	0	0	0
	*At_RLM1b* (TNL)	5	2	6 TNL	1 NL	0	7
	*At_RLM3* (TN)	0	0	0	0	0	0
	*At_RPM1* (NL)	0	0	0	0	0	0
	*At_RLP1* (LRR-RLP)	0	0	0	0	0	0
	*At_RLP23* (LRR-RLP)	3	0	3 LRR-RLP	0	0	3
	*At_RLP30* (LRR-RLP)	0	0	0	0	0	0
	*At_RLP32* (LRR-RLP)	0	1	1 LRR-RLP	0	0	1
	*At_RLP42* (LRR-RLP)	3	0	3 LRR-RLP	0	0	3
	*At_RPP1* (TNL)	3	3	5 TNL	1 TX	0	6
	*At_RPP2a* (TNL)	0	0	0	0	0	0
	*At_RPP2b* (TNL)	0	0	0	0	0	0
	*At_RPP4* (TNL)	6	0	5 TNL	1 Other-NLR	0	6
	*At_RPP5* (TNL)	7	0	5 TNL	1 Other-NLR	1	7
	*At_RPP5* (NL)	4	0	0	4 CNL	0	4
	*At_RPP8* (CNL)	2	0	2 CNL	0	0	2
	*At_RPP13* (CNL)	0	0	0	0	0	0
	*At_RPP39* (CNL)	3	0	2 CNL	1 NL	0	3
	*At_RPS2* (NL)	0	0	0	0	0	0
	*At_RPS4* (TNL)	1	0	1 TNL	0	0	1
	*At_RPS5* (TNL)	0	0	0	0	0	0
	*At_Rpw8.1* (RNL)	0	0	0	0	0	0
	*At_Rpw8.2* (RNL)	0	0	0	0	0	0
	*At_RRS1* (TNL)	1	0	0	1 NL	0	1
	*At_SOBIR1* (LRR-RLK)	0	0	0	0	0	0
	*At_WRR4a* (TNL)	1	0	1 TNL	0	0	1
	*At_WRR4b* (TNL)	0	0	0	0	0	0
	*At_WRR8* (TNL)	3	1	3 TNL	1 TN	0	4
	*At_WRR9* (NL)	0	1	0	0	1	1
	*At_WRR12* (TNL)	0	1	1 TNL	0	0	1
	Total	43	22	49	13	3	65

T = Tandem, S = Segmented, *Bju* = *Brassica juncea*, *Bol* = *Brassica oleracea*, *Bra* = *Brassica rapa*, *Bna* = *Brassica napus*, * Resistance gene analogs (RGA) domain in comparison to the cloned gene. CN = coiled-coil (CC)-nucleotide-binding site (NBS), CNL = CC-NBS-leucine rice repeats (LRR), NL = NBS-LRR, TN = Toll/Interleukin-1 receptor (TIR)-LRR, TNL = Toll/Interleukin-1 receptor (TIR)-NBS-LRR, TX = Toll/Interleukin-1 receptor (TIR) with other domains, Other-NLR = NBS-LRR with other domains, RNL = resistance to powdery mildew 8 (Rpw8)-NBS-LRR, LRR-RLK = LRR-receptor-like kinase proteins (RLK), STK = Serine/threonine-specific protein kinase, Other-RLK= RLK with other domains, LRR-RLP = LRR-receptor-like proteins, TM = transmembrane, Non-RGA = Homologs identified without RGA domain based on the RGAugury pipeline.

**Table 4:** Cloned genes in *Brassica* crops and *Arabidopsis thaliana* and their corresponding orthologs (cloned disease *R* gene homolog type).

Cloned Gene (RGA Domain)		Ortholog			
		RGA *		Non-RGA	Total
		Same	Different		
*Brassica* species	*Bju_WRR1* (CNL)	14 CNL	12 NL, 3 CN, 1 Other-RLK	16	46
	*Bna_MAPk* (Other-RLK)	1 Other-RLK	0	31	32
	*Bra_cRa/cRb* (TNL)	9 TNL	4 Other-NLR, 12 TN, 1 Other-RLK, 3 TX	1	19
	*Bra_Crr1a* (TNL)	9 TNL	1 NL, 1 Other-NLR, 1 CNL, 2 TX	8	22
	*Bol_FocBo1* (TNL)	7 TNL	1 Other-NLR, 1 TN, 1 TX	5	15
	*Bna_LepR3/Rlm2* (LRR-RLP)	4 LRR-RLP	0	3	7
	*Bna_Rlm9/4/7* (Other-RLK)	56 Other-RLK	0	28	84
	Total	100	94	94	229
*Arabidopsis thaliana*	*At_ADR1* (NL)	2 NL	3 CNL	0	5
	*At_BAK1* (LRR-RLK)	30 LRR-RLK	0	11	41
	*At_FLS2* (LRR-RLK)	13 LRR-RLK	1 LRR-RLP	5	19
	*At_NDR1* (TM)	0	0	22	22
	*At_NRG1a* (RNL)	0	6 CNL, 1 LRR-RLP, 1 NBS, 17 NL	4	29
	*At_NRG1b* (RNL)	0	8 CNL, 1 LRR-RLP, 1 NBS, 16 NL	4	29
	*At_PBS1* (STK)	0	1 NL	36	37
	*At_RAC1* (TNL)	0	1 NBS	0	1
	*At_RFO1* (Other-RLK)	2 Other-RLK	0	1	3
	*At_RFO2* (LRR-RLP)	4 LRR-RLP	0	3	7
	*At_RFO3* (Other-RLK)	15 Other-RLK	1 Other-NLR	6	22
	*At_RIN4* (CC)	0	0	0	0
	*At_RLM1a* (TNL)	0	0	0	0
	*At_RLM1b* (TNL)	9 TNL	3 NL, 1 Other-NLR, 2 TN, 1 TX	6	22
	*At_RLM3* (TN)	0	0	0	0
	*At_RPM1* (NL)	4 NL	1 NBS	5	10
	*At_RLP1* (LRR-RLP)	7 LRR-RLP	0	4	11
	*At_RLP23* (LRR-RLP)	0	0	2	2
	*At_RLP30* (LRR-RLP)	0	0	0	0
	*At_RLP32* (LRR-RLP)	4 LRR-RLP	0	2	6
	*At_RLP42* (LRR-RLP)	0	0	2	2
	*At_RPP1* (TNL)	0	0	0	0
	*At_RPP2a* (TNL)	6 TNL	1 NBS	2	9
	*At_RPP2b* (TNL)	6 TNL	1 NBS	4	11
	*At_RPP4* (TNL)	0	0	0	0
	*At_RPP5* (TNL)	1 TNL	0	0	1
	*At_RPP5* (NL)	0	0	0	0
	*At_RPP8* (CNL)	0	4 NL, 1 CN	0	5
	*At_RPP13* (CNL)	1 CNL	2 CN, 2 NBS, 1 NL	3	9
	*At_RPP39* (CNL)	1 CNL	1 CN	0	2
	*At_RPS2* (NL)	2 NL	7 CNL, 1 LRR-RLK	1	11
	*At_RPS4* (TNL)	6 TNL	1 NL, 1 TN, 1 LRR-RLP	2	11
	*At_RPS5* (TNL)	0	0	0	0
	*At_Rpw8.1* (RNL)	0	0	0	0
	*At_Rpw8.2* (RNL)	0	0	0	0
	*At_RRS1* (TNL)	1 TNL	2 NL	0	3
	*At_SOBIR1* (LRR-RLK)	21 LRR-RLK	2 LRR-RLP	2	23
	*At_WRR4a* (TNL)	0	0	0	0
	*At_WRR4b* (TNL)	0	0	0	0
	*At_WRR8* (TNL)	0	1 NL, 1 NBS	0	2
	*At_WRR9* (NL)	0	0	0	0
	*At_WRR12* (TNL)	7 TNL	5 Other-NLR, 3 NBS, 3 NL, 1 TN, 3 TX, 1 LRR-RLP	1	24
	Total	144	61	120	323

T = Tandem, S = Segmented, *Bju* = *Brassica juncea*, *Bol* = *Brassica oleracea*, *Bra* = *Brassica rapa*, *Bna* = *Brassica napus*, * Resistance gene analogs (RGA) domain in comparison to the cloned gene. CN = coiled-coil (CC)-nucleotide-binding site (NBS), CNL = CC-NBS-leucine rice repeats (LRR), NL = NBS-LRR, TN = Toll/Interleukin-1 receptor (TIR)-LRR, TNL = Toll/Interleukin-1 receptor (TIR)-NBS-LRR, TX = Toll/Interleukin-1 receptor (TIR) with other domains, Other-NLR = NBS-LRR with other domains, RNL = resistance to powdery mildew 8 (Rpw8)-NBS-LRR, LRR-RLK = LRR-receptor-like kinase proteins (RLK), STK = Serine/threonine-specific protein kinase, Other-RLK = RLK with other domains, LRR-RLP = LRR-receptor-like proteins, TM = transmembrane, Non-RGA = Homologs identified without RGA domain based on the RGAugury pipeline.

## Data Availability

The data used in this research is publicly available. The gene sequences can be found at https://www.uniprot.org/uniprot/ (accessed on 10 October 2020) and https://www.ncbi.nlm.nih.gov/ (accessed on 10 October 2020) while the genomic databases can be found at https://www.arabidopsis.org/ (accessed on 27 December 2020), http://brassicadb.org/ (accessed on 27 December 2020), http://bigd.big.ac.cn/gwh (accessed 27 December 2020), and http://brassicadb.bio2db.com/ (accessed on 10 April 2021). The data (results) presented in this research are available in the Supplementary Materials.

## Supplementary Materials

The following supporting information can be downloaded at: https://www.mdpi.com/article/10.3390/biology11060821/s1, Table S1: Results from the BLAST analysis (Comparative Genomics website) along with the identification of resistance gene analogs (RGAs) using RGAugury pipeline in Brassicaceae species and the underlying types of homolog; Table S2: List of resistance gene analogs (RGAs) in *Brassica carinata* zd-1 v1.0 identified by RGAugury pipeline.
