# Supplementary material for: Mining of Cloned Disease Resistance Gene Homologs (CDRHs) in Brassica Species and Arabidopsis thaliana
Source: Biology (Basel). 2022 May 26;11(6):821. doi: 10.3390/biology11060821 (PMC9220128; doi:10.3390/biology11060821)
Supplement: Supplementary file 1 [file biology-11-00821-s001.zip › Table S1.pdf]

Table S1. Results from the BLAST analysis (Comparative Genomics website) along with the identification of resistance gene analogs (RGAs) using RGAugury pipeline in Brassicacaeae species and the underlying types of homolog.

Hit number	Cloned gene (RGA domain class)	Organism	Chromosome	Position (bp)	Length (aa)	E-value	Perc ID	Score	Gene database name	Based on RGAugury pipeline	Gene and the homolog with its type and subtypes	RGA subclass in comparison to the cloned gene	Cluster number in their respective species
1	At_RLP1 (LRR-RLP)	Arabidopsis thaliana (vTAIR10)	At01	2,269,764	146	2.00E-80	100%	293	AT1G07390	LRR-RLP	At_RLP1 gene		
2	At_RLP1 (LRR-RLP)	Arabidopsis thaliana (vTAIR10)	At01	2,272,450	734	0	100%	1395	AT1G07390	LRR-RLP	At_RLP1 gene		
3	At_RPS5 (CNL)	Arabidopsis thaliana (vTAIR10)	At01	4,144,788	1050	0	100%	1880	AT1G12220	CNL	At_RPS5 gene		
4	Bna_Rlm9/4/7 (Other-RLK)	Arabidopsis thaliana (vTAIR10)	At01	5,523,853	359	1.00E-133	70.40%	446	AT1G16120	Other-RLK	Bna_Rlm9/4/7 ortholog	same	1
5	Bna_Rlm9/4/7 (Other-RLK)	Arabidopsis thaliana (vTAIR10)	At01	5,537,187	346	1.00E-126	70.50%	425	AT1G16160	Other-RLK	Bna_Rlm9/4/7 ortholog	same	1
6	At_RFO2 (LRR-RLP)	Arabidopsis thaliana (vTAIR10)	At01	5,903,439	756	0	100%	1407	AT1G17250	LRR-RLP	At_RFO2 gene		2
7	At_WRR12 (TNL)	Arabidopsis thaliana (vTAIR10)	At01	6,054,380	367	0	99.40%	671	AT1G17600	TNL	At_WRR12 gene		2
8	At_WRR12 (TNL)	Arabidopsis thaliana (vTAIR10)	At01	6,058,114	1069	0	96.80%	1971	AT1G17600	TNL	At_WRR12 gene		3
9	Bna_Rlm9/4/7 (Other-RLK)	Arabidopsis thaliana (vTAIR10)	At01	6,160,467	358	2.00E-133	70.60%	445	AT1G17910	Other-RLK	Bna_Rlm9/4/7 ortholog	same	3
10	At_RAC1 (TNL)	Arabidopsis thaliana (vTAIR10)	At01	11,291,512	889	0	96.60%	1384	AT1G31540	TNL	At_RAC1 gene		
11	At_RAC1 (TNL)	Arabidopsis thaliana (vTAIR10)	At01	11,293,156	388	0	97.10%	709	AT1G31540	TNL	At_RAC1 gene		
12	At_WRR8 (TNL)	Arabidopsis thaliana (vTAIR10)	At01	11,293,156	370	0	73.70%	521	AT1G31540	TNL	At_WRR8 paralog (segmented)	same	
13	At_ADR1 (NL)	Arabidopsis thaliana (vTAIR10)	At01	12,169,092	590	0	86.90%	993	AT1G33560	NL	At_ADR1 gene		
14	At_ADR1 (NL)	Arabidopsis thaliana (vTAIR10)	At01	12,171,051	275	2.00E-169	100%	549	AT1G33560	NL	At_ADR1 gene		
15	At_BAK1 (LRR-RLK)	Arabidopsis thaliana (vTAIR10)	At01	12,461,354	337	6.00E-167	73.20%	488	AT1G34210	LRR-RLK	At_BAK1 paralog (segmented)	same	
16	At_WRR4a (TNL)	Arabidopsis thaliana (vTAIR10)	At01	21,167,565	203	1.00E-112	100%	395	AT1G56510	TNL	At_WRR4a gene		4
17	At_WRR4a (TNL)	Arabidopsis thaliana (vTAIR10)	At01	21,169,503	365	0	100%	661	AT1G56510	TNL	At_WRR4a gene		4
18	At_WRR4a (TNL)	Arabidopsis thaliana (vTAIR10)	At01	21,171,093	480	0	99.70%	729	AT1G56510	TNL	At_WRR4a gene		4
19	At_WRR4a (TNL)	Arabidopsis thaliana (vTAIR10)	At01	21,179,029	202	2.00E-74	76.70%	275	AT1G56520	TNL	At_WRR4a paralog (tandem)	same	4
20	At_WRR4b (TNL)	Arabidopsis thaliana (vTAIR10)	At01	21,181,585	185	4.00E-98	100%	350	AT1G56540	TNL	At_WRR4b gene		4
21	At_RLM1b (TNL)	Arabidopsis thaliana (vTAIR10)	At01	21,181,694	149	4.00E-65	74.40%	245	AT1G56540	TNL	At_RLM1b paralog (tandem)	same	4
22	At_WRR4b (TNL)	Arabidopsis thaliana (vTAIR10)	At01	21,182,268	495	0	92.70%	721	AT1G56540	TNL	At_WRR4b gene		4
23	At_WRR4b (TNL)	Arabidopsis thaliana (vTAIR10)	At01	21,183,881	538	0	99.80%	838	AT1G56540	TNL	At_WRR4b gene		4
24	At_RPP7 (NL)	Arabidopsis thaliana (vTAIR10)	At01	21,760,167	375	0	92%	648	AT1G58602	NL	At_RPP7 gene		5
25	At_RPP7 (NL)	Arabidopsis thaliana (vTAIR10)	At01	21,761,381	794	0	99.80%	1445	AT1G58602	NL	At_RPP7 gene		5
26	At_RPP7 (NL)	Arabidopsis thaliana (vTAIR10)	At01	21,781,760	639	0	82.10%	925	AT1G58807	CNL	At_RPP7 paralog (tandem)	different	5
27	At_RPP7 (NL)	Arabidopsis thaliana (vTAIR10)	At01	21,793,326	682	0	81.80%	930	AT1G58848	CNL	At_RPP7 paralog (tandem)	different	5
28	At_RPP7 (NL)	Arabidopsis thaliana (vTAIR10)	At01	21,818,018	639	0	82.10%	925	AT1G59124	CNL	At_RPP7 paralog (tandem)	different	5
29	At_RPP7 (NL)	Arabidopsis thaliana (vTAIR10)	At01	21,829,584	682	0	81.80%	930	AT1G59218	CNL	At_RPP7 paralog (tandem)	different	5
30	At_RPP39 (CNL)	Arabidopsis thaliana (vTAIR10)	At01	22,551,486	876	0	87.80%	1374	AT1G61180	CNL	At_RPP39 paralog (tandem)	same	6
31	At_RPP39 (CNL)	Arabidopsis thaliana (vTAIR10)	At01	22,557,602	881	0	89.70%	1423	AT1G61190	CNL	At_RPP39 gene		6
32	At_RPP39 (CNL)	Arabidopsis thaliana (vTAIR10)	At01	22,610,076	729	0	87.90%	1165	AT1G61300	NL	At_RPP39 paralog (tandem)	different	6
33	At_RPP39 (CNL)	Arabidopsis thaliana (vTAIR10)	At01	22,615,943	885	0	88.20%	1400	AT1G61310	CNL	At_RPP39 paralog (tandem)	same	6
34	At_RLM1b (TNL)	Arabidopsis thaliana (vTAIR10)	At01	23,641,794	149	5.00E-62	72.40%	235	AT1G63730	TNL	At_RLM1b paralog (tandem)	same	7
35	At_WRR9 (NL)	Arabidopsis thaliana (vTAIR10)	At01	23,650,848	955	0	97.40%	1474	AT1G63750	NL	At_WRR9 gene		7
36	At_RLM1b (TNL)	Arabidopsis thaliana (vTAIR10)	At01	23,650,979	149	4.00E-60	71.80%	229	AT1G63750	NL	At_RLM1b paralog (tandem)	different	7
37	At_WRR9 (NL)	Arabidopsis thaliana (vTAIR10)	At01	23,653,817	528	0	99.80%	872	AT1G63750	NL	At_WRR9 gene		7
38	At_RLM1b (TNL)	Arabidopsis thaliana (vTAIR10)	At01	23,705,272	176	7.00E-136	74.40%	253	AT1G63860	TNL	At_RLM1b paralog (tandem)	same	7
39	At_RLM1b (TNL)	Arabidopsis thaliana (vTAIR10)	At01	23,713,692	488	0	100%	986	AT1G63880	TNL	At_RLM1b gene		7
40	At_RLM1b (TNL)	Arabidopsis thaliana (vTAIR10)	At01	23,715,323	512	0	91.70%	957	AT1G63880	TNL	At_RLM1b gene		7
41	At_RLM1b (TNL)	Arabidopsis thaliana (vTAIR10)	At01	23,716,206	211	1.00E-114	100%	400	AT1G63880	TNL	At_RLM1b gene		7
42	At_RLM1a (TNL)	Arabidopsis thaliana (vTAIR10)	At01	23,779,927	165	5.00E-77	100%	281	AT1G64070	TNL	At_RLM1a gene		7
43	At_RLM1b (TNL)	Arabidopsis thaliana (vTAIR10)	At01	23,779,976	149	2.00E-63	72.40%	239	AT1G64070	TNL	At_RLM1b paralog (tandem)	same	7
44	At_RLM1b (TNL)	Arabidopsis thaliana (vTAIR10)	At01	23,780,666	370	0	72.90%	553	AT1G64070	TNL	At_RLM1b paralog (tandem)	same	7
45	At_RLM1a (TNL)	Arabidopsis thaliana (vTAIR10)	At01	23,780,674	364	0	100%	634	AT1G64070	TNL	At_RLM1a gene		7
46	At_RLM1a (TNL)	Arabidopsis thaliana (vTAIR10)	At01	23,782,312	379	0	99.70%	694	AT1G64070	TNL	At_RLM1a gene		7
47	Bra_Crr1a (TNL)	Arabidopsis thaliana (vTAIR10)	At01	24,494,968	149	2.00E-63	74.40%	241	AT1G65850	TNL	Bra_Crr1a ortholog	same	
48	At_RFO3 (Other-RLK)	Arabidopsis thaliana (vTAIR10)	At01	25,303,681	291	1.00E-160	72.10%	385	AT1G67520	Other-RLK	At_RFO3 paralog (segmented)	same	
49	Bna_Rlm9/4/7 (Other-RLK)	Arabidopsis thaliana (vTAIR10)	At01	26,229,956	418	0	82.20%	584	AT1G69730	Other-RLK	Bna_Rlm9/4/7 ortholog	same	
50	At_BAK1 (LRR-RLK)	Arabidopsis thaliana (vTAIR10)	At01	27,020,599	282	4.00E-163	85.10%	485	AT1G71830	LRR-RLK	At_BAK1 paralog (segmented)	same	
51	At_RFO2 (LRR-RLP)	Arabidopsis thaliana (vTAIR10)	At01	27,220,921	700	0	71.40%	940	AT1G72300	LRR-RLK	At_RFO2 paralog (segmented)	different	
52	At_RFO1 (Other-RLK)	Arabidopsis thaliana (vTAIR10)	At01	29,979,356	877	0	92.40%	1398	AT1G79670	Other-RLK	At_RFO1 gene		8
53	Bna_Rlm9/4/7 (Other-RLK)	Arabidopsis thaliana (vTAIR10)	At01	29,981,411	409	0	84.30%	594	AT1G79680	Other-RLK	Bna_Rlm9/4/7 ortholog	same	8
54	Bna_MAPk (Other-RLK)	Arabidopsis thaliana (vTAIR10)	At02	202,076	160	2.00E-105	73.70%	208	AT2G01450		Bna_MAPk ortholog	lost	
55	At_BAK1 (LRR-RLK)	Arabidopsis thaliana (vTAIR10)	At02	5,745,772	269	2.00E-174	73.90%	385	AT2G13790	LRR-RLK	At_BAK1 paralog (segmented)	same	9
56	At_BAK1 (LRR-RLK)	Arabidopsis thaliana (vTAIR10)	At02	5,756,256	269	3.00E-162	73.60%	382	AT2G13800	LRR-RLK	At_BAK1 paralog (segmented)	same	9
57	At_RPP1 (TNL)	Arabidopsis thaliana (vTAIR10)	At02	5,925,381	148	4.00E-65	75.60%	245	AT2G14080	TNL	At_RPP1 paralog (segmented)	same	9
58	Bna_LepR3/Rlm2 (LRR-RLP)	Arabidopsis thaliana (vTAIR10)	At02	6,511,232	360	4.00E-158	71.90%	478	AT2G15042	LRR-RLP	Bna_LepR3/Rlm2 ortholog	same	
59	At_RLM1b (TNL)	Arabidopsis thaliana (vTAIR10)	At02	7,311,157	373	0	70.70%	533	AT2G16870	TNL	At_RLM1b paralog (segmented)	same	
60	At_RLP23 (LRR-RLP)	Arabidopsis thaliana (vTAIR10)	At02	10,827,240	726	0	71.20%	865	AT2G25440	LRR-RLP	At_RLP23 paralog (tandem)	same	
61	At_SOBIR1 (LRR-RLK)	Arabidopsis thaliana (vTAIR10)	At02	13,554,920	641	0	100%	1187	AT2G31880	LRR-RLK	At_SOBIR1 gene		10
62	At_RLP23 (LRR-RLP)	Arabidopsis thaliana (vTAIR10)	At02	13,862,545	867	0	100%	1505	AT2G32680	LRR-RLP	At_RLP23 gene		either 10 or 11
63	At_RLP23 (LRR-RLP)	Arabidopsis thaliana (vTAIR10)	At02	14,016,435	867	0	72.40%	1080	AT2G33020	LRR-RLP	At_RLP23 paralog (tandem)	same	11
64	At_RLP23 (LRR-RLP)	Arabidopsis thaliana (vTAIR10)	At02	14,018,361	230	1.00E-90	71.30%	321	AT2G33030	LRR-RLP	At_RLP23 paralog (tandem)	same	11
65	At_RLP30 (LRR-RLP)	Arabidopsis thaliana (vTAIR10)	At03	1,533,260	786	0	100%	1396	AT3G05360	LRR-RLP	At_RLP30 gene		12
66	At_RLP32 (LRR-RLP)	Arabidopsis thaliana (vTAIR10)	At03	1,648,490	868	0	100%	1426	AT3G05650	LRR-RLP	At_RLP32 gene		12
67	At_RPM1 (NL)	Arabidopsis thaliana (vTAIR10)	At03	2,229,555	1233	0	100%	2282	AT3G07040	NL	At_RPM1 gene		
68	At_RFO3 (Other-RLK)	Arabidopsis thaliana (vTAIR10)	At03	5,439,609	489	0	88.30%	820	AT3G16030	Other-RLK	At_RFO3 gene		
69	At_RFO3 (Other-RLK)	Arabidopsis thaliana (vTAIR10)	At03	5,441,377	266	0	82.30%	395	AT3G16030	Other-RLK	At_RFO3 gene		
70	At_RFO3 (Other-RLK)	Arabidopsis thaliana (vTAIR10)	At03	5,442,254	246	0	89.80%	397	AT3G16030	Other-RLK	At_RFO3 gene		
71	At_NDR1 (TM)	Arabidopsis thaliana (vTAIR10)	At03	7,191,686	255	1.00E-97	70.90%	317	AT3G20590		At_NDR1 paralog (tandem)	lost	13
72	At_NDR1 (TM)	Arabidopsis thaliana (vTAIR10)	At03	7,194,877	219	2.00E-140	100%	440	AT3G20600		At_NDR1 gene		13
73	At_RLP42 (LRR-RLP)	Arabidopsis thaliana (vTAIR10)	At03	9,101,765	859	0	83.10%	1154	AT3G24900	LRR-RLP	At_RLP42 paralog (tandem)	same	14
74	At_RLP42 (LRR-RLP)	Arabidopsis thaliana (vTAIR10)	At03	9,108,775	871	0	92.30%	1317	AT3G24982	LRR-RLP	At_RLP42 paralog (tandem)	same	14
75	At_RLP42 (LRR-RLP)	Arabidopsis thaliana (vTAIR10)	At03	9,112,679	868	0	85.30%	1227	AT3G25010	LRR-RLP	At_RLP42 paralog (tandem)	same	14
76	At_RLP42 (LRR-RLP)	Arabidopsis thaliana (vTAIR10)	At03	9,119,471	867	0	100%	1454	AT3G25020	LRR-RLP	At_RLP42 gene		14
77	At_RIN4 (CC)	Arabidopsis thaliana (vTAIR10)	At03	9,132,936	226	7.00E-77	88.40%	257	AT3G25070		At_RIN4 gene		14
78	At_RPP1 (TNL)	Arabidopsis thaliana (vTAIR10)	At03	9,268,740	178	1.00E-71	71.30%	266	AT3G25510	TNL	At_RPP1 paralog (segmented)	same	14
79	At_RPP1 (TNL)	Arabidopsis thaliana (vTAIR10)	At03	16,049,539	560	0	78.20%	862	AT3G44400	TNL	At_RPP1 paralog (tandem)	same	15
80	At_RPP1 (TNL)	Arabidopsis thaliana (vTAIR10)	At03	16,093,505	456	0	99.70%	884	AT3G44480	TNL	At_RPP1 gene		15
81	At_RPP1 (TNL)	Arabidopsis thaliana (vTAIR10)	At03	16,096,041	684	0	89.40%	1190	AT3G44480	TNL	At_RPP1 gene		15
82	At_RPP1 (TNL)	Arabidopsis thaliana (vTAIR10)	At03	16,196,292	711	0	81.40%	1090	AT3G44630	TNL	At_RPP1 paralog (tandem)	same	15
83	At_RPP1 (TNL)	Arabidopsis thaliana (vTAIR10)	At03	16,198,893	460	0	75.80%	625	AT3G44630	TNL	At_RPP1 paralog (tandem)	same	15
84	At_RPP1 (TNL)	Arabidopsis thaliana (vTAIR10)	At03	16,217,520	599	0	83.30%	957	AT3G44670	TNL	At_RPP1 paralog (tandem)	same	15
85	At_RPP13 (CNL)	Arabidopsis thaliana (vTAIR10)	At03	17,133,246	835	0	100%	1493	AT3G46530	CNL	At_RPP13 gene		
86	At_RPW8.1 (RNL)	Arabidopsis thaliana (vTAIR10)	At03	18,729,146	102	1.00E-13	45%	70.5	AT3G50470		At_RPW8.1 gene		
87	At_RPW8.2 (RNL)	Arabidopsis thaliana (vTAIR10)	At03	18,729,152	100	9.00E-22	68%	95.9	AT3G50470		At_RPW8.2 gene		
88	At_RPW8.1 (RNL)	Arabidopsis thaliana (vTAIR10)	At03	18,729,800	51	2.00E-07	50.90%	51.2	AT3G50470		At_RPW8.1 gene		
89	At_RPW8.2 (RNL)	Arabidopsis thaliana (vTAIR10)	At03	18,729,812	51	8.00E-13	74.50%	68.6	AT3G50470		At_RPW8.2 gene		
90	At_RLM1b (TNL)	Arabidopsis thaliana (vTAIR10)	At04	8,283,365	149	0	71.80%	239	AT4G14370	TNL	At_RLM1b paralog (segmented)	same	
91	At_RPP5 (TNL)	Arabidopsis thaliana (vTAIR10)	At04	9,483,820	178	5.00E-92	78.60%	284	AT4G16855		At_RPP5 paralog (tandem)	lost	16
92	At_RPP5 (TNL)	Arabidopsis thaliana (vTAIR10)	At04	9,489,222	181	2.00E-80	84.50%	296	AT4G16860	TNL	At_RPP5 paralog (tandem)	same	16
93	At_RPP4 (TNL)	Arabidopsis thaliana (vTAIR10)	At04	9,489,258	216	0	100%	437	AT4G16860	TNL	At_RPP4 gene		16
94	At_RPP4 (TNL)	Arabidopsis thaliana (vTAIR10)	At04	9,489,854	161	0	100%	340	AT4G16860	TNL	At_RPP4 gene		16
95	At_RPP5 (TNL)	Arabidopsis thaliana (vTAIR10)	At04	9,489,854	161	3.00E-166	81.90%	275	AT4G16860	TNL	At_RPP5 paralog (tandem)	same	16
96	At_RPP4 (TNL)	Arabidopsis thaliana (vTAIR10)	At04	9,492,071	352	0	100%	672	AT4G16860	TNL	At_RPP4 gene		16
97	At_RPP5 (TNL)	Arabidopsis thaliana (vTAIR10)	At04	9,492,071	359	2.00E-176	82.40%	593	AT4G16860	TNL	At_RPP5 paralog (tandem)	same	16
98	At_RPP5 (TNL)	Arabidopsis thaliana (vTAIR10)	At04	9,495,670	151	8.00E-65	78.80%	246	AT4G16860	TNL	At_RPP5 paralog (tandem)	same	16
99	At_RPP4 (TNL)	Arabidopsis thaliana (vTAIR10)	At04	9,503,269	161	3.00E-92	84.40%	276	AT4G16890	Other-NLR	At_RPP4 paralog (tandem)	different	16
100	At_RPP5 (TNL)	Arabidopsis thaliana (vTAIR10)	At04	9,503,269	161	2.00E-97	88.10%	298	AT4G16890	Other-NLR	At_RPP5 paralog (tandem)	different	16
101	At_RPP4 (TNL)	Arabidopsis thaliana (vTAIR10)	At04	9,504,913	352	0	78.60%	525	AT4G16890	Other-NLR	At_RPP4 paralog (tandem)	different	16
102	At_RPP5 (TNL)	Arabidopsis thaliana (vTAIR10)	At04	9,504,913	359	0	86.90%	630	AT4G16890	Other-NLR	At_RPP5 paralog (tandem)	different	16
103	Bol_FocBo1 (TNL)	Arabidopsis thaliana (vTAIR10)	At04	9,505,437	150	4.00E-156	74.00%	200	AT4G16890	Other-NLR	Bol_FocBo1 ortholog	different	16
104	At_RPP4 (TNL)	Arabidopsis thaliana (vTAIR10)	At04	9,513,584	196	1.00E-76	73.40%	281	AT4G16900	TNL	At_RPP4 paralog (tandem)	same	16
105	At_RPP5 (TNL)	Arabidopsis thaliana (vTAIR10)	At04	9,516,514	546	0	72.70%	764	AT4G16900	TNL	At_RPP5 paralog (tandem)	same	16
106	At_RPP5 (TNL)	Arabidopsis thaliana (vTAIR10)	At04	9,520,891	566	0	79.60%	809	AT4G16920	TNL	At_RPP5 paralog (tandem)	same	16
107	At_RPP5 (TNL)	Arabidopsis thaliana (vTAIR10)	At04	9,521,563	160	1.00E-159	100%	341	AT4G16920	TNL	At_RPP5 paralog (tandem)	same	16
108	At_RPP4 (TNL)	Arabidopsis thaliana (vTAIR10)	At04	9,525,078	352	0	93.70%	632	AT4G16920	TNL	At_RPP4 paralog (tandem)	same	16
109	At_RPP5 (TNL)	Arabidopsis thaliana (vTAIR10)	At04	9,525,078	359	1.00E-180	83.50%	605	AT4G16920	TNL	At_RPP5 paralog (tandem)	same	16
110	At_RPP5 (TNL)	Arabidopsis thaliana (vTAIR10)	At04	9,525,664	148	1.00E-59	75.60%	229	AT4G16920	TNL	At_RPP5 paralog (tandem)	same	16
111	At_RPP4 (TNL)	Arabidopsis thaliana (vTAIR10)	At04	9,537,014	355	1.00E-154	78.50%	519	AT4G16940	TNL	At_RPP4 paralog (tandem)	same	16

112	At_RPP5 (TNL)	Arabidopsis thaliana (vTAIR10)	At04	9,537,014	363	3.00E-162	77.60%	550	AT4G16940	TNL	At_RPP5 paralog (tandem)	same	16
113	At_RPP4 (TNL)	Arabidopsis thaliana (vTAIR10)	At04	9,537,612	149	3.00E-82	95.30%	300	AT4G16940	TNL	At_RPP4 paralog (tandem)	same	16
114	At_RPP5 (TNL)	Arabidopsis thaliana (vTAIR10)	At04	9,537,612	153	4.00E-65	79.70%	247	AT4G16940	TNL	At_RPP5 paralog (tandem)	same	16
115	At_RPP5 (TNL)	Arabidopsis thaliana (vTAIR10)	At04	9,540,882	459	0	99.50%	927	AT4G16950	TNL	At_RPP5 gene		16
116	At_RPP5 (TNL)	Arabidopsis thaliana (vTAIR10)	At04	9,541,554	160	0	100%	341	AT4G16950	TNL	At_RPP5 gene		16
117	At_RPP4 (TNL)	Arabidopsis thaliana (vTAIR10)	At04	9,544,313	545	0	74.80%	764	AT4G16950	TNL	At_RPP4 paralog (tandem)	same	16
118	At_RPP5 (TNL)	Arabidopsis thaliana (vTAIR10)	At04	9,544,319	553	0	92.40%	1052	AT4G16950	TNL	At_RPP5 gene		16
119	At_RPP4 (TNL)	Arabidopsis thaliana (vTAIR10)	At04	9,550,379	355	1.00E-154	78.50%	519	AT4G16960	TNL	At_RPP4 paralog (tandem)	same	16
120	At_RPP4 (TNL)	Arabidopsis thaliana (vTAIR10)	At04	9,550,977	149	4.00E-82	95.30%	299	AT4G16960	TNL	At_RPP4 paralog (tandem)	same	16
121	At_RPP5 (TNL)	Arabidopsis thaliana (vTAIR10)	At04	9,550,977	153	6.00E-65	79.70%	246	AT4G16960	TNL	At_RPP5 paralog (tandem)	same	16
122	At_RLM3 (TN)	Arabidopsis thaliana (vTAIR10)	At04	9,560,174	148	8.00E-75	99.30%	273	AT4G16990	TN	At_RLM3 gene		16
123	At_RLM3 (TN)	Arabidopsis thaliana (vTAIR10)	At04	9,561,087	279	2.00E-146	98.90%	420	AT4G16990	TN	At_RLM3 gene		16
124	At_RLM3 (TN)	Arabidopsis thaliana (vTAIR10)	At04	9,562,675	175	2.00E-54	73.70%	208	AT4G16990	TN	At_RLM3 gene		16
125	At_RLM3 (TN)	Arabidopsis thaliana (vTAIR10)	At04	9,564,471	157	8.00E-89	98.70%	316	AT4G16990	TN	At_RLM3 gene		16
126	Bol_FocBo1 (TNL)	Arabidopsis thaliana (vTAIR10)	At04	10,625,808	148	8.00E-127	70.20%	198	AT4G19500	TNL	Bol_FocBo1 ortholog	same	17
127	At_RPP2a (TNL)	Arabidopsis thaliana (vTAIR10)	At04	10,626,346	565	0	100%	1055	AT4G19500	TNL	At_RPP2a gene		17
128	At_RPP2a (TNL)	Arabidopsis thaliana (vTAIR10)	At04	10,628,163	363	0	96.40%	704	AT4G19500	TNL	At_RPP2a gene		17
129	At_RPP2b (TNL)	Arabidopsis thaliana (vTAIR10)	At04	10,633,629	630	0	92.50%	1007	AT4G19510	TNL	At_RPP2b gene		17
130	At_RPP2b (TNL)	Arabidopsis thaliana (vTAIR10)	At04	10,636,012	346	0	100%	632	AT4G19510	TNL	At_RPP2b gene		17
131	At_RPP2b (TNL)	Arabidopsis thaliana (vTAIR10)	At04	10,637,149	230	0	99.50%	500	AT4G19510	TNL	At_RPP2b gene		17
132	At_RPP2b (TNL)	Arabidopsis thaliana (vTAIR10)	At04	10,637,150	628	0	94.10%	985	AT4G19510	TNL	At_RPP2b gene		17
133	At_WRR9 (NL)	Arabidopsis thaliana (vTAIR10)	At04	12,892,778	158	1.00E-59	81.60%	229	AT4G25120		At_WRR9 paralog (segmented)	lost	
134	At_RPS2 (NL)	Arabidopsis thaliana (vTAIR10)	At04	13,224,338	1178	0	100%	2209	AT4G26090	NL	At_RPS2 gene		
135	At_ADR1 (NL)	Arabidopsis thaliana (vTAIR10)	At04	16,051,992	276	0	70.20%	392	AT4G33300	NL	At_ADR1 paralog (segmented)	same	18
136	At_BAK1 (LRR-RLK)	Arabidopsis thaliana (vTAIR10)	At04	16,087,454	266	0	87.90%	467	AT4G33430	LRR-RLK	At_BAK1 gene		18
137	At_ADR1 (NL)	Arabidopsis thaliana (vTAIR10)	At05	1,362,832	277	0	72.90%	399	AT5G04720	NL	At_ADR1 paralog (segmented)	same	
138	At_PBS1 (STK)	Arabidopsis thaliana (vTAIR10)	At05	4,179,224	152	4.00E-158	100%	291	AT5G13160		At_PBS1 gene		
139	At_RLP32 (LRR-RLP)	Arabidopsis thaliana (vTAIR10)	At05	9,524,816	760	0	70.50%	794	AT5G27060	LRR-RLP	At_RLP32 paralog (segmented)	same	
140	At_RPP8 (CNL)	Arabidopsis thaliana (vTAIR10)	At05	13,667,809	294	0	85.30%	424	AT5G35450	CNL	At_RPP8 paralog (tandem)	same	
141	At_RPP8 (CNL)	Arabidopsis thaliana (vTAIR10)	At05	13,669,015	556	0	78.40%	817	AT5G35450	CNL	At_RPP8 paralog (tandem)	same	
142	At_RPP1 (TNL)	Arabidopsis thaliana (vTAIR10)	At05	15,326,115	149	1.00E-67	75.80%	253	AT5G38344	TX	At_RPP1 paralog (segmented)	different	
143	At_WRR12 (TNL)	Arabidopsis thaliana (vTAIR10)	At05	16,043,973	156	0	70.50%	210	AT5G40100	TNL	At_WRR12 paralog (segmented)	same	
144	At_RPP8 (CNL)	Arabidopsis thaliana (vTAIR10)	At05	17,464,833	567	0	100%	1100	AT5G43470	CNL	At_RPP8 gene		
145	At_RPP8 (CNL)	Arabidopsis thaliana (vTAIR10)	At05	17,466,658	384	4.00E-176	88.80%	573	AT5G43470	CNL	At_RPP8 gene		
146	Bra_Crr1a (TNL)	Arabidopsis thaliana (vTAIR10)	At05	17,934,083	149	0	70.40%	230	AT5G44510	TNL	Bra_Crr1a ortholog	same	
147	At_RRS1 (TNL)	Arabidopsis thaliana (vTAIR10)	At05	18,181,198	374	0	74.50%	558	AT5G45050	NL	At_RRS1 paralog (tandem)	different	19
148	At_RPS4 (TNL)	Arabidopsis thaliana (vTAIR10)	At05	18,185,357	184	0	70.10%	278	AT5G45060	TNL	At_RPS4 paralog (tandem)	same	19
149	At_RPS4 (TNL)	Arabidopsis thaliana (vTAIR10)	At05	18,322,711	296	0	100%	621	AT5G45250	TNL	At_RPS4 gene		19
150	At_RPS4 (TNL)	Arabidopsis thaliana (vTAIR10)	At05	18,323,712	295	0	100%	582	AT5G45250	TNL	At_RPS4 gene		19
151	At_RPS4 (TNL)	Arabidopsis thaliana (vTAIR10)	At05	18,326,175	652	0	92.40%	1195	AT5G45250	TNL	At_RPS4 gene		19
152	At_RRS1 (TNL)	Arabidopsis thaliana (vTAIR10)	At05	18,326,716	387	0	96.60%	783	AT5G45260	TNL	At_RRS1 gene		19
153	At_RRS1 (TNL)	Arabidopsis thaliana (vTAIR10)	At05	18,327,939	442	0	92.30%	789	AT5G45260	TNL	At_RRS1 gene		19
154	At_RRS1 (TNL)	Arabidopsis thaliana (vTAIR10)	At05	18,329,450	280	2.00E-178	100%	593	AT5G45260	TNL	At_RRS1 gene		19
155	At_RAC1 (TNL)	Arabidopsis thaliana (vTAIR10)	At05	18,762,810	371	0	71.10%	462	AT5G46260	TNL	At_RAC1 paralog (segmented)	same	20
156	At_WRR8 (TNL)	Arabidopsis thaliana (vTAIR10)	At05	18,762,810	373	0	82.80%	632	AT5G46260	TNL	At_WRR8 paralog (tandem)	same	20
157	At_RAC1 (TNL)	Arabidopsis thaliana (vTAIR10)	At05	18,763,334	148	0	92.50%	283	AT5G46260	TNL	At_RAC1 paralog (segmented)	same	20
158	At_WRR8 (TNL)	Arabidopsis thaliana (vTAIR10)	At05	18,763,337	148	0	92.50%	298	AT5G46260	TNL	At_WRR8 paralog (tandem)	same	20
159	At_WRR8 (TNL)	Arabidopsis thaliana (vTAIR10)	At05	18,766,041	620	0	94.80%	1194	AT5G46270	TNL	At_WRR8 gene		20
160	At_RAC1 (TNL)	Arabidopsis thaliana (vTAIR10)	At05	18,768,296	370	0	73.70%	483	AT5G46270	TNL	At_RAC1 paralog (segmented)	same	20
161	At_WRR8 (TNL)	Arabidopsis thaliana (vTAIR10)	At05	18,768,296	371	0	100%	767	AT5G46270	TNL	At_WRR8 gene		20
162	At_WRR8 (TNL)	Arabidopsis thaliana (vTAIR10)	At05	18,768,874	164	0	98.70%	343	AT5G46270	TNL	At_WRR8 gene		20
163	At_FLS2 (LRR-RLK)	Arabidopsis thaliana (vTAIR10)	At05	18,791,856	1183	0	97.60%	2112	AT5G46330	LRR-RLK	At_FLS2 gene		20
164	At_WRR8 (TNL)	Arabidopsis thaliana (vTAIR10)	At05	18,850,869	626	0	71.20%	855	AT5G46490	TN	At_WRR8 paralog (tandem)	different	20
165	At_RAC1 (TNL)	Arabidopsis thaliana (vTAIR10)	At05	18,850,872	628	0	77.70%	895	AT5G46490	TN	At_RAC1 paralog (segmented)	different	20
166	At_RAC1 (TNL)	Arabidopsis thaliana (vTAIR10)	At05	18,853,325	155	6.00E-72	74.80%	183	AT5G46490	TN	At_RAC1 paralog (segmented)	different	20
167	At_WRR8 (TNL)	Arabidopsis thaliana (vTAIR10)	At05	18,867,813	164	0	90.20%	294	AT5G46520	TNL	At_WRR8 paralog (tandem)	same	20
168	At_RAC1 (TNL)	Arabidopsis thaliana (vTAIR10)	At05	18,867,864	148	0	90.50%	277	AT5G46520	TNL	At_RAC1 paralog (segmented)	same	20
169	At_RPP8 (CNL)	Arabidopsis thaliana (vTAIR10)	At05	19,717,406	293	2.00E-155	94.10%	455	AT5G48620	CNL	At_RPP8 paralog (tandem)	same	
170	At_RPP8 (CNL)	Arabidopsis thaliana (vTAIR10)	At05	19,719,229	566	0	87.80%	944	AT5G48620	CNL	At_RPP8 paralog (tandem)	same	
171	At_NRG1a (RNL)	Arabidopsis thaliana (vTAIR10)	At05	26,715,767	278	1.00E-156	100%	513	AT5G66900	NL	At_NRG1a gene		21
172	At_NRG1b (RNL)	Arabidopsis thaliana (vTAIR10)	At05	26,715,851	306	5.00E-140	81.30%	465	AT5G66900	NL	At_NRG1b paralog (tandem)	different	21
173	At_NRG1a (RNL)	Arabidopsis thaliana (vTAIR10)	At05	26,717,757	381	0	91.60%	632	AT5G66900	NL	At_NRG1a gene		21
174	At_NRG1a (RNL)	Arabidopsis thaliana (vTAIR10)	At05	26,719,174	278	0	87.70%	453	AT5G66910	NL	At_NRG1a paralog (tandem)	different	21
175	At_NRG1b (RNL)	Arabidopsis thaliana (vTAIR10)	At05	26,719,174	278	0	100%	541	AT5G66910	NL	At_NRG1b gene		21
176	At_NRG1b (RNL)	Arabidopsis thaliana (vTAIR10)	At05	26,721,133	219	4.00E-105	99%	362	AT5G66910	NL	At_NRG1b gene		21
177	Bna_Rlm9/4/7 (Other-RLK)	Brassica carinata (v1)	B01	1,893,923	359	4.00E-148	77.90%	490	BcaB02g06592	Other-RLK	Bna_Rlm9/4/7 ortholog	same	
178	Bna_Rlm9/4/7 (Other-RLK)	Brassica carinata (v1)	B01	6,693,142	414	0	91.50%	667	BcaB02g07231	Other-RLK	Bna_Rlm9/4/7 ortholog	same	1
179	Bna_Rlm9/4/7 (Other-RLK)	Brassica carinata (v1)	B01	6,698,248	344	1.00E-136	72.30%	457	BcaB02g07231	Other-RLK	Bna_Rlm9/4/7 ortholog	same	1
180	Bna_Rlm9/4/7 (Other-RLK)	Brassica carinata (v1)	B01	6,702,189	414	0	89.10%	635	BcaB02g07231	Other-RLK	Bna_Rlm9/4/7 ortholog	same	1
181	Bna_Rlm9/4/7 (Other-RLK)	Brassica carinata (v1)	B01	6,717,481	419	0	92.80%	691	BcaB02g07233	Other-RLK	Bna_Rlm9/4/7 ortholog	same	1
182	Bna_Rlm9/4/7 (Other-RLK)	Brassica carinata (v1)	B01	6,718,855	343	2.00E-162	80.10%	532	BcaB02g07233	Other-RLK	Bna_Rlm9/4/7 ortholog	same	1
183	At_RPP13 (CNL)	Brassica carinata (v1)	B01	8,859,618	502	0	76.60%	627	BcaB01g00987	NBS	At_RPP13 ortholog	different	
184	Bju_WRR1 (CNL)	Brassica carinata (v1)	B01	20,068,675	281	2.00E-117	74%	374	BcaB02g09357		Bju_WRR1 ortholog	lost	
185	At_SOBIR1 (LRR-RLK)	Brassica carinata (v1)	B01	58,439,633	641	0	82.60%	1015	BcaB01g04010	LRR-RLK	At_SOBIR1 ortholog	same	
186	At_PBS1 (STK)	Brassica carinata (v1)	B01	66,645,031	207	2.00E-86	72.40%	298	BcaB01g05372		At_PBS1 ortholog	lost	
187	At_PBS1 (STK)	Brassica carinata (v1)	B01	66,645,805	160	1.00E-65	77.50%	237	BcaB01g05372		At_PBS1 ortholog	lost	
188	Bna_Rlm9/4/7 (Other-RLK)	Brassica carinata (v1)	B02	6,736,225	247	2.00E-110	79.30%	380	BcaB02g07234		Bna_Rlm9/4/7 ortholog	lost	
189	At_NRG1a (RNL)	Brassica carinata (v1)	B02	24,028,938	288	5.00E-101	70.40%	352	BcaB02g09973	CNL	At_NRG1a ortholog	different	
190	At_NRG1b (RNL)	Brassica carinata (v1)	B02	24,028,956	279	8.00E-106	71.60%	367	BcaB02g09973	CNL	At_NRG1b ortholog	different	
191	Bna_MAPk (Other-RLK)	Brassica carinata (v1)	B02	33,299,083	176	3.00E-85	81.20%	287	BcaB02g10915		Bna_MAPk ortholog	lost	
192	At_RPP2b (TNL)	Brassica carinata (v1)	B03	376,327	208	5.00E-135	71.10%	315	BcaB03g11622	TNL	At_RPP2b ortholog	same	2
193	At_RPP2b (TNL)	Brassica carinata (v1)	B03	445,736	192	0.00E+00	82.20%	315	BcaB03g11627		At_RPP2b ortholog	lost	2
194	At_RPP2a (TNL)	Brassica carinata (v1)	B03	445,737	192	0	71.80%	233	BcaB03g11627		At_RPP2a ortholog	lost	2
195	Bra_Crr1a (TNL)	Brassica carinata (v1)	B03	1,011,629	510	0	80.10%	776	BcaB03g11720	TNL	Bra_Crr1a ortholog	same	
196	At_BAK1 (LRR-RLK)	Brassica carinata (v1)	B03	7,119,572	187	6.00E-71	82.30%	258	BcaB03g12797	LRR-RLK	At_BAK1 ortholog	same	
197	Bna_Rlm9/4/7 (Other-RLK)	Brassica carinata (v1)	B03	7,682,549	167	1.00E-125	74.20%	243	BcaB03g12914	Other-RLK	Bna_Rlm9/4/7 ortholog	same	
198	At_RFO2 (LRR-RLP)	Brassica carinata (v1)	B03	8,107,855	603	0	79.90%	873	BcaB03g13011	LRR-RLP	At_RFO2 ortholog	same	
199	At_ADR1 (NL)	Brassica carinata (v1)	B03	8,595,126	274	8.00E-131	70.80%	374	BcaB03g13136	CNL	At_ADR1 ortholog	different	
200	Bna_Rlm9/4/7 (Other-RLK)	Brassica carinata (v1)	B03	10,162,899	282	2.00E-142	82.20%	400	BcaB03g13465	Other-RLK	Bna_Rlm9/4/7 ortholog	same	3
201	Bna_Rlm9/4/7 (Other-RLK)	Brassica carinata (v1)	B03	10,189,876	423	2.00E-160	71.80%	526	BcaB03g13465	Other-RLK	Bna_Rlm9/4/7 ortholog	same	3
202	Bna_Rlm9/4/7 (Other-RLK)	Brassica carinata (v1)	B03	10,193,788	367	5.00E-143	73.20%	476	BcaB03g13466	Other-RLK	Bna_Rlm9/4/7 ortholog	same	3
203	Bna_Rlm9/4/7 (Other-RLK)	Brassica carinata (v1)	B03	10,536,730	255	2.00E-112	72.10%	306	BcaB03g13525		Bna_Rlm9/4/7 ortholog	lost	
204	Bna_Rlm9/4/7 (Other-RLK)	Brassica carinata (v1)	B03	18,093,173	345	7.00E-133	75.30%	446	BcaB03g14448		Bna_Rlm9/4/7 ortholog	lost	
205	Bna_Rlm9/4/7 (Other-RLK)	Brassica carinata (v1)	B03	18,103,516	357	1.00E-137	71.10%	460	BcaB03g14448		Bna_Rlm9/4/7 ortholog	lost	
206	Bna_Rlm9/4/7 (Other-RLK)	Brassica carinata (v1)	B03	18,482,738	352	2.00E-128	70.70%	432	BcaB03g14504	Other-RLK	Bna_Rlm9/4/7 ortholog	same	4
207	Bna_Rlm9/4/7 (Other-RLK)	Brassica carinata (v1)	B03	18,490,858	306	4.00E-123	72.20%	417	BcaB03g14506	Other-RLK	Bna_Rlm9/4/7 ortholog	same	4
208	Bna_Rlm9/4/7 (Other-RLK)	Brassica carinata (v1)	B03	18,505,349	341	4.00E-128	70.30%	432	BcaB03g14506	Other-RLK	Bna_Rlm9/4/7 ortholog	same	4
209	At_RFO3 (Other-RLK)	Brassica carinata (v1)	B03	19,504,787	192	2.00E-94	75.50%	284	BcaB03g14632	Other-RLK	At_RFO3 ortholog	same	
210	At_RFO3 (Other-RLK)	Brassica carinata (v1)	B03	19,505,406	190	5.00E-70	73.60%	261	BcaB03g14632	Other-RLK	At_RFO3 ortholog	same	
211	At_RFO3 (Other-RLK)	Brassica carinata (v1)	B03	19,508,165	190	1.00E-69	73.60%	260	BcaB03g14632	Other-RLK	At_RFO3 ortholog	same	
212	At_BAK1 (LRR-RLK)	Brassica carinata (v1)	B03	27,616,827	270	4.00E-166	88.50%	491	BcaB03g15620	LRR-RLK	At_BAK1 ortholog	same	
213	At_NDR1 (TM)	Brassica carinata (v1)	B04	42,091,686	219	5.00E-104	79.40%	338	BcaB04g19117		At_NDR1 ortholog	lost	
214	At_NRG1a (RNL)	Brassica carinata (v1)	B04	49,152,621	230	2.00E-74	71.30%	273	BcaB04g20089	CNL	At_NRG1a ortholog	different	5
215	At_NRG1a (RNL)	Brassica carinata (v1)	B04	49,154,535	378	3.00E-151	74.30%	500	BcaB04g20089	CNL	At_NRG1a ortholog	different	5
216	At_NRG1b (RNL)	Brassica carinata (v1)	B04	49,154,535	378	2.00E-151	73%	501	BcaB04g20089	CNL	At_NRG1b ortholog	different	5
217	At_NRG1a (RNL)	Brassica carinata (v1)	B04	49,168,452	153	1.00E-97	77.10%	220	BcaB04g20090		At_NRG1a ortholog	lost	5
218	At_NRG1a (RNL)	Brassica carinata (v1)	B04	49,172,835	300	3.00E-106	73%	368	BcaB04g20091	NL	At_NRG1a ortholog	different	5
219	At_NRG1b (RNL)	Brassica carinata (v1)	B04	49,172,859	294	1.00E-111	74.40%	384	BcaB04g20091	NL	At_NRG1b ortholog	different	5
220	At_NRG1a (RNL)	Brassica carinata (v1)	B04	50,237,747	279	0	74.10%	389	BcaB04g20245	CNL	At_NRG1a ortholog	different	
221	At_NRG1b (RNL)	Brassica carinata (v1)	B04	50,237,753	281	0	71.50%	391	BcaB04g20245	CNL	At_NRG1b ortholog	different	
222	At_NDR1 (TM)	Brassica carinata (v1)	B04	53,219,483	218	5.00E-102	76.10%	332	BcaB04g20643		At_NDR1 ortholog	lost	
223	At_RFO3 (Other-RLK)	Brassica carinata (v1)	B04	54,715,786	186	3.00E-108	75.80%	254	BcaB04g20879	Other-RLK	At_RFO3 ortholog	same	
224	At_RFO3 (Other-RLK)	Brassica carinata (v1)	B04	54,718,413	162	4.00E-57	76.50%	220	BcaB04g20879	Other-RLK	At_RFO3 ortholog	same	
225	At_RPM1 (NL)	Brassica carinata (v1)	B04	57,094,684	581	0	82.40%	952	BcaB04g21306		At_RPM1 ortholog	lost	
226	At_RPM1 (NL)	Brassica carinata (v1)	B04	57,097,840	320	3.00E-173	80%	467	BcaB04g21306		At_RPM1 ortholog	lost	
227	At_PBS1 (STK)	Brassica carinata (v1)	B05	2,806,022	221	2.00E-129	74.20%	344	BcaB05g21868		At_PBS1 ortholog	lost	
228	At_RFO3 (Other-RLK)	Brassica carinata (v1)	B06	2,594,342	386	2.00E-170	73%	531	BcaB06g25699	Other-RLK	At_RFO3 ortholog	same	

229	*Bna_MAPk (Other-RLK)*	Brassica carinata (v1)	B06	3,902,200	262	1.00E-125	76.30%	360	BcaB06g25874		Bna_MAPk ortholog	lost	
230	*At_NDR1 (TM)*	Brassica carinata (v1)	B06	5,858,689	217	4.00E-90	73.70%	298	BcaB06g26124		At_NDR1 ortholog	lost	
231	*At_RLP32 (LRR-RLP)*	Brassica carinata (v1)	B06	41,364,636	439	3.00E-148	70.30%	493	BcaB06g27884	LRR-RLP	At_RLP32 ortholog	same	
232	*At_SOBIR1 (LRR-RLK)*	Brassica carinata (v1)	B06	47,804,283	643	0	78.30%	911	BcaB06g28572	LRR-RLK	At_SOBIR1 ortholog	same	
233	*At_RLP32 (LRR-RLP)*	Brassica carinata (v1)	B06	55,417,134	402	7.00E-149	75.30%	495	BcaB06g29641		At_RLP32 ortholog	lost	
234	*At_FLS2 (LRR-RLK)*	Brassica carinata (v1)	B07	7,207,607	1031	0	80.60%	1533	BcaB07g30842	LRR-RLK	At_FLS2 ortholog	same	
235	*At_RPS4 (TNL)*	Brassica carinata (v1)	B07	7,588,261	424	0	78%	649	BcaB07g30900	TNL	At_RPS4 ortholog	same	
236	*At_RLM1b (TNL)*	Brassica carinata (v1)	B07	11,370,151	372	0	73.90%	561	BcaB07g31416		At_RLM1b ortholog	lost	
237	*At_RLM1b (TNL)*	Brassica carinata (v1)	B07	11,380,563	376	0	70.40%	532	BcaB07g31417		At_RLM1b ortholog	lost	
238	*At_RLM1b (TNL)*	Brassica carinata (v1)	B07	12,300,969	369	1.00E-162	72%	546	BcaB07g31538	TNL	At_RLM1b ortholog	same	
239	*Bna_Rlm9/4/7 (Other-RLK)*	Brassica carinata (v1)	B07	15,444,627	361	1.00E-128	72.20%	434	BcaB07g31950		Bna_Rlm9/4/7 ortholog	lost	
240	*Bna_Rlm9/4/7 (Other-RLK)*	Brassica carinata (v1)	B07	15,477,760	323	7.00E-119	72.70%	405	BcaB07g31953		Bna_Rlm9/4/7 ortholog	lost	
241	*Bna_Rlm9/4/7 (Other-RLK)*	Brassica carinata (v1)	B07	15,481,435	362	4.00E-130	72.30%	438	BcaB07g31954		Bna_Rlm9/4/7 ortholog	lost	
242	*At_NRG1b (RNL)*	Brassica carinata (v1)	B08	3,410,045	268	3.00E-100	70.50%	350	BcaB08g34626	CNL	At_NRG1b ortholog	different	
243	*At_NRG1a (RNL)*	Brassica carinata (v1)	B08	3,410,045	263	4.00E-95	70.30%	335	BcaB08g34626	CNL	At_NRG1a ortholog	different	
244	*Bna_MAPk (Other-RLK)*	Brassica carinata (v1)	B08	40,041,512	212	7.00E-162	75%	295	BcaB08g36628		Bna_MAPk ortholog	lost	
245	*Bra_Crr1a (TNL)*	Brassica carinata (v1)	C01	7,116,127	433	6.00E-179	70.60%	556	BcaC01g00655		Bra_Crr1a ortholog	lost	
246	*Bra_Crr1a (TNL)*	Brassica carinata (v1)	C01	8,094,446	358	0	80.70%	507	BcaC01g00753		Bra_Crr1a ortholog	lost	6
247	*Bra_Crr1a (TNL)*	Brassica carinata (v1)	C01	8,096,716	372	0	91.60%	672	BcaC01g00753		Bra_Crr1a ortholog	lost	6
248	*Bra_Crr1a (TNL)*	Brassica carinata (v1)	C01	8,114,956	372	0	91.60%	674	BcaC01g00754	TNL	Bra_Crr1a ortholog	same	6
249	*Bra_Crr1a (TNL)*	Brassica carinata (v1)	C01	8,132,310	372	0	83%	594	BcaC01g00754	TNL	Bra_Crr1a ortholog	same	6
250	*Bra_Crr1a (TNL)*	Brassica carinata (v1)	C01	8,160,671	440	0	71.30%	572	BcaC01g00755		Bra_Crr1a ortholog	lost	6
251	*At_BAK1 (LRR-RLK)*	Brassica carinata (v1)	C01	11,043,308	181	8.00E-73	85%	263	BcaC01g00989	LRR-RLK	At_BAK1 ortholog	same	
252	*At_BAK1 (LRR-RLK)*	Brassica carinata (v1)	C01	11,044,385	268	4.00E-129	81.70%	428	BcaC01g00989	LRR-RLK	At_BAK1 ortholog	same	
253	*At_BAK1 (LRR-RLK)*	Brassica carinata (v1)	C01	39,533,830	264	1.00E-105	70.40%	360	BcaC01g03115	LRR-RLK	At_BAK1 ortholog	same	
254	*At_RFO3 (Other-RLK)*	Brassica carinata (v1)	C01	42,803,774	295	1.00E-168	72.80%	399	BcaC01g03391	Other-RLK	At_RFO3 ortholog	same	
255	*At_NDR1 (TM)*	Brassica carinata (v1)	C01	44,399,440	220	5.00E-100	78.10%	327	BcaC01g03546		At_NDR1 ortholog	lost	
256	*Bna_Rlm9/4/7 (Other-RLK)*	Brassica carinata (v1)	C01	52,457,037	414	3.00E-165	75.30%	540	BcaC01g04499	Other-RLK	Bna_Rlm9/4/7 ortholog	same	
257	*At_SOBIR1 (LRR-RLK)*	Brassica carinata (v1)	C01	64,297,247	357	2.00E-128	75.30%	427	BcaC01g05928	LRR-RLP	At_SOBIR1 ortholog	different	
258	*At_RPP13 (CNL)*	Brassica carinata (v1)	C02	2,579,544	204	5.00E-64	80.80%	242	BcaC02g07331		At_RPP13 ortholog	lost	
259	*At_SOBIR1 (LRR-RLK)*	Brassica carinata (v1)	C02	7,265,115	643	0	80.20%	939	BcaC02g07960	LRR-RLK	At_SOBIR1 ortholog	same	
260	*Bju_WRR1 (CNL)*	Brassica carinata (v1)	C02	56,010,083	573	0	81.80%	882	BcaC02g11752	CNL	Bju_WRR1 ortholog	same	
261	*Bju_WRR1 (CNL)*	Brassica carinata (v1)	C02	56,011,952	310	8.00E-167	81.60%	491	BcaC02g11752	CNL	Bju_WRR1 ortholog	same	
262	*At_FLS2 (LRR-RLK)*	Brassica carinata (v1)	C03	49,547,168	191	3.00E-90	86.30%	328	BcaC03g16847	LRR-RLK	At_FLS2 ortholog	same	7
263	*At_RAC1 (TNL)*	Brassica carinata (v1)	C03	49,583,827	238	2.00E-125	71%	289	BcaC03g16850	NBS	At_RAC1 ortholog	different	7
264	*At_WRR8 (TNL)*	Brassica carinata (v1)	C03	49,583,827	239	2.00E-100	71.90%	353	BcaC03g16850	NBS	At_WRR8 ortholog	different	7
265	*At_NRG1a (RNL)*	Brassica carinata (v1)	C03	59,934,116	167	1.00E-63	74.20%	240	BcaC03g17818	NL	At_NRG1a ortholog	different	
266	*At_NRG1b (RNL)*	Brassica carinata (v1)	C03	59,934,122	169	1.00E-70	75.70%	262	BcaC03g17818	NL	At_NRG1b ortholog	different	
267	*At_PBS1 (STK)*	Brassica carinata (v1)	C04	5,025,627	156	1.00E-152	94.80%	286	BcaC04g19142		At_PBS1 ortholog	lost	
268	*At_FLS2 (LRR-RLK)*	Brassica carinata (v1)	C04	40,446,731	1029	0	80.90%	1534	BcaC04g21710	LRR-RLK	At_FLS2 ortholog	same	
269	*At_RPS4 (TNL)*	Brassica carinata (v1)	C04	41,520,535	269	3.00E-110	76.90%	393	BcaC04g21788		At_RPS4 ortholog	lost	8
270	*At_RPS4 (TNL)*	Brassica carinata (v1)	C04	41,528,052	563	0	72.20%	797	BcaC04g21789	TN	At_RPS4 ortholog	different	8
271	*At_RRS1 (TNL)*	Brassica carinata (v1)	C04	41,541,737	322	0	71.40%	439	BcaC04g21790	NL	At_RRS1 ortholog	different	8
272	*At_RPP39 (CNL)*	Brassica carinata (v1)	C04	48,278,371	358	0	70.10%	464	BcaC04g22316	CNL	At_RPP39 ortholog	same	
273	*At_RLM1b (TNL)*	Brassica carinata (v1)	C04	48,590,767	373	7.00E-153	70.20%	517	BcaC04g22342	TNL	At_RLM1b ortholog	same	
274	*At_RLM1b (TNL)*	Brassica carinata (v1)	C04	50,895,268	313	0	71.80%	467	BcaC04g22512	TNL	At_RLM1b ortholog	same	
275	*At_RLM1b (TNL)*	Brassica carinata (v1)	C04	52,324,787	372	0	73.30%	556	BcaC04g22671	TNL	At_RLM1b ortholog	same	
276	*At_NRG1a (RNL)*	Brassica carinata (v1)	C04	57,253,593	284	6.00E-125	80.90%	423	BcaC04g23168	CNL	At_NRG1a ortholog	different	9
277	*At_NRG1b (RNL)*	Brassica carinata (v1)	C04	57,253,593	284	6.00E-127	79.90%	429	BcaC04g23168	CNL	At_NRG1b ortholog	different	9
278	*At_NRG1a (RNL)*	Brassica carinata (v1)	C04	57,254,880	257	3.00E-147	84.80%	438	BcaC04g23168	CNL	At_NRG1a ortholog	different	9
279	*At_NRG1a (RNL)*	Brassica carinata (v1)	C04	57,255,796	217	2.00E-72	72.30%	267	BcaC04g23168	CNL	At_NRG1a ortholog	different	9
280	*At_NRG1a (RNL)*	Brassica carinata (v1)	C04	57,283,901	279	6.00E-141	82.40%	426	BcaC04g23170	NL	At_NRG1a ortholog	different	9
281	*At_NRG1b (RNL)*	Brassica carinata (v1)	C04	57,283,901	279	4.00E-141	81.30%	432	BcaC04g23170	NL	At_NRG1b ortholog	different	9
282	*At_NRG1a (RNL)*	Brassica carinata (v1)	C04	57,285,241	284	9.00E-115	72.50%	393	BcaC04g23170	NL	At_NRG1a ortholog	different	9
283	*Bju_WRR1 (CNL)*	Brassica carinata (v1)	C04	65,431,723	242	4.00E-55	80.90%	215	BcaC04g24216		Bju_WRR1 ortholog	lost	
284	*Bna_Rlm9/4/7 (Other-RLK)*	Brassica carinata (v1)	C05	9,209,235	258	5.00E-118	77.90%	352	BcaC05g25598	Other-RLK	Bna_Rlm9/4/7 ortholog	same	
285	*Bna_MAPk (Other-RLK)*	Brassica carinata (v1)	C05	10,111,460	155	1.00E-160	81.90%	232	BcaC05g25706		Bna_MAPk ortholog	lost	
286	*Bna_Rlm9/4/7 (Other-RLK)*	Brassica carinata (v1)	C05	11,114,094	203	1.00E-70	74.30%	261	BcaC05g25827		Bna_Rlm9/4/7 ortholog	lost	
287	*Bna_MAPk (Other-RLK)*	Brassica carinata (v1)	C05	50,492,391	255	7.00E-127	78%	364	BcaC05g28713		Bna_MAPk ortholog	lost	
288	*At_RFO3 (Other-RLK)*	Brassica carinata (v1)	C05	52,887,436	159	6.00E-107	77.90%	247	BcaC05g28918	Other-RLK	At_RFO3 ortholog	same	
289	*At_RPP2b (TNL)*	Brassica carinata (v1)	C06	382,946	217	2.00E-121	78.80%	366	BcaC06g30633		At_RPP2b ortholog	lost	
290	*Bju_WRR1 (CNL)*	Brassica carinata (v1)	C06	950,233	320	4.00E-140	70.90%	448	BcaC06g30724	CNL	Bju_WRR1 ortholog	same	10
291	*Bju_WRR1 (CNL)*	Brassica carinata (v1)	C06	1,048,120	389	1.00E-157	73.50%	547	BcaC06g30745	CNL	Bju_WRR1 ortholog	same	10
292	*At_ADR1 (NL)*	Brassica carinata (v1)	C06	9,963,235	277	3.00E-115	71.10%	394	BcaC06g32180	NL	At_ADR1 ortholog	same	
293	*Bol_FocBo1 (TNL)*	Brassica carinata (v1)	C06	17,906,954	402	0	92.20%	760	BcaC06g33518	TNL	Bol_FocBo1 ortholog	same	
294	*Bol_FocBo1 (TNL)*	Brassica carinata (v1)	C06	17,907,685	166	1.00E-89	97.50%	328	BcaC06g33518	TNL	Bol_FocBo1 ortholog	same	
295	*Bol_FocBo1 (TNL)*	Brassica carinata (v1)	C06	17,921,371	331	0	93.30%	540	BcaC06g33518	TNL	Bol_FocBo1 ortholog	same	
296	*Bol_FocBo1 (TNL)*	Brassica carinata (v1)	C06	17,921,894	149	0	99.30%	277	BcaC06g33518	TNL	Bol_FocBo1 ortholog	same	
297	*At_NRG1a (RNL)*	Brassica carinata (v1)	C06	20,201,771	265	1.00E-101	71.60%	355	BcaC06g33794	CNL	At_NRG1a ortholog	different	
298	*At_NRG1b (RNL)*	Brassica carinata (v1)	C06	20,201,795	273	1.00E-105	70.30%	367	BcaC06g33794	CNL	At_NRG1b ortholog	different	
299	*At_BAK1 (LRR-RLK)*	Brassica carinata (v1)	C06	48,441,923	157	3.00E-128	71.30%	229	BcaC06g35998	LRR-RLK	At_BAK1 ortholog	same	
300	*Bna_Rlm9/4/7 (Other-RLK)*	Brassica carinata (v1)	C07	739,723	308	3.00E-122	75%	415	BcaC07g36871	Other-RLK	Bna_Rlm9/4/7 ortholog	same	
301	*Bol_FocBo1 (TNL)*	Brassica carinata (v1)	C07	20,913,883	253	2.00E-180	71.90%	291	BcaC07g38028	TNL	Bol_FocBo1 ortholog	same	
302	*Bna_Rlm9/4/7 (Other-RLK)*	Brassica carinata (v1)	C07	46,274,101	255	1.00E-107	74.90%	348	BcaC07g41258		Bna_Rlm9/4/7 ortholog	lost	
303	*At_WRR12 (TNL)*	Brassica carinata (v1)	C07	46,975,990	442	2.00E-141	70.80%	493	BcaC07g41370	Other-NLR	At_WRR12 ortholog	different	11
304	*At_WRR12 (TNL)*	Brassica carinata (v1)	C07	46,978,122	149	3.00E-69	86.50%	265	BcaC07g41371	TNL	At_WRR12 ortholog	same	11
305	*At_WRR12 (TNL)*	Brassica carinata (v1)	C07	46,980,140	508	0	78.50%	803	BcaC07g41371	TNL	At_WRR12 ortholog	same	11
306	*At_WRR12 (TNL)*	Brassica carinata (v1)	C07	46,981,943	304	0	81.90%	463	BcaC07g41371	TNL	At_WRR12 ortholog	same	11
307	*At_RLP1 (LRR-RLP)*	Brassica carinata (v1)	C07	51,237,013	260	8.00E-102	73.80%	362	BcaC07g42003	LRR-RLP	At_RLP1 ortholog	same	12
308	*At_RLP1 (LRR-RLP)*	Brassica carinata (v1)	C07	51,258,186	216	0	74.50%	317	BcaC07g42005	LRR-RLP	At_RLP1 ortholog	same	12
309	*At_RLP1 (LRR-RLP)*	Brassica carinata (v1)	C07	51,259,743	271	0	70.10%	329	BcaC07g42005	LRR-RLP	At_RLP1 ortholog	same	12
310	*At_RLP1 (LRR-RLP)*	Brassica carinata (v1)	C07	51,266,894	740	0	81.40%	1093	BcaC07g42006	LRR-RLP	At_RLP1 ortholog	same	12
311	*At_BAK1 (LRR-RLK)*	Brassica carinata (v1)	C08	45,533,019	155	1.00E-140	80.60%	253	BcaC08g46614	LRR-RLK	At_BAK1 ortholog	same	
312	*At_RFO1 (Other-RLK)*	Brassica carinata (v1)	C08	52,650,597	293	0	71.60%	337	BcaC08g47526	Other-RLK	At_RFO1 ortholog	same	13
313	*Bna_Rlm9/4/7 (Other-RLK)*	Brassica carinata (v1)	C08	52,653,788	414	0	80.40%	590	BcaC08g47527	Other-RLK	Bna_Rlm9/4/7 ortholog	same	13
314	*At_RPM1 (NL)*	Brassica carinata (v1)	C09	2,431,075	724	0	81.60%	1184	BcaC09g47934	NL	At_RPM1 ortholog	same	
315	*At_RFO3 (Other-RLK)*	Brassica carinata (v1)	C09	8,063,849	163	9.00E-58	76%	223	BcaC09g48581		At_RFO3 ortholog	lost	
316	*At_RFO3 (Other-RLK)*	Brassica carinata (v1)	C09	8,064,548	161	7.00E-106	77.60%	253	BcaC09g48581		At_RFO3 ortholog	lost	
317	*At_NDR1 (TM)*	Brassica carinata (v1)	C09	12,408,766	218	5.00E-101	75.20%	330	BcaC09g49003		At_NDR1 ortholog	lost	
318	*At_RPS2 (NL)*	Brassica carinata (v1)	C09	36,334,709	929	0	83.60%	1479	BcaC09g50671	CNL	At_RPS2 ortholog	different	
319	*At_RPP2b (TNL)*	Brassica carinata (v1)	C09	42,743,801	216	2.00E-100	75.40%	361	BcaC09g51400	TNL	At_RPP2b ortholog	same	14
320	*At_RPP2b (TNL)*	Brassica carinata (v1)	C09	42,744,927	352	0	70.10%	405	BcaC09g51400	TNL	At_RPP2b ortholog	same	14
321	*At_RPP2a (TNL)*	Brassica carinata (v1)	C09	42,749,670	466	0	74.20%	686	BcaC09g51401	TNL	At_RPP2a ortholog	same	14
322	*At_BAK1 (LRR-RLK)*	Brassica carinata (v1)	C09	48,437,428	277	0	88%	506	BcaC09g52155	LRR-RLK	At_BAK1 ortholog	same	
323	*Bna_Rlm9/4/7 (Other-RLK)*	Brassica carinata (v1)	JAAMPC010000097.1	75,952	272	0	96.60%	446	BcaNung04173		Bna_Rlm9/4/7 ortholog	lost	
324	*Bna_Rlm9/4/7 (Other-RLK)*	Brassica carinata (v1)	JAAMPC0100000971	51,602	427	0	80.50%	642	BcaNung04172	Other-RLK	Bna_Rlm9/4/7 ortholog	same	
325	*Bna_Rlm9/4/7 (Other-RLK)*	Brassica carinata (v1)	JAAMPC0100000971	52,999	414	0	94.60%	698	BcaNung04172	Other-RLK	Bna_Rlm9/4/7 ortholog	same	
326	*Bna_Rlm9/4/7 (Other-RLK)*	Brassica carinata (v1)	JAAMPC0100000971	60,986	322	7.00E-136	71.70%	454	BcaNung04172	Other-RLK	Bna_Rlm9/4/7 ortholog	same	
327	*Bna_Rlm9/4/7 (Other-RLK)*	Brassica carinata (v1)	JAAMPC0100000971	64,436	414	0	95.10%	699	BcaNung04172	Other-RLK	Bna_Rlm9/4/7 ortholog	same	
328	*Bna_Rlm9/4/7 (Other-RLK)*	Brassica carinata (v1)	JAAMPC0100000971	72,478	313	1.00E-129	70.90%	436	BcaNung04172	Other-RLK	Bna_Rlm9/4/7 ortholog	same	
329	*At_RPP8 (CNL)*	Brassica carinata (v1)	JAAMPC0100002521	1,705	293	4.00E-101	70.90%	356	BcaNung05226	NL	At_RPP8 ortholog	different	
330	*At_RPS2 (NL)*	Brassica carinata (v1)	JAAMPC0100002531	4,646	953	0	82.70%	1473	BcaNung05233	NL	At_RPS2 ortholog	same	
331	*At_RPP39 (CNL)*	Brassica carinata (v1)	JAAMPC010000274.1	1,394	464	0	71.10%	620	BcaNung05332	CN	At_RPP39 ortholog	different	
332	*Bna_MAPk (Other-RLK)*	Brassica carinata (v1)	JAAMPC010000294.1	5,081	162	2.00E-62	78.30%	232	BcaNung05434		Bna_MAPk ortholog	lost	
333	*Bna_MAPk (Other-RLK)*	Brassica carinata (v1)	JAAMPC010001230.1	6,377	165	3.00E-60	75.10%	224	BcaNung00436		Bna_MAPk ortholog	lost	
334	*Bra_cRa/cRb (TNL)*	Brassica carinata (v1)	JAAMPC010001579.1	294,956	220	1.00E-83	77.70%	308	BcaNung00823	Other-NLR	Bra_cRa/cRb ortholog	different	
335	*Bra_cRa/cRb (TNL)*	Brassica carinata (v1)	JAAMPC010001579.1	296,050	374	0	86%	671	BcaNung00823	Other-NLR	Bra_cRa/cRb ortholog	different	
336	*Bra_cRa/cRb (TNL)*	Brassica carinata (v1)	JAAMPC010001579.1	296,050	374	0.00E+00	86.00%	671	BcaNung00823	Other-NLR	Bra_cRa/cRb ortholog	different	
337	*Bra_cRa/cRb (TNL)*	Brassica carinata (v1)	JAAMPC010001579.1	299,113	316	8.00E-125	72.10%	437	BcaNung00823	Other-NLR	Bra_cRa/cRb ortholog	different	
338	*Bra_cRa/cRb (TNL)*	Brassica carinata (v1)	JAAMPC010001579.1	299,113	316	8.00E-125	72.10%	437	BcaNung00823	Other-NLR	Bra_cRa/cRb ortholog	different	
339	*Bra_cRa/cRb (TNL)*	Brassica carinata (v1)	JAAMPC010001579.1	314,973	149	8.00E-76	87.20%	283	BcaNung00823	Other-NLR	Bra_cRa/cRb ortholog	different	
340	*Bra_cRa/cRb (TNL)*	Brassica carinata (v1)	JAAMPC010001579.1	317,054	370	1.00E-175	77.20%	589	BcaNung00823	Other-NLR	Bra_cRa/cRb ortholog	different	
341	*Bra_cRa/cRb (TNL)*	Brassica carinata (v1)	JAAMPC010001579.1	317,054	370	1.00E-175	77.20%	589	BcaNung00823	Other-NLR	Bra_cRa/cRb ortholog	different	
342	*At_PBS1 (STK)*	Brassica carinata (v1)	JAAMPC010001583.1	297,391	311	6.00E-168	86.10%	533	BcaNung01172		At_PBS1 ortholog	lost	
343	*Bra_cRa/cRb (TNL)*	Brassica carinata (v1)	JAAMPC010001591.1	249,576	220	1.00E-81	74.50%	302	BcaNung01716	TNL	Bra_cRa/cRb ortholog	same	
344	*Bra_cRa/cRb (TNL)*	Brassica carinata (v1)	JAAMPC010001591.1	250,658	371	0	70.60%	548	BcaNung01716	TNL	Bra_cRa/cRb ortholog	same	
345	*Bra_cRa/cRb (TNL)*	Brassica carinata (v1)	JAAMPC010001591.1	250,658	371	0.00E+00	70.60%	548	BcaNung01716	TNL	Bra_cRa/cRb ortholog	same	

346	*Bra_cRa/cRb (TNL)*	Brassica carinata (v1)	JAAMPC010001591.1	273,608	149	5.00E-77	87.90%	287	BcaNung01716	TNL	Bra_cRa/cRb ortholog	same	
347	*Bra_cRa/cRb (TNL)*	Brassica carinata (v1)	JAAMPC010001591.1	274,375	374	1.00E-172	76.20%	580	BcaNung01716	TNL	Bra_cRa/cRb ortholog	same	
348	*Bra_cRa/cRb (TNL)*	Brassica carinata (v1)	JAAMPC010001591.1	274,375	374	1.00E-172	76.20%	580	BcaNung01716	TNL	Bra_cRa/cRb ortholog	same	
349	*At_PBS1 (STK)*	Brassica carinata (v1)	JAAMPC010001596.1	30,755	152	9.00E-149	90.10%	272	BcaNung01865		At_PBS1 ortholog	lost	
350	*Bna_MAPk (Other-RLK)*	Brassica juncea (v1.5)	A01	1,053,219	222	4.00E-93	72.90%	322	BjuA040764		Bna_MAPk ortholog	lost	
351	*At_BAK1 (LRR-RLK)*	Brassica juncea (v1.5)	A01	2,253,431	277	0	88.40%	506	BjuA003269		At_BAK1 ortholog	lost	
352	*At_RPS2 (NL)*	Brassica juncea (v1.5)	A01	10,001,942	398	0	80.40%	563	BjuA004393		At_RPS2 ortholog	lost	1
353	*At_RPS2 (NL)*	Brassica juncea (v1.5)	A01	10,003,131	533	0	84.20%	907	BjuA004393		At_RPS2 ortholog	lost	1
354	*At_RPS2 (NL)*	Brassica juncea (v1.5)	A01	10,036,778	398	0	80.40%	563	BjuA004392	CNL	At_RPS2 ortholog	different	1
355	*At_RPS2 (NL)*	Brassica juncea (v1.5)	A01	10,037,967	533	0	84.20%	907	BjuA004392	CNL	At_RPS2 ortholog	different	1
356	*At_NDR1 (TM)*	Brassica juncea (v1.5)	A01	30,391,236	218	5.00E-100	74.70%	327	BjuA000872		At_NDR1 ortholog	lost	
357	*At_RFO3 (Other-RLK)*	Brassica juncea (v1.5)	A01	33,505,086	163	4.00E-57	75.40%	220	BjuA040278		At_RFO3 ortholog	lost	
358	*Bna_MAPk (Other-RLK)*	Brassica juncea (v1.5)	A01	35,140,547	181	1.00E-156	82.30%	303	BjuA006021		Bna_MAPk ortholog	lost	
359	*At_RPM1 (NL)*	Brassica juncea (v1.5)	A01	38,488,712	948	0	80.30%	1451	BjuA006224	NL	At_RPM1 ortholog	same	
360	*At_PBS1 (STK)*	Brassica juncea (v1.5)	A02	493,901	158	9.00E-85	72.70%	232	BjuA040832		At_PBS1 ortholog	lost	
361	*At_PBS1 (STK)*	Brassica juncea (v1.5)	A02	2,506,818	152	4.00E-146	90.10%	265	BjuA041175		At_PBS1 ortholog	lost	
362	*At_FLS2 (LRR-RLK)*	Brassica juncea (v1.5)	A02	24,267,580	358	1.00E-149	73.10%	508	BjuA008024		At_FLS2 ortholog	lost	
363	*At_NRG1a (RNL)*	Brassica juncea (v1.5)	A02	33,331,591	242	4.00E-94	74.70%	332	BjuA015118	NL	At_NRG1a ortholog	different	
364	*At_NRG1b (RNL)*	Brassica juncea (v1.5)	A02	33,331,597	244	8.00E-97	72.90%	340	BjuA015118	NL	At_NRG1b ortholog	different	
365	*At_PBS1 (STK)*	Brassica juncea (v1.5)	A03	2,656,910	160	2.00E-66	76.80%	239	BjuA041684		At_PBS1 ortholog	lost	
366	*Bra_Crr1a (TNL)*	Brassica juncea (v1.5)	A03	6,250,520	372	2.00E-163	70.90%	501	BjuA009474		Bra_Crr1a ortholog	lost	
367	*At_SOBIR1 (LRR-RLK)*	Brassica juncea (v1.5)	A03	9,452,768	647	0	80%	976	BjuA009972	LRR-RLK	At_SOBIR1 ortholog	same	
368	*Bna_Rlm9/4/7 (Other-RLK)*	Brassica juncea (v1.5)	A03	18,129,318	363	4.00E-141	73.80%	469	BjuA011464		Bna_Rlm9/4/7 ortholog	lost	
369	*At_NDR1 (TM)*	Brassica juncea (v1.5)	A03	22,744,202	220	4.00E-101	79%	330	BjuA012694		At_NDR1 ortholog	lost	
370	*At_BAK1 (LRR-RLK)*	Brassica juncea (v1.5)	A03	26,569,964	156	3.00E-64	73%	238	BjuA012929		At_BAK1 ortholog	lost	
371	*Bol_FocBo1 (TNL)*	Brassica juncea (v1.5)	A03	29,839,949	154	0	81.10%	231	BjuA013581		Bol_FocBo1 ortholog	lost	
372	*Bol_FocBo1 (TNL)*	Brassica juncea (v1.5)	A03	29,844,436	642	0	73.60%	884	BjuA013580		Bol_FocBo1 ortholog	lost	
373	*Bol_FocBo1 (TNL)*	Brassica juncea (v1.5)	A03	29,845,854	311	0	79.40%	489	BjuA013580		Bol_FocBo1 ortholog	lost	
374	*Bol_FocBo1 (TNL)*	Brassica juncea (v1.5)	A03	29,847,029	361	0	95.20%	606	BjuA013580		Bol_FocBo1 ortholog	lost	
375	*Bol_FocBo1 (TNL)*	Brassica juncea (v1.5)	A03	29,847,549	149	0	89.20%	249	BjuA013580		Bol_FocBo1 ortholog	lost	
376	*Bra_cRa/cRb (TNL)*	Brassica juncea (v1.5)	A03	31,636,982	149	6.00E-78	89.90%	290	BjuA012995	Other-RLK	Bra_cRa/cRb ortholog	different	
377	*At_BAK1 (LRR-RLK)*	Brassica juncea (v1.5)	A03	36,957,516	585	0	72.40%	682	BjuA014058		At_BAK1 ortholog	lost	
378	*At_SOBIR1 (LRR-RLK)*	Brassica juncea (v1.5)	A04	19,416,068	505	0	77.60%	714	BjuA016706		At_SOBIR1 ortholog	lost	2
379	*At_SOBIR1 (LRR-RLK)*	Brassica juncea (v1.5)	A04	19,429,333	643	0	77.90%	919	BjuA016707	LRR-RLK	At_SOBIR1 ortholog	same	2
380	*Bna_MAPk (Other-RLK)*	Brassica juncea (v1.5)	A05	2,120,779	297	3.00E-166	72.70%	423	BjuA017849		Bna_MAPk ortholog	lost	
381	*Bju_WRR1 (CNL)*	Brassica juncea (v1.5)	A05	4,784,864	310	0	87.70%	546	BjuA018965	CNL	Bju_WRR1 gene		
382	*Bju_WRR1 (CNL)*	Brassica juncea (v1.5)	A05	4,792,803	578	0	84.20%	929	BjuA018965	CNL	Bju_WRR1 gene		
383	*At_NDR1 (TM)*	Brassica juncea (v1.5)	A05	11,006,149	218	5.00E-100	74.70%	327	BjuA016442		At_NDR1 ortholog	lost	
384	*At_RFO3 (Other-RLK)*	Brassica juncea (v1.5)	A05	23,780,439	350	1.00E-171	71.40%	461	BjuA020243		At_RFO3 ortholog	lost	
385	*At_PBS1 (STK)*	Brassica juncea (v1.5)	A05	31,731,715	221	9.00E-130	74.60%	345	BjuA046137		At_PBS1 ortholog	lost	
386	*Bna_Rlm9/4/7 (Other-RLK)*	Brassica juncea (v1.5)	A06	8,694,726	415	7.00E-141	70.10%	469	BjuA025842	Other-RLK	Bna_Rlm9/4/7 ortholog	same	
387	*At_NRG1a (RNL)*	Brassica juncea (v1.5)	A06	22,322,122	283	0	77.30%	404	BjuA023752	NL	At_NRG1a ortholog	different	
388	*At_NRG1b (RNL)*	Brassica juncea (v1.5)	A06	22,322,122	283	4.00E-175	75.20%	402	BjuA023752	NL	At_NRG1b ortholog	different	
389	*At_NRG1a (RNL)*	Brassica juncea (v1.5)	A07	17,665,979	283	2.00E-102	71%	357	BjuA011771	NL	At_NRG1a ortholog	different	3
390	*At_NRG1b (RNL)*	Brassica juncea (v1.5)	A07	17,666,015	271	6.00E-146	71.90%	367	BjuA011771	NL	At_NRG1b ortholog	different	3
391	*At_NRG1a (RNL)*	Brassica juncea (v1.5)	A07	17,670,864	286	2.00E-98	70.90%	345	BjuA011772		At_NRG1a ortholog	lost	3
392	*At_NRG1b (RNL)*	Brassica juncea (v1.5)	A07	17,670,882	279	1.00E-102	71.30%	357	BjuA011772		At_NRG1b ortholog	lost	3
393	*At_BAK1 (LRR-RLK)*	Brassica juncea (v1.5)	A07	31,213,154	271	1.00E-151	88.10%	493	BjuA043357		At_BAK1 ortholog	lost	
394	*Bra_Crr1a (TNL)*	Brassica juncea (v1.5)	A08	14,472,288	508	0	73.60%	709	BjuA028756		Bra_Crr1a ortholog	lost	
395	*At_BAK1 (LRR-RLK)*	Brassica juncea (v1.5)	A08	16,431,640	181	5.00E-71	83.90%	258	BjuA043694		At_BAK1 ortholog	lost	
396	*At_RFO2 (LRR-RLP)*	Brassica juncea (v1.5)	A08	23,534,428	735	0	71.10%	916	BjuA030513		At_RFO2 ortholog	lost	
397	*At_RFO2 (LRR-RLP)*	Brassica juncea (v1.5)	A08	23,637,098	313	2.00E-143	78.20%	475	BjuA001318		At_RFO2 ortholog	lost	
398	*Bna_Rlm9/4/7 (Other-RLK)*	Brassica juncea (v1.5)	A08	25,411,167	414	4.00E-158	73.60%	520	BjuA031342	Other-RLK	Bna_Rlm9/4/7 ortholog	same	
399	*At_NRG1a (RNL)*	Brassica juncea (v1.5)	A09	6,392,132	217	2.00E-75	74.60%	276	BjuA032766	CNL	At_NRG1a ortholog	different	
400	*At_NRG1a (RNL)*	Brassica juncea (v1.5)	A09	6,393,017	257	3.00E-145	85.20%	439	BjuA032766	CNL	At_NRG1a ortholog	different	
401	*At_NRG1a (RNL)*	Brassica juncea (v1.5)	A09	6,394,301	284	7.00E-129	83%	434	BjuA032766	CNL	At_NRG1a ortholog	different	
402	*At_NRG1b (RNL)*	Brassica juncea (v1.5)	A09	6,394,301	284	9.00E-131	81.60%	440	BjuA032766	CNL	At_NRG1b ortholog	different	
403	*At_RLM1b (TNL)*	Brassica juncea (v1.5)	A09	12,388,588	256	3.00E-179	71.40%	386	BjuA033255		At_RLM1b ortholog	lost	
404	*At_RPS4 (TNL)*	Brassica juncea (v1.5)	A09	18,228,835	186	5.00E-83	77.90%	306	BjuA033777		At_RPS4 ortholog	lost	
405	*At_FLS2 (LRR-RLK)*	Brassica juncea (v1.5)	A09	18,822,456	1029	0	80.60%	1535	BjuA033829	LRR-RLK	At_FLS2 ortholog	same	
406	*Bna_MAPk (Other-RLK)*	Brassica juncea (v1.5)	A09	19,731,990	149	2.00E-55	77.80%	210	BjuA033940		Bna_MAPk ortholog	lost	
407	*Bna_Rlm9/4/7 (Other-RLK)*	Brassica juncea (v1.5)	A09	50,388,643	351	1.00E-139	74.90%	466	BjuA044338	Other-RLK	Bna_Rlm9/4/7 ortholog	same	
408	*At_WRR12 (TNL)*	Brassica juncea (v1.5)	A09	50,739,872	411	6.00E-150	77.10%	519	BjuA044277	TNL	At_WRR12 ortholog	same	4
409	*At_WRR12 (TNL)*	Brassica juncea (v1.5)	A09	50,744,552	368	0	82.30%	547	BjuA044277	TNL	At_WRR12 ortholog	same	4
410	*At_WRR12 (TNL)*	Brassica juncea (v1.5)	A09	50,746,198	521	0	77.90%	820	BjuA044277	TNL	At_WRR12 ortholog	same	4
411	*At_WRR12 (TNL)*	Brassica juncea (v1.5)	A09	50,746,914	149	3.00E-67	84.50%	257	BjuA044277	TNL	At_WRR12 ortholog	same	4
412	*At_WRR12 (TNL)*	Brassica juncea (v1.5)	A09	50,748,511	411	4.00E-144	74.60%	501	BjuA044276	TN	At_WRR12 ortholog	different	4
413	*Bna_Rlm9/4/7 (Other-RLK)*	Brassica juncea (v1.5)	A09	51,149,532	273	2.00E-101	73.90%	353	BjuA044212		Bna_Rlm9/4/7 ortholog	lost	
414	*At_RLP1 (LRR-RLP)*	Brassica juncea (v1.5)	A09	54,409,274	733	0	80.70%	1098	BjuA019470	LRR-RLP	At_RLP1 ortholog	same	
415	*Bna_Rlm9/4/7 (Other-RLK)*	Brassica juncea (v1.5)	A10	4,537,324	423	1.00E-162	73.50%	533	BjuA037454	Other-RLK	Bna_Rlm9/4/7 ortholog	same	
416	*At_PBS1 (STK)*	Brassica juncea (v1.5)	A10	15,795,424	306	2.00E-174	90.10%	552	BjuA039332		At_PBS1 ortholog	lost	
417	*At_RPP13 (CNL)*	Brassica juncea (v1.5)	B01	4,320,770	530	0	76.60%	691	BjuB042097	CN	At_RPP13 ortholog	different	
418	*Bra_Crr1a (TNL)*	Brassica juncea (v1.5)	B01	6,659,984	239	4.00E-119	90.70%	418	BjuB042163		Bra_Crr1a ortholog	lost	
419	*Bra_Crr1a (TNL)*	Brassica juncea (v1.5)	B01	6,662,929	400	6.00E-154	70.70%	523	BjuB042164		Bra_Crr1a ortholog	lost	
420	*At_SOBIR1 (LRR-RLK)*	Brassica juncea (v1.5)	B01	12,586,387	644	0	79.80%	938	BjuB026478	LRR-RLK	At_SOBIR1 ortholog	same	
421	*At_RLP32 (LRR-RLP)*	Brassica juncea (v1.5)	B01	16,125,444	366	0	77.30%	444	BjuB024212		At_RLP32 ortholog	lost	
422	*At_WRR12 (TNL)*	Brassica juncea (v1.5)	B01	25,960,337	214	4.00E-72	71%	273	BjuB024457		At_WRR12 ortholog	lost	
423	*At_NDR1 (TM)*	Brassica juncea (v1.5)	B01	35,663,626	217	8.00E-93	74.60%	306	BjuB026192		At_NDR1 ortholog	lost	
424	*Bna_MAPk (Other-RLK)*	Brassica juncea (v1.5)	B01	38,416,433	268	8.00E-125	75%	357	BjuB025524		Bna_MAPk ortholog	lost	
425	*At_RFO3 (Other-RLK)*	Brassica juncea (v1.5)	B01	40,046,803	184	3.00E-168	77.70%	263	BjuB025614	Other-RLK	At_RFO3 ortholog	same	
426	*At_RFO3 (Other-RLK)*	Brassica juncea (v1.5)	B01	40,047,407	176	3.00E-168	79.50%	286	BjuB025614	Other-RLK	At_RFO3 ortholog	same	
427	*At_NRG1b (RNL)*	Brassica juncea (v1.5)	B02	1,484,226	268	3.00E-100	70.50%	350	BjuB034038	CNL	At_NRG1b ortholog	different	
428	*At_NRG1a (RNL)*	Brassica juncea (v1.5)	B02	1,484,226	263	4.00E-95	70.30%	335	BjuB034038	CNL	At_NRG1a ortholog	different	
429	*At_BAK1 (LRR-RLK)*	Brassica juncea (v1.5)	B02	2,183,030	155	1.00E-67	76.70%	247	BjuB033975		At_BAK1 ortholog	lost	
430	*At_SOBIR1 (LRR-RLK)*	Brassica juncea (v1.5)	B02	20,607,278	339	7.00E-135	77.50%	446	BjuB016108	LRR-RLP	At_SOBIR1 ortholog	different	5
431	*At_SOBIR1 (LRR-RLK)*	Brassica juncea (v1.5)	B02	20,629,147	339	7.00E-135	77.50%	446	BjuB016107		At_SOBIR1 ortholog	lost	5
432	*At_PBS1 (STK)*	Brassica juncea (v1.5)	B02	47,393,413	308	3.00E-175	90.20%	555	BjuB035957		At_PBS1 ortholog	lost	
433	*At_PBS1 (STK)*	Brassica juncea (v1.5)	B02	47,404,539	306	2.00E-174	90.10%	552	BjuB035955		At_PBS1 ortholog	lost	
434	*Bna_LepR3/Rlm2 (LRR-RLP)*	Brassica juncea (v1.5)	B02	47,713,794	940	0	80.60%	1269	BjuB035822		Bna_LepR3/Rlm2 ortholog	lost	
435	*Bol_FocBo1 (TNL)*	Brassica juncea (v1.5)	B02	60,018,077	361	0	95.20%	607	BjuB012951		Bol_FocBo1 ortholog	lost	
436	*Bol_FocBo1 (TNL)*	Brassica juncea (v1.5)	B02	60,019,723	164	2.00E-83	92%	308	BjuB012951		Bol_FocBo1 ortholog	lost	
437	*Bol_FocBo1 (TNL)*	Brassica juncea (v1.5)	B02	60,020,731	166	5.00E-85	94.50%	313	BjuB012951		Bol_FocBo1 ortholog	lost	
438	*Bol_FocBo1 (TNL)*	Brassica juncea (v1.5)	B02	60,021,529	402	0	91.50%	759	BjuB012951		Bol_FocBo1 ortholog	lost	
439	*At_RFO1 (Other-RLK)*	Brassica juncea (v1.5)	B02	62,481,482	350	1.00E-144	72.20%	428	BjuB037876		At_RFO1 ortholog	lost	
440	*At_BAK1 (LRR-RLK)*	Brassica juncea (v1.5)	B02	65,147,594	267	9.00E-156	90.20%	495	BjuB038308		At_BAK1 ortholog	lost	
441	*At_ADR1 (NL)*	Brassica juncea (v1.5)	B03	2,378,135	274	0	70.40%	370	BjuB043228	CNL	At_ADR1 ortholog	different	
442	*At_RFO2 (LRR-RLP)*	Brassica juncea (v1.5)	B03	2,879,415	313	0	78.20%	475	BjuB043336		At_RFO2 ortholog	lost	
443	*At_RFO2 (LRR-RLP)*	Brassica juncea (v1.5)	B03	2,880,632	396	0	78.20%	552	BjuB043336		At_RFO2 ortholog	lost	
444	*Bna_MAPk (Other-RLK)*	Brassica juncea (v1.5)	B03	3,080,966	162	2.00E-62	78.30%	232	BjuB043370		Bna_MAPk ortholog	lost	
445	*At_RPP2a (TNL)*	Brassica juncea (v1.5)	B03	12,412,263	346	6.00E-175	72.80%	496	BjuB004089	TNL	At_RPP2a ortholog	same	
446	*At_RFO3 (Other-RLK)*	Brassica juncea (v1.5)	B03	13,766,542	190	4.00E-70	73.60%	261	BjuB031953		At_RFO3 ortholog	lost	
447	*At_RFO3 (Other-RLK)*	Brassica juncea (v1.5)	B03	13,769,301	190	5.00E-70	73.60%	261	BjuB031953		At_RFO3 ortholog	lost	
448	*At_RFO3 (Other-RLK)*	Brassica juncea (v1.5)	B03	13,769,920	192	2.00E-94	75.50%	284	BjuB031954		At_RFO3 ortholog	lost	
449	*Bna_Rlm9/4/7 (Other-RLK)*	Brassica juncea (v1.5)	B03	14,784,002	306	3.00E-121	71.20%	412	BjuB032313	Other-RLK	Bna_Rlm9/4/7 ortholog	same	
450	*At_WRR12 (TNL)*	Brassica juncea (v1.5)	B03	15,140,053	487	0	78.60%	689	BjuB032367	TX	At_WRR12 ortholog	different	6
451	*At_WRR12 (TNL)*	Brassica juncea (v1.5)	B03	15,142,436	424	0	77.80%	665	BjuB032367	TX	At_WRR12 ortholog	different	6
452	*At_WRR12 (TNL)*	Brassica juncea (v1.5)	B03	15,144,407	149	2.00E-69	86.50%	265	BjuB032367	TX	At_WRR12 ortholog	different	6
453	*At_WRR12 (TNL)*	Brassica juncea (v1.5)	B03	15,146,576	411	6.00E-150	77.10%	519	BjuB032368	Other-NLR	At_WRR12 ortholog	different	6
454	*Bna_Rlm9/4/7 (Other-RLK)*	Brassica juncea (v1.5)	B03	19,240,663	357	7.00E-137	71.10%	457	BjuB047322	Other-RLK	Bna_Rlm9/4/7 ortholog	same	
455	*Bna_MAPk (Other-RLK)*	Brassica juncea (v1.5)	B03	25,023,316	202	1.00E-88	76.20%	300	BjuB047338		Bna_MAPk ortholog	lost	
456	*At_BAK1 (LRR-RLK)*	Brassica juncea (v1.5)	B03	25,490,073	247	4.00E-176	96.70%	506	BjuB047401	LRR-RLK	At_BAK1 ortholog	same	
457	*At_BAK1 (LRR-RLK)*	Brassica juncea (v1.5)	B03	25,976,110	270	4.00E-166	88.50%	491	BjuB005404		At_BAK1 ortholog	lost	
458	*At_BAK1 (LRR-RLK)*	Brassica juncea (v1.5)	B03	26,005,027	270	4.00E-166	88.50%	491	BjuB005401		At_BAK1 ortholog	lost	
459	*At_NRG1b (RNL)*	Brassica juncea (v1.5)	B04	902,503	245	3.00E-100	73.80%	350	BjuB043056	NBS	At_NRG1b ortholog	different	7
460	*At_NRG1a (RNL)*	Brassica juncea (v1.5)	B04	902,509	243	1.00E-96	75.30%	340	BjuB043056	NBS	At_NRG1a ortholog	different	7
461	*At_NRG1a (RNL)*	Brassica juncea (v1.5)	B04	909,959	243	1.00E-96	75.30%	340	BjuB043056	NBS	At_NRG1a ortholog	different	7
462	*At_NRG1b (RNL)*	Brassica juncea (v1.5)	B04	909,965	245	3.00E-100	73.80%	350	BjuB043056	NBS	At_NRG1b ortholog	different	7

463	*At_NRG1b (RNL)*	Brassica juncea (v1.5)	B04	912,060	245	3.00E-100	73.80%	350	BjuB043056	NBS	At_NRG1b ortholog	different	7
464	*At_NRG1a (RNL)*	Brassica juncea (v1.5)	B04	912,066	243	1.00E-96	75.30%	340	BjuB043056	NBS	At_NRG1a ortholog	different	7
465	*At_NRG1a (RNL)*	Brassica juncea (v1.5)	B04	917,648	243	1.00E-96	75.30%	340	BjuB043055	NL	At_NRG1a ortholog	different	7
466	*At_NRG1b (RNL)*	Brassica juncea (v1.5)	B04	917,654	245	3.00E-100	73.80%	350	BjuB043055	NL	At_NRG1b ortholog	different	7
467	*At_FLS2 (LRR-RLK)*	Brassica juncea (v1.5)	B04	4,419,520	1022	0	78.10%	1425	BjuB027601	LRR-RLK	At_FLS2 ortholog	same	
468	*At_RPS4 (TNL)*	Brassica juncea (v1.5)	B04	5,018,312	571	0	70.40%	754	BjuB027664	LRR-RLP	At_RPS4 ortholog	different	
469	*Bna_MAPk (Other-RLK)*	Brassica juncea (v1.5)	B04	6,886,552	149	6.00E-55	77.10%	209	BjuB027817		Bna_MAPk ortholog	lost	
470	*At_FLS2 (LRR-RLK)*	Brassica juncea (v1.5)	B04	7,486,027	1031	0	80.60%	1536	BjuB027897		At_FLS2 ortholog	lost	
471	*At_RLM1b (TNL)*	Brassica juncea (v1.5)	B04	13,810,183	309	2.00E-173	72.40%	458	BjuB028367		At_RLM1b ortholog	lost	
472	*At_RLM1b (TNL)*	Brassica juncea (v1.5)	B04	15,483,265	370	0	71.60%	545	BjuB046985		At_RLM1b ortholog	lost	
473	*At_RLM1b (TNL)*	Brassica juncea (v1.5)	B04	15,494,525	369	7.00E-165	72.60%	552	BjuB046987		At_RLM1b ortholog	lost	
474	*Bna_Rlm9/4/7 (Other-RLK)*	Brassica juncea (v1.5)	B04	16,269,384	420	9.00E-154	71.40%	507	BjuB028598		Bna_Rlm9/4/7 ortholog	lost	
475	*At_BAK1 (LRR-RLK)*	Brassica juncea (v1.5)	B05	3,086,950	268	0	82.40%	433	BjuB045283		At_BAK1 ortholog	lost	
476	*At_RPS2 (NL)*	Brassica juncea (v1.5)	B05	7,458,086	979	0	81.30%	1476	BjuB040328	CNL	At_RPS2 ortholog	different	
477	*At_PBS1 (STK)*	Brassica juncea (v1.5)	B05	11,675,827	311	5.00E-168	86.10%	533	BjuB012316		At_PBS1 ortholog	lost	
478	*At_PBS1 (STK)*	Brassica juncea (v1.5)	B05	11,684,022	311	5.00E-168	86.10%	533	BjuB012315		At_PBS1 ortholog	lost	
479	*At_PBS1 (STK)*	Brassica juncea (v1.5)	B05	11,702,695	311	5.00E-168	86.10%	533	BjuB012314		At_PBS1 ortholog	lost	
480	*At_PBS1 (STK)*	Brassica juncea (v1.5)	B05	18,924,093	221	9.00E-130	74.60%	345	BjuB012564	NL	At_PBS1 ortholog	different	
481	*Bna_Rlm9/4/7 (Other-RLK)*	Brassica juncea (v1.5)	B06	14,519,666	358	8.00E-154	78.40%	507	BjuB021709	Other-RLK	Bna_Rlm9/4/7 ortholog	same	8
482	*Bna_Rlm9/4/7 (Other-RLK)*	Brassica juncea (v1.5)	B06	14,542,596	242	1.00E-110	78.90%	329	BjuB021711	Other-RLK	Bna_Rlm9/4/7 ortholog	same	8
483	*At_RLP1 (LRR-RLP)*	Brassica juncea (v1.5)	B06	17,721,444	489	0	81.30%	711	BjuB015404		At_RLP1 ortholog	lost	
484	*Bna_Rlm9/4/7 (Other-RLK)*	Brassica juncea (v1.5)	B06	22,282,529	414	0	92%	670	BjuB041843	Other-RLK	Bna_Rlm9/4/7 ortholog	same	9
485	*Bna_Rlm9/4/7 (Other-RLK)*	Brassica juncea (v1.5)	B06	22,285,180	318	2.00E-138	73.20%	462	BjuB041843	Other-RLK	Bna_Rlm9/4/7 ortholog	same	9
486	*Bna_Rlm9/4/7 (Other-RLK)*	Brassica juncea (v1.5)	B06	22,288,203	358	3.00E-180	90.70%	575	BjuB041843	Other-RLK	Bna_Rlm9/4/7 ortholog	same	9
487	*Bna_Rlm9/4/7 (Other-RLK)*	Brassica juncea (v1.5)	B06	22,292,175	343	1.00E-162	80.10%	532	BjuB041843	Other-RLK	Bna_Rlm9/4/7 ortholog	same	9
488	*Bna_Rlm9/4/7 (Other-RLK)*	Brassica juncea (v1.5)	B06	22,293,549	419	0	92.10%	682	BjuB041843	Other-RLK	Bna_Rlm9/4/7 ortholog	same	9
489	*Bna_Rlm9/4/7 (Other-RLK)*	Brassica juncea (v1.5)	B06	22,298,537	344	2.00E-132	70.90%	444	BjuB041843	Other-RLK	Bna_Rlm9/4/7 ortholog	same	9
490	*Bna_Rlm9/4/7 (Other-RLK)*	Brassica juncea (v1.5)	B06	22,299,655	503	0	76.70%	640	BjuB041843	Other-RLK	Bna_Rlm9/4/7 ortholog	same	9
491	*Bna_Rlm9/4/7 (Other-RLK)*	Brassica juncea (v1.5)	B06	22,305,058	503	0	76.30%	635	BjuB041843	Other-RLK	Bna_Rlm9/4/7 ortholog	same	9
492	*Bna_Rlm9/4/7 (Other-RLK)*	Brassica juncea (v1.5)	B06	22,306,176	344	7.00E-132	70.60%	443	BjuB041843	Other-RLK	Bna_Rlm9/4/7 ortholog	same	9
493	*Bna_Rlm9/4/7 (Other-RLK)*	Brassica juncea (v1.5)	B06	22,311,222	419	0	90.60%	673	BjuB041844	Other-RLK	Bna_Rlm9/4/7 ortholog	same	9
494	*Bna_Rlm9/4/7 (Other-RLK)*	Brassica juncea (v1.5)	B06	22,312,596	343	1.00E-162	80.10%	532	BjuB041844	Other-RLK	Bna_Rlm9/4/7 ortholog	same	9
495	*Bna_Rlm9/4/7 (Other-RLK)*	Brassica juncea (v1.5)	B06	22,316,568	358	3.00E-180	90.70%	575	BjuB041845	Other-RLK	Bna_Rlm9/4/7 ortholog	same	9
496	*Bna_Rlm9/4/7 (Other-RLK)*	Brassica juncea (v1.5)	B06	22,329,723	299	9.00E-134	80.60%	448	BjuB041845	Other-RLK	Bna_Rlm9/4/7 ortholog	same	9
497	*Bna_Rlm9/4/7 (Other-RLK)*	Brassica juncea (v1.5)	B06	22,482,367	414	0	89.10%	633	BjuB002734		Bna_Rlm9/4/7 ortholog	lost	
498	*Bna_Rlm9/4/7 (Other-RLK)*	Brassica juncea (v1.5)	B06	22,485,937	291	1.00E-132	86.50%	445	BjuB001154		Bna_Rlm9/4/7 ortholog	lost	
499	*Bna_Rlm9/4/7 (Other-RLK)*	Brassica juncea (v1.5)	B06	22,884,657	414	0	94.40%	701	BjuB022106		Bna_Rlm9/4/7 ortholog	lost	
500	*Bna_Rlm9/4/7 (Other-RLK)*	Brassica juncea (v1.5)	B06	22,889,510	345	2.00E-155	78.80%	511	BjuB022107		Bna_Rlm9/4/7 ortholog	lost	
501	*Bna_Rlm9/4/7 (Other-RLK)*	Brassica juncea (v1.5)	B06	33,005,382	424	0	80.60%	602	BjuB046742	Other-RLK	Bna_Rlm9/4/7 ortholog	same	
502	*Bna_Rlm9/4/7 (Other-RLK)*	Brassica juncea (v1.5)	B06	36,515,823	415	0	81.90%	590	BjuB023278	Other-RLK	Bna_Rlm9/4/7 ortholog	same	
503	*Bna_Rlm9/4/7 (Other-RLK)*	Brassica juncea (v1.5)	B06	36,519,097	425	0	79.20%	587	BjuB023278	Other-RLK	Bna_Rlm9/4/7 ortholog	same	
504	*At_NDR1 (TM)*	Brassica juncea (v1.5)	B07	6,447,875	218	7.00E-101	75.60%	329	BjuB007879		At_NDR1 ortholog	lost	
505	*At_NRG1b (RNL)*	Brassica juncea (v1.5)	B07	9,850,429	281	0	71.80%	394	BjuB007476	CNL	At_NRG1b ortholog	different	
506	*At_NRG1a (RNL)*	Brassica juncea (v1.5)	B07	9,850,435	279	0	74.50%	392	BjuB007476	CNL	At_NRG1a ortholog	different	
507	*At_NRG1b (RNL)*	Brassica juncea (v1.5)	B07	10,973,418	294	1.00E-111	74.40%	384	BjuB039514	NL	At_NRG1b ortholog	different	10
508	*At_NRG1a (RNL)*	Brassica juncea (v1.5)	B07	10,973,442	300	2.00E-106	73%	368	BjuB039514	NL	At_NRG1a ortholog	different	10
509	*At_NRG1a (RNL)*	Brassica juncea (v1.5)	B07	10,978,407	164	4.00E-108	77.40%	239	BjuB039514	NL	At_NRG1a ortholog	different	10
510	*At_NRG1b (RNL)*	Brassica juncea (v1.5)	B07	10,978,407	164	1.00E-110	77.40%	234	BjuB039514	NL	At_NRG1b ortholog	different	10
511	*At_NRG1a (RNL)*	Brassica juncea (v1.5)	B07	10,986,810	378	2.00E-151	74.60%	501	BjuB039515	CNL	At_NRG1a ortholog	different	10
512	*At_NRG1b (RNL)*	Brassica juncea (v1.5)	B07	10,986,810	378	3.00E-152	73.20%	503	BjuB039515	CNL	At_NRG1b ortholog	different	10
513	*At_NRG1a (RNL)*	Brassica juncea (v1.5)	B07	10,988,619	371	0	71.40%	471	BjuB039515	CNL	At_NRG1a ortholog	different	10
514	*Bna_Rlm9/4/7 (Other-RLK)*	Brassica juncea (v1.5)	B07	14,971,695	414	2.00E-164	74.80%	538	BjuB026813		Bna_Rlm9/4/7 ortholog	lost	
515	*At_NDR1 (TM)*	Brassica juncea (v1.5)	B07	16,041,662	219	4.00E-104	79.40%	338	BjuB008693		At_NDR1 ortholog	lost	
516	*At_PBS1 (STK)*	Brassica juncea (v1.5)	B08	9,043,761	221	3.00E-100	77.30%	338	BjuB014768		At_PBS1 ortholog	lost	
517	*Bra_Crr1a (TNL)*	Brassica juncea (v1.5)	B08	13,443,718	290	3.00E-118	73.10%	416	BjuB015637		Bra_Crr1a ortholog	lost	
518	*At_RPP2b (TNL)*	Brassica juncea (v1.5)	B08	22,878,689	216	0	76.30%	368	BjuB046002	TNL	At_RPP2b ortholog	same	11
519	*At_RPP2b (TNL)*	Brassica juncea (v1.5)	B08	22,882,180	150	5.00E-56	72%	219	BjuB046002	TNL	At_RPP2b ortholog	same	11
520	*At_RPP2a (TNL)*	Brassica juncea (v1.5)	B08	22,885,460	352	0	73.20%	526	BjuB046003		At_RPP2a ortholog	lost	11
521	*Bna_MAPk (Other-RLK)*	Brassica juncea (v1.5)	B08	25,383,730	213	3.00E-155	70.80%	278	BjuB016962		Bna_MAPk ortholog	lost	
522	*At_RPP13 (CNL)*	Brassica juncea (v1.5)	B08	55,028,163	184	0	75%	202	BjuB019597		At_RPP13 ortholog	lost	
523	*At_SOBIR1 (LRR-RLK)*	Brassica juncea (v1.5)	Contig10400	13,067	647	0	80%	976	BjuO000539	LRR-RLK	At_SOBIR1 ortholog	same	
524	*At_RFO3 (Other-RLK)*	Brassica juncea (v1.5)	Contig11_2007833_2318	232,793	192	2.00E-94	75.50%	284	BjuO001166	Other-NLR	At_RFO3 ortholog	different	
525	*At_RFO3 (Other-RLK)*	Brassica juncea (v1.5)	Contig11_2007833_2318	233,412	190	5.00E-70	73.60%	261	BjuO001166	Other-NLR	At_RFO3 ortholog	different	
526	*At_RFO3 (Other-RLK)*	Brassica juncea (v1.5)	Contig11_2007833_2318	236,171	190	1.00E-69	73.60%	260	BjuO001166	Other-NLR	At_RFO3 ortholog	different	
527	*At_RPP8 (CNL)*	Brassica juncea (v1.5)	Contig304_976566_1213	165,576	293	5.00E-104	73.70%	364	BjuO004937	NL	At_RPP8 ortholog	different	
528	*Bna_Rlm9/4/7 (Other-RLK)*	Brassica juncea (v1.5)	Contig36_1_2224133_45	9,502	223	5.00E-76	70.80%	277	BjuO005777	Other-RLK	Bna_Rlm9/4/7 ortholog	same	
529	*Bna_Rlm9/4/7 (Other-RLK)*	Brassica juncea (v1.5)	Contig36_1_2224133_45	14,282	414	1.00E-157	73.40%	518	BjuO005778		Bna_Rlm9/4/7 ortholog	lost	
530	*At_RPM1 (NL)*	Brassica juncea (v1.5)	Contig3612	120,430	473	0	81.10%	749	BjuO005682		At_RPM1 ortholog	lost	
531	*At_RPM1 (NL)*	Brassica juncea (v1.5)	Contig3612	121,991	533	0	75%	736	BjuO005683	NBS	At_RPM1 ortholog	different	
532	*Bra_cRa/cRb (TNL)*	Brassica juncea (v1.5)	Contig371_150000_5894	279,095	374	9.00E-172	75.60%	578	BjuO005872		Bra_cRa/cRb ortholog	lost	
533	*Bra_cRa/cRb (TNL)*	Brassica juncea (v1.5)	Contig371_150000_5894	279,095	374	9.00E-172	75.60%	578	BjuO005872		Bra_cRa/cRb ortholog	lost	
534	*Bra_cRa/cRb (TNL)*	Brassica juncea (v1.5)	Contig371_150000_5894	279,865	149	1.00E-77	89.20%	289	BjuO005872		Bra_cRa/cRb ortholog	lost	
535	*Bra_cRa/cRb (TNL)*	Brassica juncea (v1.5)	Contig371_150000_5894	290,097	220	5.00E-80	74.00%	297	BjuO005873		Bra_cRa/cRb ortholog	lost	
536	*At_RPP13 (CNL)*	Brassica juncea (v1.5)	Contig4032	13,758	240	0	75%	287	BjuO006220	CN	At_RPP13 ortholog	different	
537	*Bju_WRR1 (CNL)*	Brassica juncea (v1.5)	Contig467_246521_1152	31,358	281	1.00E-117	74%	374	BjuO007002	TX	Bju_WRR1 paralog (segmented)	different	
538	*Bna_Rlm9/4/7 (Other-RLK)*	Brassica juncea (v1.5)	Contig958	121,997	414	4.00E-158	73.60%	520	BjuO009876	Other-RLK	Bna_Rlm9/4/7 ortholog	same	
539	*At_BAK1 (LRR-RLK)*	Brassica juncea (v1.5)	Super_scaffold_35_9066	36,992	157	2.00E-62	71.90%	232	BjuO012447		At_BAK1 ortholog	lost	
540	*At_RPS2 (NL)*	Brassica napus (v4.1)	A01	7,809,061	824	0	81.90%	1293	BnaA01g15270D	CNL	At_RPS2 ortholog	different	
541	*At_RPM1 (NL)*	Brassica napus (v4.1)	A01	21,882,052	538	0	76.70%	775	BnaA01g32500D		At_RPM1 ortholog	lost	
542	*At_RPM1 (NL)*	Brassica napus (v4.1)	A01	21,883,684	755	0	79%	1177	BnaA01g32500D		At_RPM1 ortholog	lost	
543	*At_NDR1 (TM)*	Brassica napus (v4.1)	A01_random	2,274,848	218	3.00E-100	74.70%	327	BnaA01g36770D		At_NDR1 ortholog	lost	
544	*At_PBS1 (STK)*	Brassica napus (v4.1)	A02	650,604	152	1.00E-147	90.10%	268	BnaA02g01440D		At_PBS1 ortholog	lost	
545	*At_RPS4 (TNL)*	Brassica napus (v4.1)	A02	17,105,912	294	0	81.90%	461	BnaA02g23810D	NL	At_RPS4 ortholog	different	1
546	*At_RRS1 (TNL)*	Brassica napus (v4.1)	A02	17,140,309	371	0	70.80%	516	BnaA02g23840D	TNL	At_RRS1 ortholog	same	1
547	*At_FLS2 (LRR-RLK)*	Brassica napus (v4.1)	A02	17,797,445	509	0	75%	733	BnaA02g24430D		At_FLS2 ortholog	lost	2
548	*At_FLS2 (LRR-RLK)*	Brassica napus (v4.1)	A02	17,798,970	362	0	76.50%	426	BnaA02g24440D	LRR-RLP	At_FLS2 ortholog	different	2
549	*Bol_FocBo1 (TNL)*	Brassica napus (v4.1)	A02	21,951,695	150	1.00E-175	74%	223	BnaA02g30300D	TNL	Bol_FocBo1 ortholog	same	
550	*At_NRG1a (RNL)*	Brassica napus (v4.1)	A02	22,632,504	244	1.00E-93	72.90%	330	BnaA02g31380D	NL	At_NRG1a ortholog	different	
551	*At_NRG1b (RNL)*	Brassica napus (v4.1)	A02	22,632,510	249	1.00E-97	71%	342	BnaA02g31380D	NL	At_NRG1b ortholog	different	
552	*At_PBS1 (STK)*	Brassica napus (v4.1)	A03	1,951,315	160	7.00E-65	75.60%	234	BnaA03g04220D		At_PBS1 ortholog	lost	
553	*At_PBS1 (STK)*	Brassica napus (v4.1)	A03	1,952,081	323	1.00E-154	77.70%	495	BnaA03g04220D		At_PBS1 ortholog	lost	
554	*At_SOBIR1 (LRR-RLK)*	Brassica napus (v4.1)	A03	6,784,297	613	0	79.60%	961	BnaA03g14760D	LRR-RLK	At_SOBIR1 ortholog	same	
555	*At_NDR1 (TM)*	Brassica napus (v4.1)	A03	17,509,529	220	2.00E-96	78.60%	316	BnaA03g35850D		At_NDR1 ortholog	lost	
556	*Bra_cRa/cRb (TNL)*	Brassica napus (v4.1)	A03	22,866,351	548	0	70.80%	713	BnaA03g45000D	TNL	Bra_cRa/cRb ortholog	same	3
557	*Bra_cRa/cRb (TNL)*	Brassica napus (v4.1)	A03	22,866,351	548	0.00E+00	76.40%	781	BnaA03g45000D	TNL	Bra_cRa/cRb ortholog	same	3
558	*Bra_cRa/cRb (TNL)*	Brassica napus (v4.1)	A03	22,867,981	498	0	91.30%	942	BnaA03g45000D	TNL	Bra_cRa/cRb ortholog	same	3
559	*Bra_cRa/cRb (TNL)*	Brassica napus (v4.1)	A03	22,867,981	498	0.00E+00	93%	962	BnaA03g45000D	TNL	Bra_cRa/cRb ortholog	same	3
560	*Bra_cRa/cRb (TNL)*	Brassica napus (v4.1)	A03	22,872,209	211	6.00E-95	90.50%	345	BnaA03g45000D	TNL	Bra_cRa/cRb ortholog	same	3
561	*Bra_cRa/cRb (TNL)*	Brassica napus (v4.1)	A03	22,877,861	373	0	79.60%	608	BnaA03g45020D	Other-NLR	Bra_cRa/cRb ortholog	different	3
562	*Bra_cRa/cRb (TNL)*	Brassica napus (v4.1)	A03	22,877,861	373	0.00E+00	83.10%	639	BnaA03g45020D	Other-NLR	Bra_cRa/cRb ortholog	different	3
563	*Bra_cRa/cRb (TNL)*	Brassica napus (v4.1)	A03	22,878,662	149	8.00E-77	88.50%	286	BnaA03g45020D	Other-NLR	Bra_cRa/cRb ortholog	different	3
564	*Bra_cRa/cRb (TNL)*	Brassica napus (v4.1)	A03	22,888,887	149	2.00E-70	83.80%	266	BnaA03g45040D	TN	Bra_cRa/cRb ortholog	different	3
565	*At_BAK1 (LRR-RLK)*	Brassica napus (v4.1)	A03	27,408,858	406	0	81.20%	653	BnaA03g52520D	LRR-RLK	At_BAK1 ortholog	same	
566	*At_SOBIR1 (LRR-RLK)*	Brassica napus (v4.1)	A04	14,792,368	612	0	75.10%	867	BnaA04g18590D	LRR-RLK	At_SOBIR1 ortholog	same	4
567	*At_SOBIR1 (LRR-RLK)*	Brassica napus (v4.1)	A04	14,806,040	609	0	78.10%	906	BnaA04g18600D	LRR-RLK	At_SOBIR1 ortholog	same	4
568	*Bna_MAPk (Other-RLK)*	Brassica napus (v4.1)	A05	1,709,039	207	2.00E-160	73.90%	292	BnaA05g03140D		Bna_MAPk paralog (segmented)	lost	
569	*Bju_WRR1 (CNL)*	Brassica napus (v4.1)	A05	4,305,311	311	2.00E-162	80.70%	495	BnaA05g07870D	CNL	Bju_WRR1 ortholog	same	5
570	*Bju_WRR1 (CNL)*	Brassica napus (v4.1)	A05	4,311,474	582	0	80.70%	895	BnaA05g07870D	CNL	Bju_WRR1 ortholog	same	5
571	*Bju_WRR1 (CNL)*	Brassica napus (v4.1)	A05	4,319,877	382	5.00E-179	81.90%	611	BnaA05g07880D	CNL	Bju_WRR1 ortholog	same	5
572	*Bju_WRR1 (CNL)*	Brassica napus (v4.1)	A05	4,321,481	574	0	75.90%	816	BnaA05g07880D	CNL	Bju_WRR1 ortholog	same	5
573	*Bju_WRR1 (CNL)*	Brassica napus (v4.1)	A05	4,332,312	299	4.00E-164	80.60%	483	BnaA05g07900D	CNL	Bju_WRR1 ortholog	same	5
574	*Bju_WRR1 (CNL)*	Brassica napus (v4.1)	A05	4,337,193	573	0	76%	828	BnaA05g07900D	CNL	Bju_WRR1 ortholog	same	5
575	*Bju_WRR1 (CNL)*	Brassica napus (v4.1)	A05	4,355,848	295	5.00E-152	77.60%	449	BnaA05g07930D	CNL	Bju_WRR1 ortholog	same	5
576	*Bju_WRR1 (CNL)*	Brassica napus (v4.1)	A05	4,364,235	587	0	78.80%	862	BnaA05g07930D	CNL	Bju_WRR1 ortholog	same	5
577	*Bna_MAPk (Other-RLK)*	Brassica napus (v4.1)	A05	8,893,301	222	6.00E-88	70.70%	306	BnaA05g14400D		Bna_MAPk paralog (segmented)	lost	
578	*At_RFO2 (LRR-RLP)*	Brassica napus (v4.1)	A06	6,021,550	264	2.00E-100	70.40%	327	BnaA06g11590D		At_RFO2 ortholog	lost	
579	*Bna_MAPk (Other-RLK)*	Brassica napus (v4.1)	A06	6,410,020	204	3.00E-115	78.90%	306	BnaA06g12330D		Bna_MAPk paralog (segmented)	lost	

580	*At_NRG1a (RNL)*	Brassica napus (v4.1)	A06	19,271,016	278	9.00E-179	75.50%	394	BnaA06g28070D	NL	At_NRG1a ortholog	different	
581	*At_NRG1b (RNL)*	Brassica napus (v4.1)	A06	19,271,016	278	6.00E-171	74.40%	399	BnaA06g28070D	NL	At_NRG1b ortholog	different	
582	*Bju_WRR1 (CNL)*	Brassica napus (v4.1)	A06	20,318,707	318	6.00E-131	70.70%	446	BnaA06g29890D	CN	Bju_WRR1 ortholog	different	
583	*At_NRG1a (RNL)*	Brassica napus (v4.1)	A07	11,205,908	267	6.00E-142	70.40%	330	BnaA07g12270D	NL	At_NRG1a ortholog	different	
584	*Bna_Rlm9/4/7 (Other-RLK)*	Brassica napus (v4.1)	A07	15,912,812	403	0	87.50%	708	BnaA07g20220D	Other-RLK	Bna_Rlm9/4/7 gene		
585	*Bna_Rlm9/4/7 (Other-RLK)*	Brassica napus (v4.1)	A07	15,914,139	414	0	90%	659	BnaA07g20220D	Other-RLK	Bna_Rlm9/4/7 gene		
586	*At_BAK1 (LRR-RLK)*	Brassica napus (v4.1)	A07	17,609,967	283	3.00E-171	86.90%	509	BnaA07g23390D	LRR-RLK	At_BAK1 ortholog	same	
587	*At_BAK1 (LRR-RLK)*	Brassica napus (v4.1)	A07	21,146,877	271	5.00E-151	88.10%	491	BnaA07g29610D	LRR-RLK	At_BAK1 ortholog	same	
588	*Bna_Rlm9/4/7 (Other-RLK)*	Brassica napus (v4.1)	A07	23,714,980	282	0	70.20%	337	BnaA07g35090D		Bna_Rlm9/4/7 paralog (segmente	lost	
589	*Bra_Crr1a (TNL)*	Brassica napus (v4.1)	A08	9,456,600	171	3.00E-103	100%	370	BnaA08g10100D	TNL	Bra_Crr1a ortholog	same	
590	*Bra_Crr1a (TNL)*	Brassica napus (v4.1)	A08	9,458,599	365	0	95.30%	618	BnaA08g10100D	TNL	Bra_Crr1a ortholog	same	
591	*Bra_Crr1a (TNL)*	Brassica napus (v4.1)	A08	9,460,211	504	0	82.90%	808	BnaA08g10100D	TNL	Bra_Crr1a ortholog	same	
592	*At_NRG1a (RNL)*	Brassica napus (v4.1)	A09	3,765,226	217	2.00E-75	74.60%	276	BnaA09g07700D	CNL	At_NRG1a ortholog	different	
593	*At_NRG1a (RNL)*	Brassica napus (v4.1)	A09	3,766,310	351	9.00E-154	75.70%	507	BnaA09g07700D	CNL	At_NRG1a ortholog	different	
594	*At_NRG1a (RNL)*	Brassica napus (v4.1)	A09	3,767,507	314	9.00E-114	73.20%	390	BnaA09g07700D	CNL	At_NRG1a ortholog	different	
595	*At_NRG1b (RNL)*	Brassica napus (v4.1)	A09	3,767,615	278	6.00E-115	79.10%	394	BnaA09g07700D	CNL	At_NRG1b ortholog	different	
596	*At_FLS2 (LRR-RLK)*	Brassica napus (v4.1)	A09	11,052,516	1029	0	79.80%	1521	BnaA09g17950D	LRR-RLK	At_FLS2 ortholog	same	
597	*Bna_MAPk (Other-RLK)*	Brassica napus (v4.1)	A09	11,574,577	149	1.00E-55	77.80%	211	BnaA09g18630D		Bna_MAPk paralog (segmented)	lost	
598	*At_WRR12 (TNL)*	Brassica napus (v4.1)	A09	30,772,200	363	0	79%	516	BnaA09g44880D	NL	At_WRR12 ortholog	different	6
599	*At_WRR12 (TNL)*	Brassica napus (v4.1)	A09	30,773,786	368	0	87.20%	665	BnaA09g44890D	TNL	At_WRR12 ortholog	same	6
600	*At_WRR12 (TNL)*	Brassica napus (v4.1)	A09	30,774,573	149	3.00E-67	84.50%	257	BnaA09g44890D	TNL	At_WRR12 ortholog	same	6
601	*At_WRR12 (TNL)*	Brassica napus (v4.1)	A09	30,776,168	401	2.00E-133	71.50%	469	BnaA09g44910D	NBS	At_WRR12 ortholog	different	6
602	*At_RLP1 (LRR-RLP)*	Brassica napus (v4.1)	A09	32,933,162	732	0	79.30%	1081	BnaA09g49510D		At_RLP1 ortholog	lost	
603	*Bna_MAPk (Other-RLK)*	Brassica napus (v4.1)	A10	12,328,399	205	2.00E-158	75.10%	286	BnaA10g16050D		Bna_MAPk paralog (segmented)	lost	
604	*At_PBS1 (STK)*	Brassica napus (v4.1)	A10	14,132,154	306	2.00E-174	90.10%	552	BnaA10g20130D		At_PBS1 ortholog	lost	7
605	*Bna_LepR3/Rlm2 (LRR-RLP)*	Brassica napus (v4.1)	A10	14,408,228	938	0	94.60%	1568	BnaA10g20720D	LRR-RLP	Bna_LepR3/Rlm2 gene		7
606	*At_PBS1 (STK)*	Brassica napus (v4.1)	Ann_random	441,036	216	9.00E-131	76.30%	346	BnaAnng00490D		At_PBS1 ortholog	lost	
607	*At_BAK1 (LRR-RLK)*	Brassica napus (v4.1)	Ann_random	10,492,141	157	2.00E-123	71.30%	230	BnaAnng09800D	LRR-RLK	At_BAK1 ortholog	same	
608	*Bju_WRR1 (CNL)*	Brassica napus (v4.1)	Ann_random	29,698,468	574	0	78.30%	854	BnaAnng25650D	Other-RLK	Bju_WRR1 ortholog	different	
609	*At_BAK1 (LRR-RLK)*	Brassica napus (v4.1)	C01	2,492,169	271	0	88.90%	496	BnaC01g04780D	LRR-RLK	At_BAK1 ortholog	same	
610	*At_RPP2a (TNL)*	Brassica napus (v4.1)	C01	7,475,079	347	0	71.10%	499	BnaC01g11870D	TNL	At_RPP2a ortholog	same	
611	*At_PBS1 (STK)*	Brassica napus (v4.1)	C01	10,547,452	218	3.00E-118	70.10%	322	BnaC01g15420D		At_PBS1 ortholog	lost	
612	*At_RPS2 (NL)*	Brassica napus (v4.1)	C01	12,578,108	926	0	80.30%	1433	BnaC01g18040D	NL	At_RPS2 ortholog	same	8
613	*At_RPS2 (NL)*	Brassica napus (v4.1)	C01	12,580,855	287	2.00E-116	71.40%	408	BnaC01g18050D	LRR-RLK	At_RPS2 ortholog	different	8
614	*At_RFO3 (Other-RLK)*	Brassica napus (v4.1)	C01_random	3,874,474	161	2.00E-100	73.90%	237	BnaC01g44280D	Other-RLK	At_RFO3 ortholog	same	
615	*At_PBS1 (STK)*	Brassica napus (v4.1)	C02	2,366,207	317	1.00E-163	83.90%	521	BnaC02g04520D		At_PBS1 ortholog	lost	
616	*At_FLS2 (LRR-RLK)*	Brassica napus (v4.1)	C02	34,764,682	328	1.00E-132	72.20%	457	BnaC02g32260D	LRR-RLK	At_FLS2 ortholog	same	
617	*At_FLS2 (LRR-RLK)*	Brassica napus (v4.1)	C02	34,765,631	714	0	77.30%	974	BnaC02g32260D	LRR-RLK	At_FLS2 ortholog	same	
618	*Bra_cRa/cRb (TNL)*	Brassica napus (v4.1)	C03	5,911,520	149	3.00E-67	79.10%	256	BnaC03g12220D	TNL	Bra_cRa/cRb ortholog	same	
619	*At_SOBIR1 (LRR-RLK)*	Brassica napus (v4.1)	C03	9,089,357	613	0	78.10%	952	BnaC03g17800D	LRR-RLK	At_SOBIR1 ortholog	same	
620	*At_RLP32 (LRR-RLP)*	Brassica napus (v4.1)	C03	16,182,943	422	2.00E-159	70.10%	526	BnaC03g27740D	LRR-RLP	At_RLP32 ortholog	same	
621	*At_NDR1 (TM)*	Brassica napus (v4.1)	C03	26,585,925	220	4.00E-100	78.10%	327	BnaC03g41690D		At_NDR1 ortholog	lost	
622	*At_RFO3 (Other-RLK)*	Brassica napus (v4.1)	C03	28,016,588	185	9.00E-105	75.60%	259	BnaC03g43200D	Other-RLK	At_RFO3 ortholog	same	
623	*At_RPP2b (TNL)*	Brassica napus (v4.1)	C03	52,670,102	159	3.00E-81	83.60%	300	BnaC03g63290D	TNL	At_RPP2b ortholog	same	
624	*At_RPP2b (TNL)*	Brassica napus (v4.1)	C03	52,670,487	257	0	73.90%	313	BnaC03g63290D	TNL	At_RPP2b ortholog	same	
625	*At_RPP2b (TNL)*	Brassica napus (v4.1)	C03	52,671,338	216	0	80.50%	386	BnaC03g63290D	TNL	At_RPP2b ortholog	same	
626	*Bra_Crr1a (TNL)*	Brassica napus (v4.1)	C03	54,091,253	339	0	78.10%	505	BnaC03g64750D	Other-NLR	Bra_Crr1a ortholog	different	
627	*Bra_Crr1a (TNL)*	Brassica napus (v4.1)	C03	54,093,518	372	0	84.60%	623	BnaC03g64750D	Other-NLR	Bra_Crr1a ortholog	different	
628	*At_BAK1 (LRR-RLK)*	Brassica napus (v4.1)	C03	56,311,726	181	6.00E-72	84.50%	260	BnaC03g66580D	LRR-RLK	At_BAK1 ortholog	same	
629	*At_PBS1 (STK)*	Brassica napus (v4.1)	C03_random	182,236	322	4.00E-153	79.10%	491	BnaC03g71490D		At_PBS1 ortholog	lost	
630	*At_PBS1 (STK)*	Brassica napus (v4.1)	C03_random	183,009	160	7.00E-67	78.10%	240	BnaC03g71490D		At_PBS1 ortholog	lost	
631	*Bju_WRR1 (CNL)*	Brassica napus (v4.1)	C04	6,689,765	306	4.00E-167	80%	497	BnaC04g08930D	CNL	Bju_WRR1 ortholog	same	
632	*Bju_WRR1 (CNL)*	Brassica napus (v4.1)	C04	6,691,309	501	0	77%	709	BnaC04g08930D	CNL	Bju_WRR1 ortholog	same	
633	*At_RFO3 (Other-RLK)*	Brassica napus (v4.1)	C05	36,364,003	290	8.00E-158	71%	381	BnaC05g37330D	Other-RLK	At_RFO3 ortholog	same	
634	*Bna_Rlm9/4/7 (Other-RLK)*	Brassica napus (v4.1)	C05_random	670,856	258	3.00E-110	74.80%	325	BnaC05g49950D		Bna_Rlm9/4/7 paralog (segmente	lost	
635	*Bna_MAPk (Other-RLK)*	Brassica napus (v4.1)	C05_random	765,421	155	8.00E-153	81.20%	230	BnaC05g50100D		Bna_MAPk paralog (segmented)	lost	
636	*Bna_Rlm9/4/7 (Other-RLK)*	Brassica napus (v4.1)	C06	21,847,811	343	3.00E-152	77.20%	502	BnaC06g19670D	Other-RLK	Bna_Rlm9/4/7 paralog (segmente	same	9
637	*Bna_Rlm9/4/7 (Other-RLK)*	Brassica napus (v4.1)	C06	21,853,061	405	0	86.60%	610	BnaC06g19670D	Other-RLK	Bna_Rlm9/4/7 paralog (segmente	same	9
638	*Bna_Rlm9/4/7 (Other-RLK)*	Brassica napus (v4.1)	C06	21,873,027	322	0	81%	537	BnaC06g19690D	Other-RLK	Bna_Rlm9/4/7 paralog (segmente	same	9
639	*Bna_Rlm9/4/7 (Other-RLK)*	Brassica napus (v4.1)	C06	21,874,108	468	0	79.90%	639	BnaC06g19690D	Other-RLK	Bna_Rlm9/4/7 paralog (segmente	same	9
640	*At_BAK1 (LRR-RLK)*	Brassica napus (v4.1)	C07	6,913,900	157	2.00E-123	71.30%	230	BnaC07g04440D	LRR-RLK	At_BAK1 ortholog	same	
641	*At_NRG1a (RNL)*	Brassica napus (v4.1)	C07	22,454,472	273	2.00E-95	71%	336	BnaC07g16470D	CNL	At_NRG1a ortholog	different	
642	*Bju_WRR1 (CNL)*	Brassica napus (v4.1)	C07	32,597,157	317	1.00E-134	70.30%	446	BnaC07g26860D	CNL	Bju_WRR1 ortholog	same	
643	*Bol_FocBo1 (TNL)*	Brassica napus (v4.1)	C07	36,966,687	402	0	88.80%	728	BnaC07g33990D		Bol_FocBo1 ortholog	lost	10
644	*Bol_FocBo1 (TNL)*	Brassica napus (v4.1)	C07	36,967,412	164	1.00E-75	87.80%	283	BnaC07g33990D		Bol_FocBo1 ortholog	lost	10
645	*Bol_FocBo1 (TNL)*	Brassica napus (v4.1)	C07	36,968,412	164	0	95.70%	320	BnaC07g33990D		Bol_FocBo1 ortholog	lost	10
646	*Bol_FocBo1 (TNL)*	Brassica napus (v4.1)	C07	36,969,987	323	0	89.10%	498	BnaC07g34000D	TN	Bol_FocBo1 ortholog	different	10
647	*Bol_FocBo1 (TNL)*	Brassica napus (v4.1)	C07	36,970,534	149	0	99.30%	276	BnaC07g34000D	TN	Bol_FocBo1 ortholog	different	10
648	*Bra_cRa/cRb (TNL)*	Brassica napus (v4.1)	C07	38,866,602	370	3.00E-163	72.90%	552	BnaC07g37000D	TNL	Bra_cRa/cRb ortholog	same	11
649	*Bra_cRa/cRb (TNL)*	Brassica napus (v4.1)	C07	38,866,602	370	2.00E-179	78%	600	BnaC07g37000D	TNL	Bra_cRa/cRb ortholog	same	11
650	*Bra_cRa/cRb (TNL)*	Brassica napus (v4.1)	C07	38,883,868	149	1.00E-70	83.80%	266	BnaC07g37010D	TNL	Bra_cRa/cRb ortholog	same	11
651	*At_BAK1 (LRR-RLK)*	Brassica napus (v4.1)	C07	42,893,162	260	3.00E-153	90%	481	BnaC07g44250D	LRR-RLK	At_BAK1 ortholog	same	
652	*Bna_Rlm9/4/7 (Other-RLK)*	Brassica napus (v4.1)	C08	736,593	299	2.00E-110	73.20%	380	BnaC08g01040D		Bna_Rlm9/4/7 paralog (segmente	lost	
653	*Bol_FocBo1 (TNL)*	Brassica napus (v4.1)	C08	16,227,708	253	0	72.30%	296	BnaC08g10820D		Bol_FocBo1 ortholog	lost	
654	*Bna_Rlm9/4/7 (Other-RLK)*	Brassica napus (v4.1)	C08	33,846,247	178	2.00E-103	75.20%	220	BnaC08g36740D	Other-RLK	Bna_Rlm9/4/7 paralog (segmente	same	
655	*Bna_MAPk (Other-RLK)*	Brassica napus (v4.1)	C08	34,220,131	201	1.00E-153	80%	311	BnaC08g37390D		Bna_MAPk paralog (segmented)	lost	
656	*At_WRR12 (TNL)*	Brassica napus (v4.1)	C08	34,304,231	363	0	80.90%	539	BnaC08g37590D	TNL	At_WRR12 ortholog	same	12
657	*At_WRR12 (TNL)*	Brassica napus (v4.1)	C08	34,305,842	374	0	84.20%	618	BnaC08g37590D	TNL	At_WRR12 ortholog	same	12
658	*At_WRR12 (TNL)*	Brassica napus (v4.1)	C08	34,308,259	401	1.00E-136	72.50%	479	BnaC08g37610D	NBS	At_WRR12 ortholog	different	12
659	*At_WRR12 (TNL)*	Brassica napus (v4.1)	C08	34,313,443	272	2.00E-137	80.80%	374	BnaC08g37630D	NL	At_WRR12 ortholog	different	12
660	*At_RLP1 (LRR-RLP)*	Brassica napus (v4.1)	C08	38,027,292	370	0	85.40%	555	BnaC08g45290D	LRR-RLP	At_RLP1 ortholog	same	
661	*At_RLP1 (LRR-RLP)*	Brassica napus (v4.1)	C08	38,028,398	368	0	72.50%	506	BnaC08g45290D	LRR-RLP	At_RLP1 ortholog	same	
662	*At_RPP13 (CNL)*	Brassica napus (v4.1)	C09	4,673,917	169	0	71%	219	BnaC09g07440D	NL	At_RPP13 ortholog	different	
663	*At_NRG1a (RNL)*	Brassica napus (v4.1)	C09	4,777,453	284	4.00E-115	72.50%	394	BnaC09g07660D	NL	At_NRG1a ortholog	different	13
664	*At_NRG1a (RNL)*	Brassica napus (v4.1)	C09	4,778,823	279	6.00E-139	81.70%	422	BnaC09g07660D	NL	At_NRG1a ortholog	different	13
665	*At_NRG1b (RNL)*	Brassica napus (v4.1)	C09	4,778,823	279	5.00E-140	80.60%	428	BnaC09g07660D	NL	At_NRG1b ortholog	different	13
666	*At_NRG1a (RNL)*	Brassica napus (v4.1)	C09	4,796,772	217	7.00E-71	70.90%	262	BnaC09g07690D	CNL	At_NRG1a ortholog	different	13
667	*At_NRG1a (RNL)*	Brassica napus (v4.1)	C09	4,797,636	257	6.00E-143	82.40%	426	BnaC09g07690D	CNL	At_NRG1a ortholog	different	13
668	*At_NRG1a (RNL)*	Brassica napus (v4.1)	C09	4,798,941	278	2.00E-110	78.40%	380	BnaC09g07690D	CNL	At_NRG1a ortholog	different	13
669	*At_NRG1b (RNL)*	Brassica napus (v4.1)	C09	4,798,941	278	9.00E-113	77.60%	387	BnaC09g07690D	CNL	At_NRG1b ortholog	different	13
670	*At_RLM1b (TNL)*	Brassica napus (v4.1)	C09	8,175,957	372	0	72%	541	BnaC09g11670D	NL	At_RLM1b ortholog	different	
671	*At_RPS4 (TNL)*	Brassica napus (v4.1)	C09	15,829,258	189	5.00E-76	71.40%	284	BnaC09g18950D	TNL	At_RPS4 ortholog	same	
672	*At_RPS4 (TNL)*	Brassica napus (v4.1)	C09	15,830,646	425	0	75.50%	621	BnaC09g18950D	TNL	At_RPS4 ortholog	same	
673	*At_FLS2 (LRR-RLK)*	Brassica napus (v4.1)	C09	16,171,976	1029	0	79.50%	1504	BnaC09g19310D	LRR-RLK	At_FLS2 ortholog	same	
674	*Bna_MAPk (Other-RLK)*	Brassica napus (v4.1)	C09	41,611,615	203	2.00E-162	78.80%	305	BnaC09g38840D		Bna_MAPk paralog (segmented)	lost	
675	*At_PBS1 (STK)*	Brassica napus (v4.1)	C09	45,020,379	152	5.00E-152	96.70%	289	BnaC09g43930D		At_PBS1 ortholog	lost	
676	*At_ADR1 (NL)*	Brassica napus (v4.1)	C09	48,466,297	275	2.00E-135	71.20%	394	BnaC09g50760D	NL	At_ADR1 ortholog	same	
677	*At_NDR1 (TM)*	Brassica napus (v4.1)	Cnn_random	9,952,559	218	2.00E-101	75.20%	330	BnaCnng10770D		At_NDR1 ortholog	lost	
678	*Bna_MAPk (Other-RLK)*	Brassica napus (v4.1)	Cnn_random	16,613,600	69	3.00E-68	100%	114	BnaCnng17720D		Bna_MAPk gene		
679	*Bna_MAPk (Other-RLK)*	Brassica napus (v4.1)	Cnn_random	16,614,919	51	1.00E-114	98%	115	BnaCnng17720D		Bna_MAPk gene		
680	*Bna_MAPk (Other-RLK)*	Brassica napus (v4.1)	Cnn_random	16,615,782	138	1.00E-114	88.40%	216	BnaCnng17720D		Bna_MAPk gene		
681	*At_SOBIR1 (LRR-RLK)*	Brassica napus (v4.1)	Cnn_random	38,131,070	644	0	73.70%	838	BnaCnng39490D	LRR-RLK	At_SOBIR1 ortholog	same	
682	*At_SOBIR1 (LRR-RLK)*	Brassica napus (v4.1)	Cnn_random	38,137,776	609	0	77.60%	909	BnaCnng39500D	LRR-RLK	At_SOBIR1 ortholog	same	
683	*At_RPM1 (NL)*	Brassica napus (v4.1)	Cnn_random	51,653,077	155	1.00E-52	71.60%	208	BnaCnng52210D		At_RPM1 ortholog	lost	
684	*Bna_LepR3/Rlm2 (LRR-RLP)*	Brassica napus (v4.1)	Cnn_random	65,300,836	729	0	86.40%	1082	BnaCnng65590D	LRR-RLP	Bna_LepR3/Rlm2 paralog (segme	same	
685	*Bra_Crr1a (TNL)*	Brassica napus (v4.1)	Cnn_random	77,044,835	372	0	79.80%	562	BnaCnng76010D	NL	Bra_Crr1a ortholog	different	
686	*At_NRG1a (RNL)*	Brassica napus (v4.1)	Unn_random	5,766,262	167	4.00E-65	74.20%	244	BnaUnng04020D	LRR-RLP	At_NRG1a ortholog	different	
687	*At_NRG1b (RNL)*	Brassica napus (v4.1)	Unn_random	5,766,268	169	9.00E-73	75.70%	268	BnaUnng04020D	LRR-RLP	At_NRG1b ortholog	different	
688	*Bna_LepR3/Rlm2 (LRR-RLP)*	Brassica nigra (v2)	B01	12,952,287	169	4.00E-62	71%	235	BniB041244-TA		Bna_LepR3/Rlm2 ortholog	lost	
689	*At_NDR1 (TM)*	Brassica nigra (v2)	B01	14,956,123	219	4.00E-105	79.40%	340	BniB020860-TA		At_NDR1 ortholog	lost	
690	*Bna_Rlm9/4/7 (Other-RLK)*	Brassica nigra (v2)	B01	17,398,868	414	5.00E-165	74.80%	538	BniB024659-TA	Other-RLK	Bna_Rlm9/4/7 ortholog	same	
691	*At_NRG1a (RNL)*	Brassica nigra (v2)	B01	21,138,263	230	9.00E-74	70.80%	270	BniB045931-TA	CNL	At_NRG1a ortholog	different	
692	*At_NRG1a (RNL)*	Brassica nigra (v2)	B01	21,140,184	378	2.00E-151	74.60%	499	BniB045931-TA	CNL	At_NRG1a ortholog	different	
693	*At_NRG1b (RNL)*	Brassica nigra (v2)	B01	21,140,184	378	2.00E-152	73.20%	502	BniB045931-TA	CNL	At_NRG1b ortholog	different	
694	*At_NRG1b (RNL)*	Brassica nigra (v2)	B01	22,315,847	281	0	71.80%	391	BniB044496-TA	CNL	At_NRG1b ortholog	different	
695	*At_NRG1a (RNL)*	Brassica nigra (v2)	B01	22,315,853	279	0	74.50%	389	BniB044496-TA	CNL	At_NRG1a ortholog	different	
696	*At_NDR1 (TM)*	Brassica nigra (v2)	B01	24,972,690	218	3.00E-101	75.60%	329	BniB035387-TA		At_NDR1 ortholog	lost	

697	*At_RFO3 (Other-RLK)*	Brassica nigra (v2)	B01	26,727,042	186	2.00E-107	74.70%	253	BniB035242-TA	Other-RLK	At_RFO3 ortholog	same	
698	*At_RFO3 (Other-RLK)*	Brassica nigra (v2)	B01	26,728,164	162	2.00E-57	76.50%	220	BniB035242-TA	Other-RLK	At_RFO3 ortholog	same	
699	*At_RPM1 (NL)*	Brassica nigra (v2)	B01	29,227,777	945	0	81.40%	1479	BniB003381-TA	NL	At_RPM1 ortholog	same	
700	*At_PBS1 (STK)*	Brassica nigra (v2)	B02	31,350,843	152	3.00E-148	91.40%	265	BniB023846-TA		At_PBS1 ortholog	lost	
701	*At_RPS2 (NL)*	Brassica nigra (v2)	B02	38,730,136	979	0	81.20%	1471	BniB013353-TA	CNL	At_RPS2 ortholog	different	
702	*At_BAK1 (LRR-RLK)*	Brassica nigra (v2)	B02	41,971,113	269	0	83.20%	434	BniB009226-TA	LRR-RLK	At_BAK1 ortholog	same	1
703	*At_BAK1 (LRR-RLK)*	Brassica nigra (v2)	B02	41,971,747	184	0	84.70%	264	BniB009226-TA	LRR-RLK	At_BAK1 ortholog	same	1
704	*At_BAK1 (LRR-RLK)*	Brassica nigra (v2)	B02	42,104,490	184	0	84.70%	264	BniB014037-TA	LRR-RLK	At_BAK1 ortholog	same	1
705	*At_BAK1 (LRR-RLK)*	Brassica nigra (v2)	B02	42,105,124	269	0	83.20%	434	BniB014037-TA	LRR-RLK	At_BAK1 ortholog	same	1
706	*Bna_MAPk (Other-RLK)*	Brassica nigra (v2)	B03	27,880,831	213	7.00E-155	70.80%	278	BniB032687-TA		Bna_MAPk ortholog	lost	
707	*At_SOBIR1 (LRR-RLK)*	Brassica nigra (v2)	B03	34,330,370	641	0	82.60%	1015	BniB011337-TA	LRR-RLK	At_SOBIR1 ortholog	same	
708	*Bra_cRa/cRb (TNL)*	Brassica nigra (v2)	B03	37,980,890	149	3.00E-67	78.50%	255	BniB030941-TA	TNL	Bra_cRa/cRb ortholog	same	
709	*Bra_Crr1a (TNL)*	Brassica nigra (v2)	B03	37,981,704	290	0	72.70%	414	BniB030941-TA	TNL	Bra_Crr1a ortholog	same	
710	*Bna_Rlm9/4/7 (Other-RLK)*	Brassica nigra (v2)	B03	38,217,503	418	6.00E-158	73.40%	518	BniB030989-TA	Other-RLK	Bna_Rlm9/4/7 ortholog	same	2
711	*Bna_Rlm9/4/7 (Other-RLK)*	Brassica nigra (v2)	B03	38,341,281	422	2.00E-157	72.50%	516	BniB022022-TA		Bna_Rlm9/4/7 ortholog	lost	2
712	*At_PBS1 (STK)*	Brassica nigra (v2)	B03	42,087,802	161	2.00E-66	79.50%	238	BniB031841-TA		At_PBS1 ortholog	lost	
713	*Bra_cRa/cRb (TNL)*	Brassica nigra (v2)	B03	42,439,880	148	4.00E-62	76%	238	BniB035654-TA	TX	Bra_cRa/cRb ortholog	different	
714	*At_NRG1a (RNL)*	Brassica nigra (v2)	B04	9,292,857	288	2.00E-101	70.40%	352	BniB004370-TA	CNL	At_NRG1a ortholog	different	
715	*At_NRG1b (RNL)*	Brassica nigra (v2)	B04	9,292,875	279	3.00E-106	71.60%	367	BniB004370-TA	CNL	At_NRG1b ortholog	different	
716	*At_NRG1a (RNL)*	Brassica nigra (v2)	B04	11,525,894	154	2.00E-90	79.20%	233	BniB047087-TA		At_NRG1a ortholog	lost	
717	*At_NRG1b (RNL)*	Brassica nigra (v2)	B04	11,525,894	154	1.00E-89	76.60%	219	BniB047087-TA		At_NRG1b ortholog	lost	
718	*Bna_Rlm9/4/7 (Other-RLK)*	Brassica nigra (v2)	B04	14,701,293	359	2.00E-148	77.70%	490	BniB043159-TA	Other-RLK	Bna_Rlm9/4/7 ortholog	same	
719	*At_RLP1 (LRR-RLP)*	Brassica nigra (v2)	B04	19,368,160	489	0	80.70%	700	BniB035836-TA		At_RLP1 ortholog	lost	3
720	*At_RLP1 (LRR-RLP)*	Brassica nigra (v2)	B04	19,376,776	170	2.00E-66	74.10%	250	BniB035835-TA	LRR-RLP	At_RLP1 ortholog	same	3
721	*At_RLP1 (LRR-RLP)*	Brassica nigra (v2)	B04	19,378,229	489	0	80.90%	701	BniB035835-TA	LRR-RLP	At_RLP1 ortholog	same	3
722	*Bna_Rlm9/4/7 (Other-RLK)*	Brassica nigra (v2)	B04	23,576,435	414	0	91.30%	660	BniB022842-TA	Other-RLK	Bna_Rlm9/4/7 ortholog	same	4
723	*Bna_Rlm9/4/7 (Other-RLK)*	Brassica nigra (v2)	B04	23,579,207	344	5.00E-138	72.90%	459	BniB022842-TA	Other-RLK	Bna_Rlm9/4/7 ortholog	same	4
724	*Bna_Rlm9/4/7 (Other-RLK)*	Brassica nigra (v2)	B04	23,583,779	414	0	88.40%	632	BniB022841-TA	Other-RLK	Bna_Rlm9/4/7 ortholog	same	4
725	*Bna_Rlm9/4/7 (Other-RLK)*	Brassica nigra (v2)	B04	23,596,745	416	0	86.20%	617	BniB022840-TA	Other-RLK	Bna_Rlm9/4/7 ortholog	same	4
726	*Bna_Rlm9/4/7 (Other-RLK)*	Brassica nigra (v2)	B04	23,600,553	325	1.00E-139	74.40%	464	BniB022840-TA	Other-RLK	Bna_Rlm9/4/7 ortholog	same	4
727	*Bna_Rlm9/4/7 (Other-RLK)*	Brassica nigra (v2)	B04	32,540,444	425	2.00E-179	79%	580	BniB040191-TA	Other-RLK	Bna_Rlm9/4/7 ortholog	same	5
728	*Bna_Rlm9/4/7 (Other-RLK)*	Brassica nigra (v2)	B04	32,550,442	425	0	79.50%	588	BniB040189-TA	Other-RLK	Bna_Rlm9/4/7 ortholog	same	5
729	*At_RFO3 (Other-RLK)*	Brassica nigra (v2)	B05	5,338,428	176	2.00E-168	79.50%	285	BniB008900-TA	Other-RLK	At_RFO3 ortholog	same	
730	*At_RFO3 (Other-RLK)*	Brassica nigra (v2)	B05	5,339,032	184	2.00E-168	77.70%	263	BniB008900-TA	Other-RLK	At_RFO3 ortholog	same	
731	*Bna_MAPk (Other-RLK)*	Brassica nigra (v2)	B05	6,598,310	343	2.00E-136	72.80%	447	BniB039261-TA		Bna_MAPk ortholog	lost	
732	*At_NDR1 (TM)*	Brassica nigra (v2)	B05	8,603,809	217	2.00E-90	73.70%	298	BniB043481-TA		At_NDR1 ortholog	lost	
733	*At_WRR12 (TNL)*	Brassica nigra (v2)	B05	14,948,097	156	9.00E-55	71.10%	216	BniB041320-TA	TX	At_WRR12 ortholog	different	
734	*At_RLP32 (LRR-RLP)*	Brassica nigra (v2)	B05	24,268,644	439	1.00E-148	70.30%	493	BniB033518-TA	LRR-RLP	At_RLP32 ortholog	same	
735	*At_RLP32 (LRR-RLP)*	Brassica nigra (v2)	B05	26,163,534	593	0	75%	716	BniB038823-TA	LRR-RLP	At_RLP32 ortholog	same	
736	*At_SOBIR1 (LRR-RLK)*	Brassica nigra (v2)	B05	29,948,657	466	0	80.40%	717	BniB020100-TA	LRR-RLK	At_SOBIR1 ortholog	same	
737	*At_RPP13 (CNL)*	Brassica nigra (v2)	B05	36,124,265	256	4.00E-177	78.10%	404	BniB004987-TA	CN	At_RPP13 ortholog	different	
738	*Bna_Rlm9/4/7 (Other-RLK)*	Brassica nigra (v2)	B06	19,890,208	420	4.00E-154	71.40%	507	BniB009878-TA		Bna_Rlm9/4/7 ortholog	lost	
739	*At_RLM1b (TNL)*	Brassica nigra (v2)	B06	20,499,833	369	2.00E-156	71.20%	526	BniB026501-TA	NL	At_RLM1b ortholog	different	
740	*At_RLM1b (TNL)*	Brassica nigra (v2)	B06	21,403,196	376	0	70.70%	537	BniB011078-TA	TNL	At_RLM1b ortholog	same	6
741	*At_RLM1b (TNL)*	Brassica nigra (v2)	B06	21,409,085	386	0	70.70%	555	BniB011079-TA	TNL	At_RLM1b ortholog	same	6
742	*At_RPS4 (TNL)*	Brassica nigra (v2)	B06	25,370,149	424	0	78.30%	650	BniB029398-TA	TNL	At_RPS4 ortholog	same	
743	*At_FLS2 (LRR-RLK)*	Brassica nigra (v2)	B06	25,743,087	1031	0	80.60%	1536	BniB029419-TA	LRR-RLK	At_FLS2 ortholog	same	
744	*Bna_MAPk (Other-RLK)*	Brassica nigra (v2)	B06	26,281,408	149	2.00E-55	77.10%	209	BniB029272-TA	Other-RLK	Bna_MAPk ortholog	same	
745	*At_RPS4 (TNL)*	Brassica nigra (v2)	B06	27,521,969	306	0	80.70%	479	BniB028749-TA	TNL	At_RPS4 ortholog	same	7
746	*At_RRS1 (TNL)*	Brassica nigra (v2)	B06	27,526,048	359	3.00E-152	72.10%	517	BniB028832-TA	NL	At_RRS1 ortholog	different	7
747	*At_FLS2 (LRR-RLK)*	Brassica nigra (v2)	B06	27,944,102	256	4.00E-126	74.20%	368	BniB028864-TA		At_FLS2 ortholog	lost	
748	*At_FLS2 (LRR-RLK)*	Brassica nigra (v2)	B06	27,945,048	457	0	77.80%	627	BniB028864-TA		At_FLS2 ortholog	lost	
749	*At_FLS2 (LRR-RLK)*	Brassica nigra (v2)	B06	27,946,408	254	0	78.70%	341	BniB028865-TA		At_FLS2 ortholog	lost	
750	*Bol_FocBo1 (TNL)*	Brassica nigra (v2)	B06	30,300,581	150	7.00E-171	70.60%	209	BniB027312-TA	TNL	Bol_FocBo1 ortholog	same	8
751	*Bol_FocBo1 (TNL)*	Brassica nigra (v2)	B06	30,330,370	148	9.00E-139	72.90%	219	BniB027023-TA	TX	Bol_FocBo1 ortholog	different	8
752	*At_RFO1 (Other-RLK)*	Brassica nigra (v2)	B07	21,674,898	419	0	71.80%	480	BniB005414-TA	Other-RLK	At_RFO1 ortholog	same	
753	*Bna_MAPk (Other-RLK)*	Brassica nigra (v2)	B07	24,763,588	202	4.00E-89	76.20%	300	BniB042716-TA		Bna_MAPk ortholog	lost	
754	*At_BAK1 (LRR-RLK)*	Brassica nigra (v2)	B07	25,442,767	312	9.00E-172	80.40%	509	BniB042663-TA	LRR-RLK	At_BAK1 ortholog	same	
755	*Bna_Rlm9/4/7 (Other-RLK)*	Brassica nigra (v2)	B07	28,608,839	357	3.00E-137	71.10%	457	BniB008083-TA	Other-RLK	Bna_Rlm9/4/7 ortholog	same	
756	*Bna_Rlm9/4/7 (Other-RLK)*	Brassica nigra (v2)	B07	30,089,132	259	1.00E-118	71.80%	330	BniB025882-TA	Other-RLK	Bna_Rlm9/4/7 ortholog	same	9
757	*At_WRR12 (TNL)*	Brassica nigra (v2)	B07	30,157,430	409	2.00E-145	76%	504	BniB025877-TA	Other-NLR	At_WRR12 ortholog	different	9
758	*At_WRR12 (TNL)*	Brassica nigra (v2)	B07	30,159,498	149	3.00E-68	85.90%	259	BniB025957-TA	TX	At_WRR12 ortholog	different	9
759	*At_WRR12 (TNL)*	Brassica nigra (v2)	B07	30,161,468	510	0	79.60%	821	BniB025958-TA	TNL	At_WRR12 ortholog	same	9
760	*At_WRR12 (TNL)*	Brassica nigra (v2)	B07	30,163,081	370	0	83.50%	551	BniB025958-TA	TNL	At_WRR12 ortholog	same	9
761	*Bna_Rlm9/4/7 (Other-RLK)*	Brassica nigra (v2)	B07	30,571,723	244	7.00E-89	70.90%	315	BniB025991-TA	Other-RLK	Bna_Rlm9/4/7 ortholog	same	10
762	*Bna_Rlm9/4/7 (Other-RLK)*	Brassica nigra (v2)	B07	30,595,982	353	5.00E-132	70.80%	442	BniB025994-TA	Other-RLK	Bna_Rlm9/4/7 ortholog	same	10
763	*Bna_Rlm9/4/7 (Other-RLK)*	Brassica nigra (v2)	B07	30,603,324	214	2.00E-108	71%	271	BniB025835-TA	Other-RLK	Bna_Rlm9/4/7 ortholog	same	10
764	*Bna_Rlm9/4/7 (Other-RLK)*	Brassica nigra (v2)	B07	30,625,114	298	2.00E-119	72.40%	405	BniB025833-TA	Other-RLK	Bna_Rlm9/4/7 ortholog	same	10
765	*At_RFO3 (Other-RLK)*	Brassica nigra (v2)	B07	31,515,905	192	8.00E-95	75.50%	284	BniB039398-TA	Other-RLK	At_RFO3 ortholog	same	
766	*At_RFO3 (Other-RLK)*	Brassica nigra (v2)	B07	31,516,524	190	8.00E-70	74.20%	259	BniB039398-TA	Other-RLK	At_RFO3 ortholog	same	
767	*At_RPP2a (TNL)*	Brassica nigra (v2)	B07	32,354,464	633	0	70.30%	835	BniB039473-TA	TNL	At_RPP2a ortholog	same	
768	*Bra_Crr1a (TNL)*	Brassica nigra (v2)	B07	32,937,342	507	0	80.20%	769	BniB039103-TA	TNL	Bra_Crr1a ortholog	same	11
769	*Bra_Crr1a (TNL)*	Brassica nigra (v2)	B07	32,940,527	233	8.00E-90	77.20%	327	BniB039102-TA	TX	Bra_Crr1a ortholog	different	11
770	*Bra_Crr1a (TNL)*	Brassica nigra (v2)	B07	32,960,722	509	0	78.70%	759	BniB039101-TA	TNL	Bra_Crr1a ortholog	same	11
771	*Bna_Rlm9/4/7 (Other-RLK)*	Brassica nigra (v2)	B07	38,911,891	399	1.00E-131	70.10%	441	BniB015276-TA		Bna_Rlm9/4/7 ortholog	lost	
772	*Bna_MAPk (Other-RLK)*	Brassica nigra (v2)	B07	39,201,651	219	4.00E-158	75.70%	312	BniB015308-TA		Bna_MAPk ortholog	lost	
773	*At_RFO2 (LRR-RLP)*	Brassica nigra (v2)	B07	39,390,390	721	0	78.50%	1028	BniB015331-TA	LRR-RLP	At_RFO2 ortholog	same	
774	*At_ADR1 (NL)*	Brassica nigra (v2)	B07	39,830,124	245	3.00E-121	71.80%	340	BniB015480-TA	CNL	At_ADR1 ortholog	different	
775	*At_RPP8 (CNL)*	Brassica nigra (v2)	B07	40,664,104	293	4.00E-102	74%	357	BniB044790-TA	NL	At_RPP8 ortholog	different	
776	*At_NRG1b (RNL)*	Brassica nigra (v2)	B08	4,364,893	268	1.00E-100	70.50%	350	BniB040020-TA	CNL	At_NRG1b ortholog	different	
777	*At_NRG1a (RNL)*	Brassica nigra (v2)	B08	4,364,893	263	2.00E-95	70.30%	335	BniB040020-TA	CNL	At_NRG1a ortholog	different	
778	*At_RPP13 (CNL)*	Brassica nigra (v2)	B08	24,972,437	184	0	75%	202	BniB033688-TA		At_RPP13 ortholog	lost	
779	*At_PBS1 (STK)*	Brassica nigra (v2)	B08	26,909,391	152	6.00E-154	98%	286	BniB013913-TA		At_PBS1 ortholog	lost	
780	*Bna_LepR3/Rlm2 (LRR-RLP)*	Brassica nigra (v2)	B08	27,254,855	405	2.00E-170	79.50%	560	BniB028797-TA	LRR-RLP	Bna_LepR3/Rlm2 ortholog	same	
781	*Bna_LepR3/Rlm2 (LRR-RLP)*	Brassica nigra (v2)	B08	27,256,539	579	0	77.50%	775	BniB028797-TA	LRR-RLP	Bna_LepR3/Rlm2 ortholog	same	
782	*Bju_WRR1 (CNL)*	Brassica nigra (v2)	B08	38,507,937	317	1.00E-147	74.10%	472	BniB000049-TA	CNL	Bju_WRR1 ortholog	same	
783	*At_RPP2a (TNL)*	Brassica nigra (v2)	B08	39,243,312	356	0	72.40%	537	BniB000958-TA	TNL	At_RPP2a ortholog	same	12
784	*Bra_cRa/cRb (TNL)*	Brassica nigra (v2)	B08	43,101,622	374	2.00E-174	76.70%	585	BniB000558-TA	Other-NLR	Bra_cRa/cRb ortholog	different	12
785	*Bra_cRa/cRb (TNL)*	Brassica nigra (v2)	B08	43,101,622	374	2.00E-174	76.70%	585	BniB000558-TA	Other-NLR	Bra_cRa/cRb ortholog	different	12
786	*Bra_cRa/cRb (TNL)*	Brassica nigra (v2)	B08	43,102,347	149	3.00E-78	88.50%	290	BniB000558-TA	Other-NLR	Bra_cRa/cRb ortholog	different	12
787	*Bra_cRa/cRb (TNL)*	Brassica nigra (v2)	B08	43,111,723	220	2.00E-80	74.00%	297	BniB000556-TA	TN	Bra_cRa/cRb ortholog	different	12
788	*Bna_Rlm9/4/7 (Other-RLK)*	Brassica nigra (v2)	scaffold_101	850,272	301	6.00E-100	70.70%	348	BniB002366-TA	Other-RLK	Bna_Rlm9/4/7 ortholog	same	
789	*At_BAK1 (LRR-RLK)*	Brassica oleracea (v2.1)	C01	2,843,995	271	0	89.20%	499	Bo1g008160.1	LRR-RLK	At_BAK1 ortholog	same	
790	*At_RPP2a (TNL)*	Brassica oleracea (v2.1)	C01	7,741,199	466	0	74.40%	688	Bo1g021620.1	TNL	At_RPP2a ortholog	same	
791	*At_RPP2b (TNL)*	Brassica oleracea (v2.1)	C01	7,745,955	352	0	70.10%	405	Bo1g021620.1	TNL	At_RPP2b ortholog	same	
792	*At_RPP2b (TNL)*	Brassica oleracea (v2.1)	C01	7,747,081	216	2.00E-95	72.60%	344	Bo1g021620.1	TNL	At_RPP2b ortholog	same	
793	*At_RPS2 (NL)*	Brassica oleracea (v2.1)	C01	13,003,644	929	0	83.40%	1483	Bo1g041880.1	CNL	At_RPS2 ortholog	different	
794	*At_RPP13 (CNL)*	Brassica oleracea (v2.1)	C01	20,806,285	180	0	71.10%	206	Bo1g070920.1	CNL	At_RPP13 ortholog	same	
795	*At_NDR1 (TM)*	Brassica oleracea (v2.1)	C01	33,803,504	218	1.00E-101	75.20%	330	Bo1g108800.1		At_NDR1 ortholog	lost	
796	*At_RFO3 (Other-RLK)*	Brassica oleracea (v2.1)	C01	37,127,207	161	3.00E-106	77.60%	253	Bo1g124810.1		At_RFO3 ortholog	lost	
797	*At_RFO3 (Other-RLK)*	Brassica oleracea (v2.1)	C01	37,127,906	163	4.00E-58	76%	223	Bo1g124810.1		At_RFO3 ortholog	lost	
798	*Bna_MAPk (Other-RLK)*	Brassica oleracea (v2.1)	C01	38,152,217	210	3.00E-150	71.90%	293	Bo1g130040.1		Bna_MAPk ortholog	lost	
799	*At_RPM1 (NL)*	Brassica oleracea (v2.1)	C01	41,767,323	938	0	81.40%	1435	Bo1g147600.1	NL	At_RPM1 ortholog	same	
800	*At_PBS1 (STK)*	Brassica oleracea (v2.1)	C02	2,869,635	317	8.00E-164	83.90%	521	Bo2g011100.1		At_PBS1 ortholog	lost	
801	*At_FLS2 (LRR-RLK)*	Brassica oleracea (v2.1)	C02	40,189,220	328	8.00E-133	72.20%	457	Bo2g129990.1	LRR-RLK	At_FLS2 ortholog	same	
802	*At_FLS2 (LRR-RLK)*	Brassica oleracea (v2.1)	C02	40,190,169	714	0	77.30%	974	Bo2g129990.1	LRR-RLK	At_FLS2 ortholog	same	
803	*At_WRR8 (TNL)*	Brassica oleracea (v2.1)	C02	40,781,793	180	8.00E-140	73.30%	273	Bo2g131620.1	NL	At_WRR8 ortholog	different	
804	*At_NRG1a (RNL)*	Brassica oleracea (v2.1)	C02	48,538,910	242	2.00E-97	75.20%	341	Bo2g155530.1		At_NRG1a ortholog	lost	
805	*At_NRG1b (RNL)*	Brassica oleracea (v2.1)	C02	48,538,916	244	1.00E-101	74.10%	353	Bo2g155530.1		At_NRG1b ortholog	lost	
806	*At_PBS1 (STK)*	Brassica oleracea (v2.1)	C03	2,984,259	160	3.00E-67	78.10%	241	Bo3g009020.1		At_PBS1 ortholog	lost	
807	*At_PBS1 (STK)*	Brassica oleracea (v2.1)	C03	2,984,942	242	8.00E-92	71%	312	Bo3g009020.1		At_PBS1 ortholog	lost	
808	*Bra_cRa/cRb (TNL)*	Brassica oleracea (v2.1)	C03	6,145,397	148	4.00E-66	79%	251	Bo3g018390.1	TNL	Bra_cRa/cRb ortholog	same	
809	*At_SOBIR1 (LRR-RLK)*	Brassica oleracea (v2.1)	C03	9,708,201	613	0	78.10%	950	Bo3g026470.1	LRR-RLK	At_SOBIR1 ortholog	same	
810	*At_NDR1 (TM)*	Brassica oleracea (v2.1)	C03	28,858,163	220	2.00E-100	78.10%	327	Bo3g076120.1		At_NDR1 ortholog	lost	
811	*Bna_LepR3/Rlm2 (LRR-RLP)*	Brassica oleracea (v2.1)	C03	29,520,968	180	0	71.10%	235	Bo3g080000.1	LRR-RLP	Bna_LepR3/Rlm2 ortholog	same	
812	*At_RFO3 (Other-RLK)*	Brassica oleracea (v2.1)	C03	30,016,398	292	2.00E-169	74.30%	400	Bo3g081630.1	Other-RLK	At_RFO3 ortholog	same	
813	*At_RFO3 (Other-RLK)*	Brassica oleracea (v2.1)	C03	30,018,692	235	2.00E-86	72.70%	311	Bo3g081630.1	Other-RLK	At_RFO3 ortholog	same	

814	At_RPP2a (TNL)	Brassica oleracea (v2.1)	C03	56,738,441	277	1.00E-106	71.80%	383	Bo3g158750.1	NBS	At_RPP2a ortholog	different	
815	Bra_Crr1a (TNL)	Brassica oleracea (v2.1)	C03	57,779,818	372	0	90.50%	661	Bo3g164020.1	CNL	Bra_Crr1a ortholog	different	1
816	Bra_cRa/cRb (TNL)	Brassica oleracea (v2.1)	C03	57,786,157	149	3.00E-62	74.40%	239	Bo3g164030.1	TX	Bra_cRa/cRb ortholog	different	1
817	Bra_Crr1a (TNL)	Brassica oleracea (v2.1)	C03	57,786,388	226	1.00E-92	78.30%	336	Bo3g164030.1	TX	Bra_Crr1a ortholog	different	1
818	Bra_Crr1a (TNL)	Brassica oleracea (v2.1)	C03	57,798,947	372	0	83%	596	Bo3g164040.1	TNL	Bra_Crr1a ortholog	same	1
819	At_BAK1 (LRR-RLK)	Brassica oleracea (v2.1)	C03	60,157,587	268	2.00E-120	76.40%	402	Bo3g170710.1	LRR-RLK	At_BAK1 ortholog	same	
820	At_RPP8 (CNL)	Brassica oleracea (v2.1)	C04	7,439,949	233	2.00E-73	71.60%	271	Bo4g034660.1	CN	At_RPP8 ortholog	different	2
821	Bju_WRR1 (CNL)	Brassica oleracea (v2.1)	C04	7,439,949	230	1.00E-116	87.80%	417	Bo4g034660.1	CN	Bju_WRR1 ortholog	different	2
822	Bju_WRR1 (CNL)	Brassica oleracea (v2.1)	C04	7,449,650	315	0	89.20%	566	Bo4g034680.1	CNL	Bju_WRR1 ortholog	same	2
823	Bju_WRR1 (CNL)	Brassica oleracea (v2.1)	C04	7,451,456	377	0	81.10%	574	Bo4g034680.1	CNL	Bju_WRR1 ortholog	same	2
824	Bju_WRR1 (CNL)	Brassica oleracea (v2.1)	C04	7,452,613	199	0	74.80%	270	Bo4g034680.1	CNL	Bju_WRR1 ortholog	same	2
825	Bju_WRR1 (CNL)	Brassica oleracea (v2.1)	C04	7,461,644	156	1.00E-58	75.60%	229	Bo4g034710.1	NL	Bju_WRR1 ortholog	different	2
826	Bju_WRR1 (CNL)	Brassica oleracea (v2.1)	C04	7,462,787	367	1.00E-130	70.50%	462	Bo4g034710.1	NL	Bju_WRR1 ortholog	different	2
827	Bju_WRR1 (CNL)	Brassica oleracea (v2.1)	C04	7,464,180	163	3.00E-122	82.20%	239	Bo4g034720.1		Bju_WRR1 ortholog	lost	2
828	Bju_WRR1 (CNL)	Brassica oleracea (v2.1)	C04	7,464,670	151	3.00E-122	79.40%	224	Bo4g034730.1		Bju_WRR1 ortholog	lost	2
829	Bju_WRR1 (CNL)	Brassica oleracea (v2.1)	C04	7,466,105	361	2.00E-159	83.10%	551	Bo4g034740.1		Bju_WRR1 ortholog	lost	2
830	Bju_WRR1 (CNL)	Brassica oleracea (v2.1)	C04	7,467,193	168	6.00E-121	79.70%	235	Bo4g034740.1		Bju_WRR1 ortholog	lost	2
831	Bju_WRR1 (CNL)	Brassica oleracea (v2.1)	C04	7,467,698	151	6.00E-121	78.80%	224	Bo4g034750.1		Bju_WRR1 ortholog	lost	2
832	At_RLP23 (LRR-RLP)	Brassica oleracea (v2.1)	C04	46,966,124	357	2.00E-148	71.40%	494	Bo4g177600.1		At_RLP23 ortholog	lost	
833	At_RLP42 (LRR-RLP)	Brassica oleracea (v2.1)	C04	46,966,124	345	1.00E-114	70.10%	394	Bo4g177600.1		At_RLP42 ortholog	lost	
834	At_SOBIR1 (LRR-RLK)	Brassica oleracea (v2.1)	C04	47,021,854	644	0	73.10%	856	Bo4g177660.1	LRR-RLK	At_SOBIR1 ortholog	same	3
835	At_SOBIR1 (LRR-RLK)	Brassica oleracea (v2.1)	C04	47,028,585	609	0	77.80%	907	Bo4g177670.1	LRR-RLK	At_SOBIR1 ortholog	same	3
836	Bna_MAPk (Other-RLK)	Brassica oleracea (v2.1)	C05	8,651,182	155	1.00E-156	81.90%	232	Bo5g025110.1		Bna_MAPk ortholog	lost	
837	Bna_Rlm9/4/7 (Other-RLK)	Brassica oleracea (v2.1)	C05	9,578,599	203	7.00E-71	74.30%	261	Bo5g027440.1		Bna_Rlm9/4/7 ortholog	lost	
838	At_RLP23 (LRR-RLP)	Brassica oleracea (v2.1)	C05	13,435,429	307	0	74.90%	450	Bo5g041170.1		At_RLP23 ortholog	lost	
839	At_RLP42 (LRR-RLP)	Brassica oleracea (v2.1)	C05	13,435,429	303	0	75.50%	372	Bo5g041170.1		At_RLP42 ortholog	lost	
840	Bna_MAPk (Other-RLK)	Brassica oleracea (v2.1)	C05	36,850,907	255	9.00E-122	74.90%	346	Bo5g121280.1		Bna_MAPk ortholog	lost	
841	At_RFO3 (Other-RLK)	Brassica oleracea (v2.1)	C05	38,900,343	238	3.00E-107	73.10%	343	Bo5g126490.1	Other-RLK	At_RFO3 ortholog	same	
842	Bna_Rlm9/4/7 (Other-RLK)	Brassica oleracea (v2.1)	C06	24,760,290	427	0	80.50%	643	Bo6g080830.1	Other-RLK	Bna_Rlm9/4/7 ortholog	same	4
843	Bna_Rlm9/4/7 (Other-RLK)	Brassica oleracea (v2.1)	C06	24,761,687	414	0	94.60%	698	Bo6g080830.1	Other-RLK	Bna_Rlm9/4/7 ortholog	same	4
844	Bna_Rlm9/4/7 (Other-RLK)	Brassica oleracea (v2.1)	C06	24,769,386	322	8.00E-137	71.70%	456	Bo6g080850.1	Other-RLK	Bna_Rlm9/4/7 ortholog	same	4
845	Bna_Rlm9/4/7 (Other-RLK)	Brassica oleracea (v2.1)	C06	24,772,598	415	0	95.40%	698	Bo6g080850.1	Other-RLK	Bna_Rlm9/4/7 ortholog	same	4
846	At_BAK1 (LRR-RLK)	Brassica oleracea (v2.1)	C06	28,705,263	247	9.00E-170	96.70%	504	Bo6g093010.1	LRR-RLK	At_BAK1 ortholog	same	
847	At_BAK1 (LRR-RLK)	Brassica oleracea (v2.1)	C06	35,744,853	154	1.00E-140	80.50%	251	Bo6g113320.1	LRR-RLK	At_BAK1 ortholog	same	
848	At_BAK1 (LRR-RLK)	Brassica oleracea (v2.1)	C07	8,989,105	157	1.00E-123	71.30%	230	Bo7g025340.1	LRR-RLK	At_BAK1 ortholog	same	
849	Bju_WRR1 (CNL)	Brassica oleracea (v2.1)	C07	34,712,360	317	2.00E-147	73.80%	463	Bo7g089600.1	CN	Bju_WRR1 ortholog	different	
850	Bol_FocBo1 (TNL)	Brassica oleracea (v2.1)	C07	40,333,523	360	4.00E-167	71.30%	437	Bo7g104800.1	TNL	Bol_FocBo1 gene		
851	Bol_FocBo1 (TNL)	Brassica oleracea (v2.1)	C07	40,334,957	415	0	91.50%	773	Bo7g104800.1	TNL	Bol_FocBo1 gene		
852	Bol_FocBo1 (TNL)	Brassica oleracea (v2.1)	C07	40,335,682	164	2.00E-75	87.80%	281	Bo7g104800.1	TNL	Bol_FocBo1 gene		
853	Bol_FocBo1 (TNL)	Brassica oleracea (v2.1)	C07	40,337,119	310	0	83.20%	504	Bo7g104800.1	TNL	Bol_FocBo1 gene		
854	Bol_FocBo1 (TNL)	Brassica oleracea (v2.1)	C07	40,338,297	361	0	95.20%	616	Bo7g104800.1	TNL	Bol_FocBo1 gene		
855	Bra_cRa/cRb (TNL)	Brassica oleracea (v2.1)	C07	42,203,237	370	0	77.80%	598	Bo7g107710.1	TNL	Bra_cRa/cRb ortholog	same	5
856	Bra_cRa/cRb (TNL)	Brassica oleracea (v2.1)	C07	42,203,237	370	0	77.80%	597	Bo7g107710.1	TNL	Bra_cRa/cRb ortholog	same	5
857	Bra_cRa/cRb (TNL)	Brassica oleracea (v2.1)	C07	42,206,181	148	4.00E-71	84.40%	268	Bo7g107710.1	TNL	Bra_cRa/cRb ortholog	same	5
858	Bra_cRa/cRb (TNL)	Brassica oleracea (v2.1)	C07	42,220,445	306	0	73.50%	440	Bo7g107730.1	Other-NLR	Bra_cRa/cRb ortholog	different	5
859	Bra_cRa/cRb (TNL)	Brassica oleracea (v2.1)	C07	42,220,445	306	0.00E+00	73.50%	440	Bo7g107730.1	Other-NLR	Bra_cRa/cRb ortholog	different	5
860	Bra_cRa/cRb (TNL)	Brassica oleracea (v2.1)	C07	42,223,052	374	0	85.80%	670	Bo7g107730.1	Other-NLR	Bra_cRa/cRb ortholog	different	5
861	Bra_cRa/cRb (TNL)	Brassica oleracea (v2.1)	C07	42,223,052	374	0	85.80%	670	Bo7g107730.1	Other-NLR	Bra_cRa/cRb ortholog	different	5
862	Bra_cRa/cRb (TNL)	Brassica oleracea (v2.1)	C07	42,224,071	220	4.00E-82	75.40%	303	Bo7g107730.1	Other-NLR	Bra_cRa/cRb ortholog	different	5
863	Bra_cRa/cRb (TNL)	Brassica oleracea (v2.1)	C07	42,229,707	149	2.00E-70	83.20%	265	Bo7g107740.1	TNL	Bra_cRa/cRb ortholog	same	5
864	Bna_Rlm9/4/7 (Other-RLK)	Brassica oleracea (v2.1)	C07	45,749,846	356	1.00E-178	70.50%	421	Bo7g114950.1	Other-RLK	Bna_Rlm9/4/7 ortholog	same	
865	Bna_Rlm9/4/7 (Other-RLK)	Brassica oleracea (v2.1)	C08	845,120	308	1.00E-122	75%	415	Bo8g004320.1	Other-RLK	Bna_Rlm9/4/7 ortholog	same	
866	Bra_cRa/cRb (TNL)	Brassica oleracea (v2.1)	C08	16,520,951	148	7.00E-57	70.20%	222	Bo8g050260.1	TX	Bra_cRa/cRb ortholog	different	
867	At_RFO2 (LRR-RLP)	Brassica oleracea (v2.1)	C08	21,603,020	734	0	71.10%	937	Bo8g067030.1	LRR-RLP	At_RFO2 ortholog	same	
868	Bna_MAPk (Other-RLK)	Brassica oleracea (v2.1)	C08	36,659,117	201	3.00E-154	80%	311	Bo8g104510.1		Bna_MAPk ortholog	lost	6
869	At_WRR12 (TNL)	Brassica oleracea (v2.1)	C08	36,734,777	463	0	74.50%	594	Bo8g104700.1	TNL	At_WRR12 ortholog	same	6
870	At_WRR12 (TNL)	Brassica oleracea (v2.1)	C08	36,736,379	374	0	85.50%	662	Bo8g104700.1	TNL	At_WRR12 ortholog	same	6
871	At_WRR12 (TNL)	Brassica oleracea (v2.1)	C08	36,738,124	149	3.00E-69	86.50%	263	Bo8g104700.1	TNL	At_WRR12 ortholog	same	6
872	At_WRR12 (TNL)	Brassica oleracea (v2.1)	C08	36,739,761	411	1.00E-146	75.40%	508	Bo8g104710.1	Other-NLR	At_WRR12 ortholog	different	6
873	At_WRR12 (TNL)	Brassica oleracea (v2.1)	C08	36,745,003	273	2.00E-144	80.20%	371	Bo8g104730.1	NL	At_WRR12 ortholog	different	6
874	Bna_Rlm9/4/7 (Other-RLK)	Brassica oleracea (v2.1)	C08	37,238,450	340	5.00E-131	71.40%	439	Bo8g105650.1		Bna_Rlm9/4/7 ortholog	lost	
875	At_RLP1 (LRR-RLP)	Brassica oleracea (v2.1)	C08	40,187,715	368	0	74.10%	519	Bo8g114130.1	LRR-RLP	At_RLP1 ortholog	same	
876	At_RLP1 (LRR-RLP)	Brassica oleracea (v2.1)	C08	40,188,821	370	0	85.40%	555	Bo8g114130.1	LRR-RLP	At_RLP1 ortholog	same	
877	At_RLP1 (LRR-RLP)	Brassica oleracea (v2.1)	C08	40,191,065	144	2.00E-51	72.20%	203	Bo8g114130.1	LRR-RLP	At_RLP1 ortholog	same	
878	At_NRG1a (RNL)	Brassica oleracea (v2.1)	C09	6,399,438	285	1.00E-112	71.50%	386	Bo9g021540.1	NL	At_NRG1a ortholog	different	7
879	At_NRG1a (RNL)	Brassica oleracea (v2.1)	C09	6,400,780	279	1.00E-139	81.30%	421	Bo9g021540.1	NL	At_NRG1a ortholog	different	7
880	At_NRG1b (RNL)	Brassica oleracea (v2.1)	C09	6,400,780	279	1.00E-139	80.20%	426	Bo9g021540.1	NL	At_NRG1b ortholog	different	7
881	At_NRG1a (RNL)	Brassica oleracea (v2.1)	C09	6,409,796	217	3.00E-70	70.90%	259	Bo9g021570.1	CNL	At_NRG1a ortholog	different	7
882	At_NRG1a (RNL)	Brassica oleracea (v2.1)	C09	6,410,716	257	3.00E-139	80.10%	410	Bo9g021570.1	CNL	At_NRG1a ortholog	different	7
883	At_NRG1a (RNL)	Brassica oleracea (v2.1)	C09	6,412,021	278	1.00E-123	82%	418	Bo9g021570.1	CNL	At_NRG1a ortholog	different	7
884	At_NRG1b (RNL)	Brassica oleracea (v2.1)	C09	6,412,021	278	5.00E-126	80.90%	426	Bo9g021570.1	CNL	At_NRG1b ortholog	different	7
885	At_RLM1b (TNL)	Brassica oleracea (v2.1)	C09	10,393,761	157	6.00E-140	78.90%	269	Bo9g029450.1	TN	At_RLM1b ortholog	different	
886	At_RLM1b (TNL)	Brassica oleracea (v2.1)	C09	11,684,019	149	0	85.20%	269	Bo9g034110.1	TNL	At_RLM1b ortholog	same	
887	At_RLM1b (TNL)	Brassica oleracea (v2.1)	C09	11,684,555	367	0	72.70%	549	Bo9g034110.1	TNL	At_RLM1b ortholog	same	
888	At_RPS4 (TNL)	Brassica oleracea (v2.1)	C09	18,480,246	440	0	75.90%	652	Bo9g061260.1	TNL	At_RPS4 ortholog	same	
889	At_FLS2 (LRR-RLK)	Brassica oleracea (v2.1)	C09	19,287,466	1031	0	81%	1533	Bo9g064150.1	LRR-RLK	At_FLS2 ortholog	same	
890	Bju_WRR1 (CNL)	Brassica oleracea (v2.1)	C09	45,633,012	242	2.00E-55	80.90%	215	Bo9g153640.1		Bju_WRR1 ortholog	lost	
891	At_PBS1 (STK)	Brassica oleracea (v2.1)	C09	46,331,563	229	1.00E-150	76.40%	368	Bo9g154710.1		At_PBS1 ortholog	lost	
892	At_PBS1 (STK)	Brassica oleracea (v2.1)	C09	49,658,715	152	2.00E-150	96%	283	Bo9g168980.1		At_PBS1 ortholog	lost	
893	Bna_LepR3/Rlm2 (LRR-RLP)	Brassica oleracea (v2.1)	C09	50,124,866	404	0	85.10%	589	Bo9g169800.1	LRR-RLP	Bna_LepR3/Rlm2 ortholog	same	
894	Bna_LepR3/Rlm2 (LRR-RLP)	Brassica oleracea (v2.1)	C09	50,126,401	508	0	81.40%	721	Bo9g169800.1	LRR-RLP	Bna_LepR3/Rlm2 ortholog	same	
895	Bna_MAPk (Other-RLK)	Brassica oleracea (v2.1)	Scaffold00534	183,178	228	2.00E-94	71.90%	325	Bo00534s150.1		Bna_MAPk ortholog	lost	
896	At_RLM1b (TNL)	Brassica oleracea (v2.1)	Scaffold00787	59,374	373	3.00E-155	70.70%	523	Bo00787s050.1	NL	At_RLM1b ortholog	different	
897	At_RLM1b (TNL)	Brassica oleracea (v2.1)	Scaffold00787	79,514	149	3.00E-72	83.80%	270	Bo00787s070.1	TX	At_RLM1b ortholog	different	
898	At_RPP2b (TNL)	Brassica oleracea (v2.1)	Scaffold00889	44,320	216	9.00E-111	81%	391	Bo00889s060.1	TNL	At_RPP2b ortholog	same	
899	At_RPP2b (TNL)	Brassica oleracea (v2.1)	Scaffold01202	3,776	216	4.00E-109	80%	386	Bo01202s010.1		At_RPP2b ortholog	lost	
900	At_WRR12 (TNL)	Brassica oleracea (v2.1)	Scaffold03215	899	299	4.00E-176	85.60%	541	Bo03215s010.1	NBS	At_WRR12 ortholog	different	
901	Bju_WRR1 (CNL)	Brassica oleracea (v2.1)	Scaffold03384	1,212	283	4.00E-128	78%	412	Bo03384s010.1	CNL	Bju_WRR1 ortholog	same	
902	At_WRR12 (TNL)	Brassica oleracea (v2.1)	Scaffold03712	1,118	165	2.00E-82	82.40%	274	Bo03712s010.1	LRR-RLP	At_WRR12 ortholog	different	
903	Bju_WRR1 (CNL)	Brassica oleracea (v2.1)	Scaffold07027	557	159	5.00E-75	79.80%	256	Bo07027s010.1		Bju_WRR1 ortholog	lost	
904	At_RPP2b (TNL)	Brassica oleracea (v2.1)	Scaffold08138	621	206	3.00E-92	76.20%	300	Bo08138s010.1		At_RPP2b ortholog	lost	
905	At_RPP2b (TNL)	Brassica oleracea (v2.1)	Scaffold08794	6	206	2.00E-77	71.30%	259	Bo08794s010.1	NBS	At_RPP2b ortholog	different	
906	Bna_Rlm9/4/7 (Other-RLK)	Brassica rapa (v3.0)	A01	3,215,570	309	6.00E-114	70.80%	389	BraA01g006710.3C		Bna_Rlm9/4/7 ortholog	lost	
907	At_RPS2 (NL)	Brassica rapa (v3.0)	A01	8,942,719	930	0	83%	1470	BraA01g016810.3C	CNL	At_RPS2 ortholog	different	
908	At_NDR1 (TM)	Brassica rapa (v3.0)	A01	22,508,794	218	4.00E-101	75.20%	328	BraA01g032990.3C		At_NDR1 ortholog	lost	
909	Bna_MAPk (Other-RLK)	Brassica rapa (v3.0)	A01	25,223,445	181	3.00E-157	82.30%	301	BraA01g037110.3C		Bna_MAPk ortholog	lost	
910	At_RPM1 (NL)	Brassica rapa (v3.0)	A01	27,901,612	235	1.00E-168	71.90%	312	BraA01g041700.3C		At_RPM1 ortholog	lost	
911	At_PBS1 (STK)	Brassica rapa (v3.0)	A02	367,975	223	7.00E-131	73.90%	344	BraA02g000620.3C		At_PBS1 ortholog	lost	
912	At_BAK1 (LRR-RLK)	Brassica rapa (v3.0)	A02	1,673,101	247	1.00E-108	70.40%	367	BraA02g003410.3C	LRR-RLK	At_BAK1 ortholog	same	
913	At_PBS1 (STK)	Brassica rapa (v3.0)	A02	2,250,166	317	3.00E-169	83.90%	536	BraA02g004680.3C		At_PBS1 ortholog	lost	
914	At_FLS2 (LRR-RLK)	Brassica rapa (v3.0)	A02	22,615,974	509	0	75.20%	737	BraA02g032770.3C	LRR-RLK	At_FLS2 ortholog	same	
915	At_FLS2 (LRR-RLK)	Brassica rapa (v3.0)	A02	22,617,499	502	0	78.60%	689	BraA02g032770.3C	LRR-RLK	At_FLS2 ortholog	same	
916	At_NRG1a (RNL)	Brassica rapa (v3.0)	A02	28,419,031	242	4.00E-95	75.20%	334	BraA02g040960.3C	NL	At_NRG1a ortholog	different	
917	At_NRG1b (RNL)	Brassica rapa (v3.0)	A02	28,419,037	244	3.00E-98	73.70%	343	BraA02g040960.3C	NL	At_NRG1b ortholog	different	
918	At_PBS1 (STK)	Brassica rapa (v3.0)	A03	2,425,766	164	3.00E-67	74.30%	240	BraA03g005610.3C		At_PBS1 ortholog	lost	
919	At_PBS1 (STK)	Brassica rapa (v3.0)	A03	2,427,053	151	4.00E-133	79.40%	218	BraA03g005610.3C		At_PBS1 ortholog	lost	
920	At_SOBIR1 (LRR-RLK)	Brassica rapa (v3.0)	A03	7,877,080	647	0	80.60%	972	BraA03g016980.3C	LRR-RLK	At_SOBIR1 ortholog	same	
921	Bna_Rlm9/4/7 (Other-RLK)	Brassica rapa (v3.0)	A03	15,369,405	363	2.00E-141	73.80%	469	BraA03g030670.3C		Bna_Rlm9/4/7 ortholog	lost	
922	At_NDR1 (TM)	Brassica rapa (v3.0)	A03	19,530,877	220	3.00E-101	78.60%	329	BraA03g039450.3C		At_NDR1 ortholog	lost	
923	At_BAK1 (LRR-RLK)	Brassica rapa (v3.0)	A03	21,319,593	264	1.00E-106	70.40%	361	BraA03g042450.3C	LRR-RLK	At_BAK1 ortholog	same	
924	Bol_FocBo1 (TNL)	Brassica rapa (v3.0)	A03	23,874,310	360	0	71.60%	448	BraA03g047230.3C	TNL	Bol_FocBo1 ortholog	same	1
925	Bol_FocBo1 (TNL)	Brassica rapa (v3.0)	A03	23,874,872	154	0	79.20%	222	BraA03g047230.3C	TNL	Bol_FocBo1 ortholog	same	1
926	Bol_FocBo1 (TNL)	Brassica rapa (v3.0)	A03	23,879,360	647	0	73.40%	890	BraA03g047240.3C	TNL	Bol_FocBo1 ortholog	same	1
927	Bol_FocBo1 (TNL)	Brassica rapa (v3.0)	A03	23,880,778	311	0	80%	492	BraA03g047240.3C	TNL	Bol_FocBo1 ortholog	same	1
928	Bol_FocBo1 (TNL)	Brassica rapa (v3.0)	A03	23,881,953	361	0	95.20%	607	BraA03g047240.3C	TNL	Bol_FocBo1 ortholog	same	1
929	At_RPP5 (TNL)	Brassica rapa (v3.0)	A03	23,882,470	149	0	71.10%	224	BraA03g047240.3C	TNL	At_RPP5 ortholog	same	1
930	Bol_FocBo1 (TNL)	Brassica rapa (v3.0)	A03	23,882,473	149	0	89.20%	249	BraA03g047240.3C	TNL	Bol_FocBo1 ortholog	same	1

931	*Bra_cRa/cRb (TNL)*	Brassica rapa (v3.0)	A03	25,527,348	316	0	75.90%	484	BraA03g049830.3C	NL	Bra_cRa/cRb paralog (tandem)	different	2
932	*Bra_cRa/cRb (TNL)*	Brassica rapa (v3.0)	A03	25,527,348	316	0.00E+00	75.90%	484	BraA03g049830.3C	NL	Bra_cRa/cRb paralog (tandem)	different	2
933	*Bra_Crr1a (TNL)*	Brassica rapa (v3.0)	A03	25,545,134	149	1.00E-62	74.40%	239	BraA03g049840.3C	TX	Bra_Crr1a paralog (segmented)	different	2
934	*Bra_cRa/cRb (TNL)*	Brassica rapa (v3.0)	A03	25,545,344	219	6.00E-87	88.50%	318	BraA03g049840.3C	TX	Bra_cRa/cRb paralog (tandem)	different	2
935	*Bra_cRa/cRb (TNL)*	Brassica rapa (v3.0)	A03	25,550,991	368	0	77.10%	585	BraA03g049860.3C	TNL	Bra_cRa/cRb gene		2
936	*Bra_cRa/cRb (TNL)*	Brassica rapa (v3.0)	A03	25,550,991	368	0	77.10%	585	BraA03g049860.3C	TNL	Bra_cRa/cRb gene		2
937	*Bra_cRa/cRb (TNL)*	Brassica rapa (v3.0)	A03	25,553,090	149	3.00E-77	88.50%	286	BraA03g049860.3C	TNL	Bra_cRa/cRb gene		2
938	*Bra_Crr1a (TNL)*	Brassica rapa (v3.0)	A03	25,553,321	226	9.00E-76	71.60%	281	BraA03g049860.3C	TNL	Bra_Crr1a paralog (segmented)	same	2
939	*Bra_cRa/cRb (TNL)*	Brassica rapa (v3.0)	A03	25,560,459	209	3.00E-69	71.70%	253	BraA03g049870.3C	TX	Bra_cRa/cRb paralog (tandem)	different	2
940	*At_BAK1 (LRR-RLK)*	Brassica rapa (v3.0)	A03	30,465,652	405	0	83.90%	661	BraA03g057870.3C	LRR-RLK	At_BAK1 ortholog	same	
941	*At_SOBIR1 (LRR-RLK)*	Brassica rapa (v3.0)	A04	16,963,507	646	0	76%	880	BraA04g023000.3C	LRR-RLK	At_SOBIR1 ortholog	same	3
942	*At_SOBIR1 (LRR-RLK)*	Brassica rapa (v3.0)	A04	16,977,182	643	0	78.20%	923	BraA04g023010.3C	LRR-RLK	At_SOBIR1 ortholog	same	3
943	*Bna_MAPk (Other-RLK)*	Brassica rapa (v3.0)	A05	1,726,702	297	1.00E-166	72.70%	423	BraA05g003220.3C		Bna_MAPk ortholog	lost	
944	*Bju_WRR1 (CNL)*	Brassica rapa (v3.0)	A05	4,631,348	272	1.00E-135	88.60%	477	BraA05g008810.3C		Bju_WRR1 ortholog	lost	4
945	*Bju_WRR1 (CNL)*	Brassica rapa (v3.0)	A05	4,644,183	575	0	86%	951	BraA05g008840.3C	NL	Bju_WRR1 ortholog	different	4
946	*Bju_WRR1 (CNL)*	Brassica rapa (v3.0)	A05	4,655,623	370	0	88.60%	653	BraA05g008850.3C		Bju_WRR1 ortholog	lost	4
947	*Bju_WRR1 (CNL)*	Brassica rapa (v3.0)	A05	4,662,707	580	0	81.50%	882	BraA05g008860.3C	NL	Bju_WRR1 ortholog	different	4
948	*Bju_WRR1 (CNL)*	Brassica rapa (v3.0)	A05	4,666,017	159	0	74.80%	231	BraA05g008870.3C		Bju_WRR1 ortholog	lost	4
949	*Bju_WRR1 (CNL)*	Brassica rapa (v3.0)	A05	4,666,477	247	0	84.20%	399	BraA05g008870.3C		Bju_WRR1 ortholog	lost	4
950	*Bju_WRR1 (CNL)*	Brassica rapa (v3.0)	A05	4,674,900	301	0	87.70%	547	BraA05g008890.3C	NL	Bju_WRR1 ortholog	different	4
951	*Bju_WRR1 (CNL)*	Brassica rapa (v3.0)	A05	4,678,856	588	0	82.60%	920	BraA05g008890.3C	NL	Bju_WRR1 ortholog	different	4
952	*Bju_WRR1 (CNL)*	Brassica rapa (v3.0)	A05	4,686,427	310	9.00E-168	82.90%	520	BraA05g008900.3C		Bju_WRR1 ortholog	lost	4
953	*Bju_WRR1 (CNL)*	Brassica rapa (v3.0)	A05	4,694,289	570	0	85.90%	941	BraA05g008910.3C	NL	Bju_WRR1 ortholog	different	4
954	*Bju_WRR1 (CNL)*	Brassica rapa (v3.0)	A05	4,709,869	310	5.00E-177	86.40%	538	BraA05g008930.3C	NL	Bju_WRR1 ortholog	different	4
955	*Bju_WRR1 (CNL)*	Brassica rapa (v3.0)	A05	4,713,332	292	0	82.80%	471	BraA05g008930.3C	NL	Bju_WRR1 ortholog	different	4
956	*Bju_WRR1 (CNL)*	Brassica rapa (v3.0)	A05	4,714,270	208	0	78.30%	286	BraA05g008940.3C		Bju_WRR1 ortholog	lost	5
957	*Bju_WRR1 (CNL)*	Brassica rapa (v3.0)	A05	4,719,461	302	2.00E-164	92.30%	566	BraA05g008940.3C		Bju_WRR1 ortholog	lost	5
958	*Bju_WRR1 (CNL)*	Brassica rapa (v3.0)	A05	4,737,413	148	2.00E-54	72.90%	215	BraA05g008950.3C	NL	Bju_WRR1 ortholog	different	5
959	*Bju_WRR1 (CNL)*	Brassica rapa (v3.0)	A05	4,739,352	372	0	85.40%	625	BraA05g008950.3C	NL	Bju_WRR1 ortholog	different	5
960	*Bju_WRR1 (CNL)*	Brassica rapa (v3.0)	A05	4,740,458	183	0	76.50%	269	BraA05g008960.3C		Bju_WRR1 ortholog	lost	5
961	*Bju_WRR1 (CNL)*	Brassica rapa (v3.0)	A05	4,746,583	311	7.00E-174	85.50%	532	BraA05g008970.3C		Bju_WRR1 ortholog	lost	5
962	*Bju_WRR1 (CNL)*	Brassica rapa (v3.0)	A05	4,752,683	603	0	82%	934	BraA05g008980.3C	NL	Bju_WRR1 ortholog	different	5
963	*Bju_WRR1 (CNL)*	Brassica rapa (v3.0)	A05	4,760,122	382	0	85%	641	BraA05g008990.3C	CNL	Bju_WRR1 ortholog	same	5
964	*Bju_WRR1 (CNL)*	Brassica rapa (v3.0)	A05	4,761,693	585	0	78.80%	871	BraA05g008990.3C	CNL	Bju_WRR1 ortholog	same	5
965	*Bju_WRR1 (CNL)*	Brassica rapa (v3.0)	A05	4,772,648	299	2.00E-177	86.60%	526	BraA05g009010.3C	NL	Bju_WRR1 ortholog	different	5
966	*Bju_WRR1 (CNL)*	Brassica rapa (v3.0)	A05	4,777,402	593	0	81.20%	915	BraA05g009010.3C	NL	Bju_WRR1 ortholog	different	5
967	*Bju_WRR1 (CNL)*	Brassica rapa (v3.0)	A05	4,793,416	308	2.00E-166	81.10%	493	BraA05g009040.3C		Bju_WRR1 ortholog	lost	5
968	*Bju_WRR1 (CNL)*	Brassica rapa (v3.0)	A05	4,801,799	587	0	82.70%	918	BraA05g009050.3C	NL	Bju_WRR1 ortholog	different	5
969	*Bna_MAPk (Other-RLK)*	Brassica rapa (v3.0)	A05	10,133,718	222	2.00E-93	72.90%	322	BraA05g016950.3C		Bna_MAPk ortholog	lost	
970	*At_RFO3 (Other-RLK)*	Brassica rapa (v3.0)	A05	22,785,744	350	3.00E-172	71.40%	461	BraA05g031110.3C	Other-RLK	At_RFO3 ortholog	same	
971	*Bna_Rlm9/4/7 (Other-RLK)*	Brassica rapa (v3.0)	A06	7,992,030	332	2.00E-127	76.50%	429	BraA06g014990.3C	Other-RLK	Bna_Rlm9/4/7 ortholog	same	
972	*At_NRG1a (RNL)*	Brassica rapa (v3.0)	A06	22,660,222	286	2.00E-119	76.50%	405	BraA06g033140.3C	NL	At_NRG1a ortholog	different	
973	*At_NRG1b (RNL)*	Brassica rapa (v3.0)	A06	22,660,222	286	4.00E-118	74.40%	402	BraA06g033140.3C	NL	At_NRG1b ortholog	different	
974	*Bju_WRR1 (CNL)*	Brassica rapa (v3.0)	A06	23,792,495	299	3.00E-119	72.20%	425	BraA06g035130.3C	NL	Bju_WRR1 ortholog	different	6
975	*Bju_WRR1 (CNL)*	Brassica rapa (v3.0)	A06	23,829,329	314	3.00E-139	72.60%	444	BraA06g035180.3C	NL	Bju_WRR1 ortholog	different	6
976	*At_BAK1 (LRR-RLK)*	Brassica rapa (v3.0)	A07	5,133,133	157	1.00E-129	71.90%	232	BraA07g006070.3C	LRR-RLK	At_BAK1 ortholog	same	
977	*At_NRG1a (RNL)*	Brassica rapa (v3.0)	A07	14,812,850	283	6.00E-103	71%	357	BraA07g017010.3C	NL	At_NRG1a ortholog	different	7
978	*At_NRG1b (RNL)*	Brassica rapa (v3.0)	A07	14,812,886	271	2.00E-146	71.90%	367	BraA07g017010.3C	NL	At_NRG1b ortholog	different	7
979	*At_NRG1a (RNL)*	Brassica rapa (v3.0)	A07	14,823,110	286	6.00E-98	70.20%	342	BraA07g017020.3C	NL	At_NRG1a ortholog	different	7
980	*At_NRG1b (RNL)*	Brassica rapa (v3.0)	A07	14,823,128	279	5.00E-102	70.20%	354	BraA07g017020.3C	NL	At_NRG1b ortholog	different	7
981	*Bna_Rlm9/4/7 (Other-RLK)*	Brassica rapa (v3.0)	A07	19,978,761	343	0	91.20%	606	BraA07g026110.3C	Other-RLK	Bna_Rlm9/4/7 ortholog	same	
982	*Bna_Rlm9/4/7 (Other-RLK)*	Brassica rapa (v3.0)	A07	19,980,132	419	0	97.10%	724	BraA07g026110.3C	Other-RLK	Bna_Rlm9/4/7 ortholog	same	
983	*At_BAK1 (LRR-RLK)*	Brassica rapa (v3.0)	A07	21,715,000	247	2.00E-176	96.70%	506	BraA07g029410.3C	LRR-RLK	At_BAK1 ortholog	same	
984	*At_BAK1 (LRR-RLK)*	Brassica rapa (v3.0)	A07	25,435,198	271	4.00E-152	88.10%	493	BraA07g036270.3C	LRR-RLK	At_BAK1 ortholog	same	
985	*Bna_Rlm9/4/7 (Other-RLK)*	Brassica rapa (v3.0)	A07	28,459,325	424	0	80.40%	603	BraA07g042560.3C	Other-RLK	Bna_Rlm9/4/7 ortholog	same	
986	*Bra_Crr1a (TNL)*	Brassica rapa (v3.0)	A08	11,696,782	199	1.00E-80	78.80%	297	BraA08g013970.3C	TX	Bra_Crr1a paralog (tandem)	different	
987	*Bra_Crr1a (TNL)*	Brassica rapa (v3.0)	A08	12,275,017	375	0	95.70%	701	BraA08g014830.3C	TNL	Bra_Crr1a gene		
988	*Bra_cRa/cRb (TNL)*	Brassica rapa (v3.0)	A08	12,275,886	149	2.00E-62	75.10%	239	BraA08g014830.3C	TNL	Bra_cRa/cRb paralog (segmented	same	
989	*Bra_Crr1a (TNL)*	Brassica rapa (v3.0)	A08	12,276,051	204	3.00E-101	98%	362	BraA08g014830.3C	TNL	Bra_Crr1a gene		
990	*At_RFO2 (LRR-RLP)*	Brassica rapa (v3.0)	A08	20,242,694	734	0	70.80%	926	BraA08g029370.3C	LRR-RLP	At_RFO2 ortholog	same	
991	*At_RPP8 (CNL)*	Brassica rapa (v3.0)	A08	21,456,930	293	5.00E-103	73%	360	BraA08g032140.3C	NL	At_RPP8 ortholog	different	
992	*Bna_Rlm9/4/7 (Other-RLK)*	Brassica rapa (v3.0)	A08	22,418,732	399	2.00E-156	74.60%	513	BraA08g034600.3C		Bna_Rlm9/4/7 ortholog	lost	
993	*At_RPP13 (CNL)*	Brassica rapa (v3.0)	A09	5,222,211	455	0	74%	538	BraA09g009110.3C	NBS	At_RPP13 ortholog	different	
994	*At_NRG1a (RNL)*	Brassica rapa (v3.0)	A09	5,307,195	217	9.00E-76	74.60%	276	BraA09g009300.3C	CNL	At_NRG1a ortholog	different	
995	*At_NRG1a (RNL)*	Brassica rapa (v3.0)	A09	5,308,080	257	1.00E-145	85.20%	439	BraA09g009300.3C	CNL	At_NRG1a ortholog	different	
996	*At_NRG1a (RNL)*	Brassica rapa (v3.0)	A09	5,309,364	284	3.00E-129	83%	434	BraA09g009300.3C	CNL	At_NRG1a ortholog	different	
997	*At_NRG1b (RNL)*	Brassica rapa (v3.0)	A09	5,309,364	284	3.00E-131	81.60%	440	BraA09g009300.3C	CNL	At_NRG1b ortholog	different	
998	*At_RLM1b (TNL)*	Brassica rapa (v3.0)	A09	8,722,997	149	1.00E-65	77.10%	248	BraA09g014870.3C	TN	At_RLM1b ortholog	different	8
999	*At_RLM1b (TNL)*	Brassica rapa (v3.0)	A09	8,723,631	375	4.00E-155	70.40%	522	BraA09g014870.3C	TN	At_RLM1b ortholog	different	8
1000	*At_RLM1b (TNL)*	Brassica rapa (v3.0)	A09	8,725,844	149	6.00E-67	79.10%	252	BraA09g014880.3C	TNL	At_RLM1b ortholog	same	8
1001	*At_RLM1b (TNL)*	Brassica rapa (v3.0)	A09	8,752,770	149	3.00E-67	78.50%	253	BraA09g014900.3C	TNL	At_RLM1b ortholog	same	8
1002	*At_RLM1b (TNL)*	Brassica rapa (v3.0)	A09	8,863,567	150	2.00E-58	70.60%	225	BraA09g015080.3C	Other-NLR	At_RLM1b ortholog	different	8
1003	*At_RPS4 (TNL)*	Brassica rapa (v3.0)	A09	13,753,052	515	0	75.90%	762	BraA09g021150.3C	TNL	At_RPS4 ortholog	same	
1004	*At_FLS2 (LRR-RLK)*	Brassica rapa (v3.0)	A09	14,232,317	1029	0	80.80%	1539	BraA09g021780.3C	LRR-RLK	At_FLS2 ortholog	same	
1005	*Bna_MAPk (Other-RLK)*	Brassica rapa (v3.0)	A09	15,285,814	149	9.00E-56	77.80%	210	BraA09g023130.3C		Bna_MAPk ortholog	lost	
1006	*Bna_Rlm9/4/7 (Other-RLK)*	Brassica rapa (v3.0)	A09	40,507,682	351	4.00E-140	74.90%	466	BraA09g056650.3C		Bna_Rlm9/4/7 ortholog	lost	
1007	*At_WRR12 (TNL)*	Brassica rapa (v3.0)	A09	40,957,407	368	0	82.60%	550	BraA09g057600.3C	TNL	At_WRR12 ortholog	same	9
1008	*At_WRR12 (TNL)*	Brassica rapa (v3.0)	A09	40,959,002	504	0	80.10%	823	BraA09g057600.3C	TNL	At_WRR12 ortholog	same	9
1009	*At_WRR12 (TNL)*	Brassica rapa (v3.0)	A09	40,959,785	149	2.00E-68	85.20%	260	BraA09g057600.3C	TNL	At_WRR12 ortholog	same	9
1010	*At_WRR12 (TNL)*	Brassica rapa (v3.0)	A09	40,961,385	411	6.00E-145	74.90%	503	BraA09g057610.3C	Other-NLR	At_WRR12 ortholog	different	9
1011	*Bna_Rlm9/4/7 (Other-RLK)*	Brassica rapa (v3.0)	A09	41,410,932	340	7.00E-132	72%	442	BraA09g058390.3C		Bna_Rlm9/4/7 ortholog	lost	
1012	*At_PBS1 (STK)*	Brassica rapa (v3.0)	A09	43,634,283	218	7.00E-99	71.10%	327	BraA09g063070.3C		At_PBS1 ortholog	lost	
1013	*At_RLP1 (LRR-RLP)*	Brassica rapa (v3.0)	A09	43,768,475	733	0	80.90%	1099	BraA09g063380.3C		At_RLP1 ortholog	lost	
1014	*At_RLP1 (LRR-RLP)*	Brassica rapa (v3.0)	A09	43,770,658	146	5.00E-56	74.60%	217	BraA09g063380.3C		At_RLP1 ortholog	lost	
1015	*Bna_Rlm9/4/7 (Other-RLK)*	Brassica rapa (v3.0)	A10	1,787,230	423	8.00E-164	73.70%	535	BraA10g003440.3C	Other-RLK	Bna_Rlm9/4/7 ortholog	same	
1016	*Bna_MAPk (Other-RLK)*	Brassica rapa (v3.0)	A10	15,219,939	255	5.00E-167	70.90%	336	BraA10g021230.3C		Bna_MAPk ortholog	lost	
1017	*At_PBS1 (STK)*	Brassica rapa (v3.0)	A10	17,211,860	306	6.00E-175	90.10%	552	BraA10g025590.3C		At_PBS1 ortholog	lost	
1018	*Bna_LepR3/Rlm2 (LRR-RLP)*	Brassica rapa (v3.0)	A10	17,540,233	739	0	84.90%	1034	BraA10g026360.3C		Bna_LepR3/Rlm2 ortholog	lost	

Note:

*Arrangement based on the genes position in its respective genome.

Red fonts means the cloned gene was represented by the gene in a database based on the similarity analysis.

Yellow box means the gene exist as homolog to at least two cloned genes.

Reference:

Li P, Quan X, Jia G, Xiao J, Cloutier S, You FM, 2016. RGAugury: a pipeline for genome-wide prediction of resistance gene analogs (RGAs) in plants. BMC Genomics **17**, 852.

Lyons E, Pedersen B, Kane J, et al., 2008. Finding and Comparing Syntenic Regions among Arabidopsis and the Outgroups Papaya, Poplar, and Grape: CoGe with Rosids. Plant physiology **148**, 1772.

Tirnaz S, Bayer P, Inturrisi F, et al., 2020. Resistance gene analogs in the Brassicaceae: Identification, characterization, distribution and evolution. Plant physiology **184**, 909–22.
