# Supplementary material for: Mining of Cloned Disease Resistance Gene Homologs (CDRHs) in Brassica Species and Arabidopsis thaliana
Source: Biology (Basel). 2022 May 26;11(6):821. doi: 10.3390/biology11060821 (PMC9220128; doi:10.3390/biology11060821)
Supplement: Supplementary file 1 [file biology-11-00821-s001.zip › Table S2.pdf]

Table S2. List of resistance gene analogs (RGAs) in *Brassica carinata* zd-1 v1.0 identified by RGAugury pipeline.

**Number**	**Gene**	**RGA type**
1	BcaC04g22760	CN
2	BcaC07g38502	CN
3	BcaB02g09974	CN
4	BcaNung05332	CN
5	BcaC08g43505	CN
6	BcaB07g31340	CN
7	BcaC03g16764	CN
8	BcaC04g19598	CN
9	BcaC07g38510	CN
10	BcaB07g31351	CN
11	BcaC09g51441	CN
12	BcaC03g16769	CN
13	BcaC01g01216	CNL
14	BcaC03g17971	CNL
15	BcaB06g25378	CNL
16	BcaB08g34626	CNL
17	BcaC07g42571	CNL
18	BcaB07g33473	CNL
19	BcaB08g36407	CNL
20	BcaB07g30851	CNL
21	BcaC06g33794	CNL
22	BcaB02g09973	CNL
23	BcaC04g22424	CNL
24	BcaC04g23168	CNL
25	BcaC09g49372	CNL
26	BcaB08g34460	CNL
27	BcaB03g13168	CNL
28	BcaC08g43336	CNL
29	BcaC06g31794	CNL
30	BcaC07g41717	CNL
31	BcaB01g00645	CNL
32	BcaB02g07270	CNL
33	BcaC06g30745	CNL
34	BcaB07g31336	CNL
35	BcaC04g19685	CNL
36	BcaC07g39476	CNL
37	BcaC01g03216	CNL
38	BcaC08g43237	CNL
39	BcaC06g30724	CNL
40	BcaC06g34474	CNL
41	BcaC05g25204	CNL
42	BcaC02g08928	CNL
43	BcaB02g09946	CNL
44	BcaB03g15642	CNL
45	BcaB07g31264	CNL
46	BcaC02g11752	CNL
47	BcaC09g49497	CNL
48	BcaNung05799	CNL
49	BcaC07g42561	CNL
50	BcaB03g16340	CNL
51	BcaB04g20089	CNL
52	BcaB04g20286	CNL
53	BcaC06g34429	CNL
54	BcaC01g04663	CNL
55	BcaC01g01510	CNL
56	BcaB07g31338	CNL
57	BcaC04g21725	CNL
58	BcaB01g00640	CNL
59	BcaC06g30971	CNL
60	BcaC09g50671	CNL

61	BcaNung04015	CNL
62	BcaC09g50597	CNL
63	BcaB03g13138	CNL
64	BcaC09g49457	CNL
65	BcaB04g20195	CNL
66	BcaB04g20092	CNL
67	BcaB07g33472	CNL
68	BcaB03g15688	CNL
69	BcaB07g31810	CNL
70	BcaB01g00559	CNL
71	BcaB03g13413	CNL
72	BcaC06g31548	CNL
73	BcaC04g22316	CNL
74	BcaB03g13197	CNL
75	BcaC02g11243	CNL
76	BcaC07g41711	CNL
77	BcaNung04016	CNL
78	BcaC05g25496	CNL
79	BcaB08g34547	CNL
80	BcaC06g34763	CNL
81	BcaB03g13136	CNL
82	BcaC05g29069	CNL
83	BcaC07g39565	CNL
84	BcaC07g41710	CNL
85	BcaC07g38504	CNL
86	BcaC08g42981	CNL
87	BcaC08g43199	CNL
88	BcaB04g20245	CNL
89	BcaB07g30879	NBS
90	BcaC04g18749	NBS
91	BcaC04g22423	NBS
92	BcaC05g25208	NBS
93	BcaC07g41368	NBS
94	BcaC04g23200	NBS
95	BcaC08g43442	NBS
96	BcaC07g39468	NBS
97	BcaC06g32254	NBS
98	BcaC08g45686	NBS
99	BcaB03g15687	NBS
100	BcaC09g49766	NBS
101	BcaC03g16734	NBS
102	BcaB06g27432	NBS
103	BcaB05g23829	NBS
104	BcaNung04012	NBS
105	BcaC03g15429	NBS
106	BcaB04g20325	NBS
107	BcaB01g00987	NBS
108	BcaC06g34725	NBS
109	BcaB06g27430	NBS
110	BcaC03g17975	NBS
111	BcaC05g26816	NBS
112	BcaB07g31341	NBS
113	BcaC07g42563	NBS
114	BcaC09g48552	NBS
115	BcaC03g16850	NBS
116	BcaB07g30442	NBS
117	BcaB05g23536	NBS
118	BcaC07g42564	NBS
119	BcaC03g13560	NBS
120	BcaC06g34466	NBS
121	BcaC02g10435	NBS
122	BcaC03g15431	NBS

123	BcaB04g20688	NBS
124	BcaC05g25205	NBS
125	BcaC07g41721	NBS
126	BcaB01g02604	NBS
127	BcaC03g13559	NBS
128	BcaC08g46726	NBS
129	BcaB02g10007	NBS
130	BcaC04g22191	NBS
131	BcaB03g15686	NBS
132	BcaC06g34459	NBS
133	BcaB05g24788	NBS
134	BcaB03g15685	NBS
135	BcaB03g13199	NBS
136	BcaC07g38497	NL
137	BcaC04g22178	NL
138	BcaC04g21215	NL
139	BcaB07g29885	NL
140	BcaC04g18747	NL
141	BcaB05g23777	NL
142	BcaB07g31172	NL
143	BcaC09g52139	NL
144	BcaB07g31050	NL
145	BcaB06g27426	NL
146	BcaC01g00616	NL
147	BcaB06g29169	NL
148	BcaB01g01704	NL
149	BcaC05g25207	NL
150	BcaC06g32706	NL
151	BcaC07g42585	NL
152	BcaC04g23834	NL
153	BcaC07g41707	NL
154	BcaC05g25198	NL
155	BcaB03g15473	NL
156	BcaB07g31186	NL
157	BcaB07g30034	NL
158	BcaC08g44809	NL
159	BcaB07g31235	NL
160	BcaC09g51572	NL
161	BcaC04g22151	NL
162	BcaC06g32707	NL
163	BcaC05g24943	NL
164	BcaB07g31051	NL
165	BcaB03g12806	NL
166	BcaB08g36730	NL
167	BcaC04g22277	NL
168	BcaC07g38695	NL
169	BcaB04g20232	NL
170	BcaC02g10433	NL
171	BcaB01g05424	NL
172	BcaC03g16735	NL
173	BcaC01g00997	NL
174	BcaC08g45691	NL
175	BcaB07g30780	NL
176	BcaC09g52148	NL
177	BcaC04g21188	NL
178	BcaNung03053	NL
179	BcaNung05226	NL
180	BcaC07g38501	NL
181	BcaNung04011	NL
182	BcaC08g47517	NL
183	BcaC06g34481	NL
184	BcaC08g43333	NL

185	BcaB01g00505	NL
186	BcaC04g22762	NL
187	BcaB01g06296	NL
188	BcaB05g23289	NL
189	BcaC04g23170	NL
190	BcaB07g29985	NL
191	BcaB01g01707	NL
192	BcaC06g32180	NL
193	BcaB01g06293	NL
194	BcaB07g30782	NL
195	BcaC04g21790	NL
196	BcaB03g13297	NL
197	BcaB06g25379	NL
198	BcaB03g15021	NL
199	BcaC07g42581	NL
200	BcaB01g06294	NL
201	BcaB03g13202	NL
202	BcaB07g29906	NL
203	BcaB07g31169	NL
204	BcaC03g17981	NL
205	BcaC03g13557	NL
206	BcaC05g29959	NL
207	BcaC07g38509	NL
208	BcaC08g44107	NL
209	BcaC02g07603	NL
210	BcaB03g13293	NL
211	BcaC07g41708	NL
212	BcaC08g44110	NL
213	BcaB07g29907	NL
214	BcaC09g47934	NL
215	BcaB05g21751	NL
216	BcaC09g51442	NL
217	BcaC06g34375	NL
218	BcaB06g27383	NL
219	BcaNung05233	NL
220	BcaC07g42587	NL
221	BcaC03g17818	NL
222	BcaB05g21752	NL
223	BcaB05g23375	NL
224	BcaB06g27435	NL
225	BcaC01g04610	NL
226	BcaC01g03111	NL
227	BcaC03g17979	NL
228	BcaB04g20091	NL
229	BcaC05g24942	NL
230	BcaC05g29070	NL
231	BcaC01g01891	NL
232	BcaC03g17207	OTHER
233	BcaB05g22688	OTHER
234	BcaC06g31568	OTHER
235	BcaB02g07886	OTHER
236	BcaB05g24905	OTHER
237	BcaB03g15684	OTHER
238	BcaNung02558	OTHER
239	BcaB03g12388	OTHER
240	BcaC06g34483	OTHER
241	BcaC08g46737	OTHER
242	BcaB01g02716	OTHER
243	BcaB03g15024	OTHER
244	BcaB05g23293	OTHER
245	BcaC06g31471	OTHER
246	BcaC03g13589	OTHER

247	BcaC01g02958	OTHER
248	BcaB03g15694	OTHER
249	BcaB01g04906	OTHER
250	BcaC08g45689	OTHER
251	BcaB08g34692	OTHER
252	BcaB03g12384	OTHER
253	BcaB02g06599	OTHER
254	BcaC02g07540	OTHER
255	BcaC01g04645	OTHER
256	BcaB03g15690	OTHER
257	BcaB08g35742	OTHER
258	BcaNung00823	OTHER
259	BcaC03g16731	OTHER
260	BcaC07g41370	OTHER
261	BcaB01g00438	OTHER
262	BcaB03g11621	OTHER
263	BcaB01g00504	OTHER
264	BcaC01g00474	OTHER
265	BcaB01g06317	OTHER
266	BcaC08g46731	TN
267	BcaC08g45683	TN
268	BcaB03g15692	TN
269	BcaC08g45692	TN
270	BcaNung01773	TN
271	BcaC07g41373	TN
272	BcaC04g18706	TN
273	BcaB08g34591	TN
274	BcaC04g21789	TN
275	BcaB08g36723	TN
276	BcaB03g15027	TN
277	BcaB07g30897	TN
278	BcaB05g24785	TN
279	BcaC05g30211	TN
280	BcaB07g30470	TN
281	BcaC03g13591	TN
282	BcaC05g25682	TN
283	BcaB06g27427	TN
284	BcaB06g27434	TN
285	BcaB07g30170	TN
286	BcaC03g17453	TN
287	BcaB03g11626	TN
288	BcaB03g15693	TN
289	BcaC08g46730	TN
290	BcaC08g46728	TN
291	BcaB05g23295	TN
292	BcaC07g38148	TN
293	BcaB03g15022	TN
294	BcaB07g32560	TN
295	BcaB01g04882	TN
296	BcaC08g45684	TN
297	BcaC02g08956	TN
298	BcaC03g15433	TN
299	BcaC08g46733	TN
300	BcaNung01440	TN
301	BcaC02g11275	TNL
302	BcaB02g10408	TNL
303	BcaC03g13671	TNL
304	BcaNung02559	TNL
305	BcaC06g31268	TNL
306	BcaB07g30900	TNL
307	BcaC03g16733	TNL
308	BcaNung01775	TNL

309	BcaC04g22342	TNL
310	BcaB07g30904	TNL
311	BcaC07g40130	TNL
312	BcaC05g26337	TNL
313	BcaB07g30465	TNL
314	BcaC06g34869	TNL
315	BcaB03g11625	TNL
316	BcaB05g23830	TNL
317	BcaB05g23924	TNL
318	BcaC06g32926	TNL
319	BcaC09g48129	TNL
320	BcaB03g12360	TNL
321	BcaC04g21186	TNL
322	BcaNung02555	TNL
323	BcaC08g45693	TNL
324	BcaC01g00425	TNL
325	BcaC03g16858	TNL
326	BcaC07g38142	TNL
327	BcaC04g18695	TNL
328	BcaB01g04619	TNL
329	BcaB01g05422	TNL
330	BcaB07g30834	TNL
331	BcaB03g11975	TNL
332	BcaB07g33583	TNL
333	BcaB04g19249	TNL
334	BcaC03g13401	TNL
335	BcaB07g30466	TNL
336	BcaC09g51403	TNL
337	BcaC06g34374	TNL
338	BcaC07g41367	TNL
339	BcaC08g46725	TNL
340	BcaB07g30903	TNL
341	BcaC03g13587	TNL
342	BcaB03g12358	TNL
343	BcaB03g12991	TNL
344	BcaC02g09158	TNL
345	BcaB07g30835	TNL
346	BcaC04g18769	TNL
347	BcaB01g06322	TNL
348	BcaC03g16852	TNL
349	BcaC01g00430	TNL
350	BcaC05g29961	TNL
351	BcaB08g34693	TNL
352	BcaC01g00473	TNL
353	BcaC06g31566	TNL
354	BcaB08g36686	TNL
355	BcaC06g34479	TNL
356	BcaC05g29367	TNL
357	BcaC09g51400	TNL
358	BcaC06g32294	TNL
359	BcaB07g31538	TNL
360	BcaC08g44537	TNL
361	BcaC07g39355	TNL
362	BcaC04g22513	TNL
363	BcaNung02557	TNL
364	BcaC06g34465	TNL
365	BcaB02g07888	TNL
366	BcaB08g37355	TNL
367	BcaB05g22226	TNL
368	BcaC07g39246	TNL
369	BcaB07g31409	TNL
370	BcaC06g31567	TNL

371	BcaC07g38873	TNL
372	BcaB03g15226	TNL
373	BcaC04g22685	TNL
374	BcaC08g47492	TNL
375	BcaNung06446	TNL
376	BcaC06g33518	TNL
377	BcaC01g00754	TNL
378	BcaB07g30896	TNL
379	BcaB01g00439	TNL
380	BcaB07g30995	TNL
381	BcaC04g22512	TNL
382	BcaB07g31537	TNL
383	BcaB08g36752	TNL
384	BcaC05g29962	TNL
385	BcaB03g11720	TNL
386	BcaB08g36685	TNL
387	BcaB07g30028	TNL
388	BcaC09g51401	TNL
389	BcaNung06797	TNL
390	BcaC08g46727	TNL
391	BcaC09g47772	TNL
392	BcaC07g38028	TNL
393	BcaC08g47516	TNL
394	BcaC03g16854	TNL
395	BcaC01g00216	TNL
396	BcaC04g21896	TNL
397	BcaB07g30905	TNL
398	BcaC04g21783	TNL
399	BcaB05g24569	TNL
400	BcaC01g05800	TNL
401	BcaC04g22671	TNL
402	BcaB07g30471	TNL
403	BcaB01g04035	TNL
404	BcaC02g10369	TNL
405	BcaB01g05419	TNL
406	BcaNung07093	TNL
407	BcaC04g19247	TNL
408	BcaC03g17662	TNL
409	BcaB01g05161	TNL
410	BcaC04g22511	TNL
411	BcaB06g26204	TNL
412	BcaB07g31195	TNL
413	BcaB03g12262	TNL
414	BcaB01g00784	TNL
415	BcaB07g31554	TNL
416	BcaB07g30901	TNL
417	BcaC04g21784	TNL
418	BcaB03g12426	TNL
419	BcaB01g04898	TNL
420	BcaNung01716	TNL
421	BcaNung04529	TNL
422	BcaB01g06320	TNL
423	BcaB07g31365	TNL
424	BcaB08g36683	TNL
425	BcaC01g00618	TNL
426	BcaC05g29960	TNL
427	BcaC07g38875	TNL
428	BcaC03g13623	TNL
429	BcaC07g39242	TNL
430	BcaB06g27659	TNL
431	BcaB02g07887	TNL
432	BcaC07g40943	TNL

433	BcaB05g23288	TNL
434	BcaB07g31419	TNL
435	BcaB03g11622	TNL
436	BcaB07g32393	TNL
437	BcaC01g00424	TNL
438	BcaB07g32394	TNL
439	BcaC03g16895	TNL
440	BcaC07g41371	TNL
441	BcaC09g49199	TNL
442	BcaB07g30836	TNL
443	BcaB05g23320	TNL
444	BcaB06g27658	TNL
445	BcaC08g46126	TNL
446	BcaB07g30472	TNL
447	BcaB08g36734	TNL
448	BcaNung02640	TNL
449	BcaB07g30906	TNL
450	BcaC01g02955	TNL
451	BcaB06g27980	TNL
452	BcaB07g32547	TNL
453	BcaNung04533	TNL
454	BcaC02g07963	TNL
455	BcaB01g00515	TNL
456	BcaC09g50219	TNL
457	BcaB04g19347	TNL
458	BcaC04g22502	TNL
459	BcaB03g12355	TNL
460	BcaB03g11973	TNL
461	BcaC06g34470	TNL
462	BcaC03g16730	TNL
463	BcaB07g31552	TNL
464	BcaB01g01706	TNL
465	BcaC04g22207	TNL
466	BcaC01g04314	TNL
467	BcaC01g00315	TNL
468	BcaC06g34478	TNL
469	BcaB04g19246	TX
470	BcaC04g20121	TX
471	BcaB07g33376	TX
472	BcaB07g31234	TX
473	BcaC04g22111	TX
474	BcaB02g10970	TX
475	BcaC06g34492	TX
476	BcaC06g34467	TX
477	BcaB05g24786	TX
478	BcaNung04486	TX
479	BcaC08g46734	TX
480	BcaB07g31325	TX
481	BcaNung01059	TX
482	BcaC08g45690	TX
483	BcaC03g16732	TX
484	BcaB01g05192	TX
485	BcaB07g31561	TX
486	BcaB03g15025	TX
487	BcaB07g30919	TX
488	BcaC07g41807	TX
489	BcaC03g17361	TX
490	BcaB07g30907	TX
491	BcaC06g34005	TX
492	BcaC03g16714	TX
493	BcaC03g13648	TX
494	BcaC06g34482	TX

495	BcaB03g15476	TX
496	BcaB02g10236	TX
497	BcaB07g30914	TX
498	BcaC01g00642	TX
499	BcaB07g30915	TX
500	BcaB04g20175	TX
501	BcaB03g15689	TX
502	BcaC06g31181	TX
503	BcaNung01727	TX
504	BcaC02g07539	TX
505	BcaB03g15030	TX
506	BcaC06g31470	TX
507	BcaC09g51001	TX
508	BcaC03g15427	TX
509	BcaC04g22397	TX
510	BcaC03g16729	TX
511	BcaC04g21801	TX
512	BcaC06g34460	TX
513	BcaNung06112	TX
514	BcaC07g42026	TX
515	BcaC01g05728	TX
516	BcaC04g18699	TX
517	BcaNung00822	TX
518	BcaC09g49334	TX
519	BcaC03g13590	TX
520	BcaC06g31517	TX
521	BcaC09g49526	TX
522	BcaC08g45888	TX
523	BcaC04g22696	TX
524	BcaC06g31576	TX
525	BcaC06g34402	TX
526	BcaNung01060	TX
527	BcaC01g01343	TX
528	BcaC08g43474	TX
529	BcaC05g29958	TX
530	BcaC01g00422	TX
531	BcaC01g03122	TX
532	BcaB07g30469	TX
533	BcaC04g21800	TX
534	BcaB07g31265	TX
535	BcaC03g15425	TX
536	BcaC04g21809	TX
537	BcaC06g34376	TX
538	BcaNung00971	TX
539	BcaB01g05284	TX
540	BcaB03g12385	TX
541	BcaB04g17135	TX
542	BcaC06g34493	TX
543	BcaC08g44018	TX
544	BcaNung04488	TX
545	BcaB07g31259	TX
546	BcaC04g22276	TX
547	BcaB08g34534	TX
548	BcaC09g50983	TX
549	BcaB07g30468	TX
550	BcaB01g06297	TX
551	BcaB01g00027	RLK
552	BcaB01g00039	RLK
553	BcaB01g00048	RLK
554	BcaB01g00233	RLK
555	BcaB01g00235	RLK
556	BcaB01g00259	RLK

557	BcaB01g00425	RLK
558	BcaB01g00482	RLK
559	BcaB01g00489	RLK
560	BcaB01g00506	RLK
561	BcaB01g00903	RLK
562	BcaB01g00946	RLK
563	BcaB01g00990	RLK
564	BcaB01g01013	RLK
565	BcaB01g01016	RLK
566	BcaB01g01085	RLK
567	BcaB01g01086	RLK
568	BcaB01g01087	RLK
569	BcaB01g01090	RLK
570	BcaB01g01231	RLK
571	BcaB01g01604	RLK
572	BcaB01g01648	RLK
573	BcaB01g01653	RLK
574	BcaB01g01654	RLK
575	BcaB01g01766	RLK
576	BcaB01g01809	RLK
577	BcaB01g01810	RLK
578	BcaB01g01811	RLK
579	BcaB01g01814	RLK
580	BcaB01g01854	RLK
581	BcaB01g01858	RLK
582	BcaB01g01892	RLK
583	BcaB01g01897	RLK
584	BcaB01g01951	RLK
585	BcaB01g02025	RLK
586	BcaB01g02047	RLK
587	BcaB01g02242	RLK
588	BcaB01g02279	RLK
589	BcaB01g02405	RLK
590	BcaB01g02436	RLK
591	BcaB01g02449	RLK
592	BcaB01g02450	RLK
593	BcaB01g02458	RLK
594	BcaB01g02530	RLK
595	BcaB01g02586	RLK
596	BcaB01g02589	RLK
597	BcaB01g02590	RLK
598	BcaB01g02591	RLK
599	BcaB01g02592	RLK
600	BcaB01g02693	RLK
601	BcaB01g02694	RLK
602	BcaB01g02782	RLK
603	BcaB01g02820	RLK
604	BcaB01g02840	RLK
605	BcaB01g02860	RLK
606	BcaB01g02949	RLK
607	BcaB01g02950	RLK
608	BcaB01g02979	RLK
609	BcaB01g03012	RLK
610	BcaB01g03111	RLK
611	BcaB01g03198	RLK
612	BcaB01g03367	RLK
613	BcaB01g03504	RLK
614	BcaB01g03599	RLK
615	BcaB01g03650	RLK
616	BcaB01g03664	RLK
617	BcaB01g03717	RLK
618	BcaB01g03761	RLK

619	BcaB01g03793	RLK
620	BcaB01g03826	RLK
621	BcaB01g03845	RLK
622	BcaB01g03877	RLK
623	BcaB01g03993	RLK
624	BcaB01g04010	RLK
625	BcaB01g04067	RLK
626	BcaB01g04111	RLK
627	BcaB01g04135	RLK
628	BcaB01g04156	RLK
629	BcaB01g04242	RLK
630	BcaB01g04295	RLK
631	BcaB01g04302	RLK
632	BcaB01g04414	RLK
633	BcaB01g04478	RLK
634	BcaB01g04533	RLK
635	BcaB01g04534	RLK
636	BcaB01g04551	RLK
637	BcaB01g04559	RLK
638	BcaB01g04561	RLK
639	BcaB01g04575	RLK
640	BcaB01g04576	RLK
641	BcaB01g04578	RLK
642	BcaB01g04587	RLK
643	BcaB01g04649	RLK
644	BcaB01g04650	RLK
645	BcaB01g04651	RLK
646	BcaB01g04787	RLK
647	BcaB01g04813	RLK
648	BcaB01g04901	RLK
649	BcaB01g05010	RLK
650	BcaB01g05301	RLK
651	BcaB01g05303	RLK
652	BcaB01g05365	RLK
653	BcaB01g05428	RLK
654	BcaB01g05450	RLK
655	BcaB01g05551	RLK
656	BcaB01g06020	RLK
657	BcaB01g06102	RLK
658	BcaB01g06103	RLK
659	BcaB01g06126	RLK
660	BcaB01g06127	RLK
661	BcaB01g06235	RLK
662	BcaB01g06258	RLK
663	BcaB01g06259	RLK
664	BcaB01g06305	RLK
665	BcaB01g06339	RLK
666	BcaB02g06491	RLK
667	BcaB02g06592	RLK
668	BcaB02g06593	RLK
669	BcaB02g06594	RLK
670	BcaB02g06597	RLK
671	BcaB02g06728	RLK
672	BcaB02g06762	RLK
673	BcaB02g06829	RLK
674	BcaB02g06907	RLK
675	BcaB02g07231	RLK
676	BcaB02g07233	RLK
677	BcaB02g07483	RLK
678	BcaB02g07510	RLK
679	BcaB02g07681	RLK
680	BcaB02g07905	RLK

681	BcaB02g08003	RLK
682	BcaB02g08014	RLK
683	BcaB02g08015	RLK
684	BcaB02g08146	RLK
685	BcaB02g08213	RLK
686	BcaB02g08214	RLK
687	BcaB02g08384	RLK
688	BcaB02g08387	RLK
689	BcaB02g08410	RLK
690	BcaB02g08529	RLK
691	BcaB02g08909	RLK
692	BcaB02g08971	RLK
693	BcaB02g09257	RLK
694	BcaB02g09306	RLK
695	BcaB02g09407	RLK
696	BcaB02g09675	RLK
697	BcaB02g09736	RLK
698	BcaB02g09888	RLK
699	BcaB02g09939	RLK
700	BcaB02g09951	RLK
701	BcaB02g09990	RLK
702	BcaB02g10066	RLK
703	BcaB02g10159	RLK
704	BcaB02g10160	RLK
705	BcaB02g10220	RLK
706	BcaB02g10222	RLK
707	BcaB02g10234	RLK
708	BcaB02g10289	RLK
709	BcaB02g10319	RLK
710	BcaB02g10352	RLK
711	BcaB02g10354	RLK
712	BcaB02g10463	RLK
713	BcaB02g10740	RLK
714	BcaB02g10741	RLK
715	BcaB02g10784	RLK
716	BcaB02g10919	RLK
717	BcaB02g11039	RLK
718	BcaB02g11040	RLK
719	BcaB02g11041	RLK
720	BcaB02g11042	RLK
721	BcaB02g11043	RLK
722	BcaB02g11045	RLK
723	BcaB02g11164	RLK
724	BcaB02g11174	RLK
725	BcaB03g11663	RLK
726	BcaB03g11694	RLK
727	BcaB03g11748	RLK
728	BcaB03g11750	RLK
729	BcaB03g11758	RLK
730	BcaB03g11759	RLK
731	BcaB03g11903	RLK
732	BcaB03g11946	RLK
733	BcaB03g11974	RLK
734	BcaB03g11994	RLK
735	BcaB03g12102	RLK
736	BcaB03g12148	RLK
737	BcaB03g12172	RLK
738	BcaB03g12173	RLK
739	BcaB03g12174	RLK
740	BcaB03g12175	RLK
741	BcaB03g12206	RLK
742	BcaB03g12243	RLK

743	BcaB03g12286	RLK
744	BcaB03g12568	RLK
745	BcaB03g12670	RLK
746	BcaB03g12735	RLK
747	BcaB03g12741	RLK
748	BcaB03g12797	RLK
749	BcaB03g12823	RLK
750	BcaB03g12824	RLK
751	BcaB03g12825	RLK
752	BcaB03g12826	RLK
753	BcaB03g12827	RLK
754	BcaB03g12913	RLK
755	BcaB03g12914	RLK
756	BcaB03g13173	RLK
757	BcaB03g13257	RLK
758	BcaB03g13261	RLK
759	BcaB03g13265	RLK
760	BcaB03g13324	RLK
761	BcaB03g13465	RLK
762	BcaB03g13591	RLK
763	BcaB03g13610	RLK
764	BcaB03g13682	RLK
765	BcaB03g13838	RLK
766	BcaB03g13857	RLK
767	BcaB03g13860	RLK
768	BcaB03g13862	RLK
769	BcaB03g13863	RLK
770	BcaB03g13864	RLK
771	BcaB03g13927	RLK
772	BcaB03g14017	RLK
773	BcaB03g14039	RLK
774	BcaB03g14276	RLK
775	BcaB03g14397	RLK
776	BcaB03g14399	RLK
777	BcaB03g14504	RLK
778	BcaB03g14505	RLK
779	BcaB03g14506	RLK
780	BcaB03g14536	RLK
781	BcaB03g14570	RLK
782	BcaB03g14576	RLK
783	BcaB03g14577	RLK
784	BcaB03g14620	RLK
785	BcaB03g14632	RLK
786	BcaB03g14780	RLK
787	BcaB03g14804	RLK
788	BcaB03g14818	RLK
789	BcaB03g14819	RLK
790	BcaB03g14820	RLK
791	BcaB03g14962	RLK
792	BcaB03g14977	RLK
793	BcaB03g15071	RLK
794	BcaB03g15170	RLK
795	BcaB03g15192	RLK
796	BcaB03g15219	RLK
797	BcaB03g15221	RLK
798	BcaB03g15225	RLK
799	BcaB03g15229	RLK
800	BcaB03g15245	RLK
801	BcaB03g15289	RLK
802	BcaB03g15311	RLK
803	BcaB03g15314	RLK
804	BcaB03g15445	RLK

805	BcaB03g15515	RLK
806	BcaB03g15620	RLK
807	BcaB03g15668	RLK
808	BcaB03g15829	RLK
809	BcaB03g15886	RLK
810	BcaB03g16087	RLK
811	BcaB03g16216	RLK
812	BcaB03g16479	RLK
813	BcaB03g16637	RLK
814	BcaB03g16759	RLK
815	BcaB03g16760	RLK
816	BcaB03g16818	RLK
817	BcaB03g16881	RLK
818	BcaB04g16915	RLK
819	BcaB04g16917	RLK
820	BcaB04g16918	RLK
821	BcaB04g16940	RLK
822	BcaB04g17062	RLK
823	BcaB04g17158	RLK
824	BcaB04g17160	RLK
825	BcaB04g17168	RLK
826	BcaB04g17243	RLK
827	BcaB04g17419	RLK
828	BcaB04g17450	RLK
829	BcaB04g17876	RLK
830	BcaB04g17887	RLK
831	BcaB04g17949	RLK
832	BcaB04g18046	RLK
833	BcaB04g18468	RLK
834	BcaB04g18492	RLK
835	BcaB04g18860	RLK
836	BcaB04g18862	RLK
837	BcaB04g19006	RLK
838	BcaB04g19095	RLK
839	BcaB04g19110	RLK
840	BcaB04g19164	RLK
841	BcaB04g19356	RLK
842	BcaB04g19453	RLK
843	BcaB04g19537	RLK
844	BcaB04g19593	RLK
845	BcaB04g19846	RLK
846	BcaB04g19857	RLK
847	BcaB04g19897	RLK
848	BcaB04g19908	RLK
849	BcaB04g19915	RLK
850	BcaB04g20025	RLK
851	BcaB04g20108	RLK
852	BcaB04g20290	RLK
853	BcaB04g20334	RLK
854	BcaB04g20346	RLK
855	BcaB04g20389	RLK
856	BcaB04g20423	RLK
857	BcaB04g20536	RLK
858	BcaB04g20594	RLK
859	BcaB04g20654	RLK
860	BcaB04g20664	RLK
861	BcaB04g20682	RLK
862	BcaB04g20745	RLK
863	BcaB04g20812	RLK
864	BcaB04g20841	RLK
865	BcaB04g20844	RLK
866	BcaB04g20879	RLK

867	BcaB04g20961	RLK
868	BcaB04g21046	RLK
869	BcaB04g21246	RLK
870	BcaB04g21403	RLK
871	BcaB05g21477	RLK
872	BcaB05g21488	RLK
873	BcaB05g21634	RLK
874	BcaB05g21903	RLK
875	BcaB05g21911	RLK
876	BcaB05g21918	RLK
877	BcaB05g22012	RLK
878	BcaB05g22083	RLK
879	BcaB05g22089	RLK
880	BcaB05g22105	RLK
881	BcaB05g22148	RLK
882	BcaB05g22298	RLK
883	BcaB05g22418	RLK
884	BcaB05g22448	RLK
885	BcaB05g22463	RLK
886	BcaB05g22475	RLK
887	BcaB05g22527	RLK
888	BcaB05g22528	RLK
889	BcaB05g22529	RLK
890	BcaB05g22530	RLK
891	BcaB05g22531	RLK
892	BcaB05g22615	RLK
893	BcaB05g22629	RLK
894	BcaB05g22630	RLK
895	BcaB05g22631	RLK
896	BcaB05g22633	RLK
897	BcaB05g22819	RLK
898	BcaB05g22877	RLK
899	BcaB05g23194	RLK
900	BcaB05g23332	RLK
901	BcaB05g23485	RLK
902	BcaB05g23490	RLK
903	BcaB05g23496	RLK
904	BcaB05g23582	RLK
905	BcaB05g23719	RLK
906	BcaB05g23729	RLK
907	BcaB05g23822	RLK
908	BcaB05g23967	RLK
909	BcaB05g24092	RLK
910	BcaB05g24178	RLK
911	BcaB05g24202	RLK
912	BcaB05g24205	RLK
913	BcaB05g24208	RLK
914	BcaB05g24214	RLK
915	BcaB05g24220	RLK
916	BcaB05g24224	RLK
917	BcaB05g24305	RLK
918	BcaB05g24345	RLK
919	BcaB05g24436	RLK
920	BcaB05g24546	RLK
921	BcaB05g24547	RLK
922	BcaB05g24556	RLK
923	BcaB05g24572	RLK
924	BcaB05g24573	RLK
925	BcaB05g24584	RLK
926	BcaB05g24744	RLK
927	BcaB05g24757	RLK
928	BcaB05g24763	RLK

929	BcaB05g25020	RLK
930	BcaB05g25206	RLK
931	BcaB05g25211	RLK
932	BcaB05g25220	RLK
933	BcaB05g25238	RLK
934	BcaB06g25350	RLK
935	BcaB06g25351	RLK
936	BcaB06g25393	RLK
937	BcaB06g25459	RLK
938	BcaB06g25490	RLK
939	BcaB06g25699	RLK
940	BcaB06g25848	RLK
941	BcaB06g25934	RLK
942	BcaB06g26229	RLK
943	BcaB06g26268	RLK
944	BcaB06g26538	RLK
945	BcaB06g26557	RLK
946	BcaB06g26658	RLK
947	BcaB06g26913	RLK
948	BcaB06g27175	RLK
949	BcaB06g27177	RLK
950	BcaB06g27230	RLK
951	BcaB06g27260	RLK
952	BcaB06g27262	RLK
953	BcaB06g27421	RLK
954	BcaB06g27562	RLK
955	BcaB06g27630	RLK
956	BcaB06g27711	RLK
957	BcaB06g27712	RLK
958	BcaB06g27881	RLK
959	BcaB06g27888	RLK
960	BcaB06g27894	RLK
961	BcaB06g27933	RLK
962	BcaB06g27951	RLK
963	BcaB06g28012	RLK
964	BcaB06g28034	RLK
965	BcaB06g28047	RLK
966	BcaB06g28102	RLK
967	BcaB06g28267	RLK
968	BcaB06g28268	RLK
969	BcaB06g28338	RLK
970	BcaB06g28362	RLK
971	BcaB06g28539	RLK
972	BcaB06g28572	RLK
973	BcaB06g28656	RLK
974	BcaB06g28699	RLK
975	BcaB06g28740	RLK
976	BcaB06g28741	RLK
977	BcaB06g28853	RLK
978	BcaB06g28975	RLK
979	BcaB06g29068	RLK
980	BcaB06g29069	RLK
981	BcaB06g29204	RLK
982	BcaB06g29215	RLK
983	BcaB06g29235	RLK
984	BcaB06g29328	RLK
985	BcaB06g29412	RLK
986	BcaB06g29489	RLK
987	BcaB07g29682	RLK
988	BcaB07g29877	RLK
989	BcaB07g30014	RLK
990	BcaB07g30129	RLK

991	BcaB07g30218	RLK
992	BcaB07g30350	RLK
993	BcaB07g30370	RLK
994	BcaB07g30429	RLK
995	BcaB07g30430	RLK
996	BcaB07g30498	RLK
997	BcaB07g30500	RLK
998	BcaB07g30502	RLK
999	BcaB07g30587	RLK
1000	BcaB07g30590	RLK
1001	BcaB07g30604	RLK
1002	BcaB07g30842	RLK
1003	BcaB07g30856	RLK
1004	BcaB07g30925	RLK
1005	BcaB07g31086	RLK
1006	BcaB07g31140	RLK
1007	BcaB07g31273	RLK
1008	BcaB07g31274	RLK
1009	BcaB07g31275	RLK
1010	BcaB07g31276	RLK
1011	BcaB07g31314	RLK
1012	BcaB07g31445	RLK
1013	BcaB07g31621	RLK
1014	BcaB07g31792	RLK
1015	BcaB07g31793	RLK
1016	BcaB07g31826	RLK
1017	BcaB07g31847	RLK
1018	BcaB07g31924	RLK
1019	BcaB07g31946	RLK
1020	BcaB07g31951	RLK
1021	BcaB07g31952	RLK
1022	BcaB07g32113	RLK
1023	BcaB07g32114	RLK
1024	BcaB07g32115	RLK
1025	BcaB07g32395	RLK
1026	BcaB07g32464	RLK
1027	BcaB07g32557	RLK
1028	BcaB07g32626	RLK
1029	BcaB07g32677	RLK
1030	BcaB07g32969	RLK
1031	BcaB07g33135	RLK
1032	BcaB07g33165	RLK
1033	BcaB07g33305	RLK
1034	BcaB07g33653	RLK
1035	BcaB07g33687	RLK
1036	BcaB07g33995	RLK
1037	BcaB07g34001	RLK
1038	BcaB07g34028	RLK
1039	BcaB07g34029	RLK
1040	BcaB07g34039	RLK
1041	BcaB07g34175	RLK
1042	BcaB08g34545	RLK
1043	BcaB08g34637	RLK
1044	BcaB08g34739	RLK
1045	BcaB08g34779	RLK
1046	BcaB08g34842	RLK
1047	BcaB08g34847	RLK
1048	BcaB08g34849	RLK
1049	BcaB08g34903	RLK
1050	BcaB08g35190	RLK
1051	BcaB08g35201	RLK
1052	BcaB08g35202	RLK

1053	BcaB08g35258	RLK
1054	BcaB08g35347	RLK
1055	BcaB08g35348	RLK
1056	BcaB08g35485	RLK
1057	BcaB08g35488	RLK
1058	BcaB08g35510	RLK
1059	BcaB08g35670	RLK
1060	BcaB08g35693	RLK
1061	BcaB08g35790	RLK
1062	BcaB08g35817	RLK
1063	BcaB08g35943	RLK
1064	BcaB08g35968	RLK
1065	BcaB08g36047	RLK
1066	BcaB08g36079	RLK
1067	BcaB08g36176	RLK
1068	BcaB08g36189	RLK
1069	BcaB08g36226	RLK
1070	BcaB08g36231	RLK
1071	BcaB08g36232	RLK
1072	BcaB08g36293	RLK
1073	BcaB08g36294	RLK
1074	BcaB08g36296	RLK
1075	BcaB08g36319	RLK
1076	BcaB08g36320	RLK
1077	BcaB08g36323	RLK
1078	BcaB08g36324	RLK
1079	BcaB08g36325	RLK
1080	BcaB08g36331	RLK
1081	BcaB08g36503	RLK
1082	BcaB08g36519	RLK
1083	BcaB08g36671	RLK
1084	BcaB08g36837	RLK
1085	BcaB08g36966	RLK
1086	BcaB08g37014	RLK
1087	BcaB08g37148	RLK
1088	BcaB08g37241	RLK
1089	BcaB08g37381	RLK
1090	BcaB08g37392	RLK
1091	BcaB08g37393	RLK
1092	BcaB08g37394	RLK
1093	BcaC01g00062	RLK
1094	BcaC01g00086	RLK
1095	BcaC01g00128	RLK
1096	BcaC01g00133	RLK
1097	BcaC01g00180	RLK
1098	BcaC01g00213	RLK
1099	BcaC01g00289	RLK
1100	BcaC01g00559	RLK
1101	BcaC01g00663	RLK
1102	BcaC01g00672	RLK
1103	BcaC01g00728	RLK
1104	BcaC01g00733	RLK
1105	BcaC01g00734	RLK
1106	BcaC01g00735	RLK
1107	BcaC01g00766	RLK
1108	BcaC01g00801	RLK
1109	BcaC01g00802	RLK
1110	BcaC01g00812	RLK
1111	BcaC01g00813	RLK
1112	BcaC01g00989	RLK
1113	BcaC01g01030	RLK
1114	BcaC01g01034	RLK

1115	BcaC01g01123	RLK
1116	BcaC01g01237	RLK
1117	BcaC01g01295	RLK
1118	BcaC01g01318	RLK
1119	BcaC01g01320	RLK
1120	BcaC01g01409	RLK
1121	BcaC01g01411	RLK
1122	BcaC01g01544	RLK
1123	BcaC01g01739	RLK
1124	BcaC01g01801	RLK
1125	BcaC01g01913	RLK
1126	BcaC01g01917	RLK
1127	BcaC01g01969	RLK
1128	BcaC01g01970	RLK
1129	BcaC01g01971	RLK
1130	BcaC01g01973	RLK
1131	BcaC01g02106	RLK
1132	BcaC01g02112	RLK
1133	BcaC01g02165	RLK
1134	BcaC01g02188	RLK
1135	BcaC01g02510	RLK
1136	BcaC01g02629	RLK
1137	BcaC01g02760	RLK
1138	BcaC01g02816	RLK
1139	BcaC01g02841	RLK
1140	BcaC01g02913	RLK
1141	BcaC01g03036	RLK
1142	BcaC01g03115	RLK
1143	BcaC01g03149	RLK
1144	BcaC01g03193	RLK
1145	BcaC01g03262	RLK
1146	BcaC01g03300	RLK
1147	BcaC01g03315	RLK
1148	BcaC01g03391	RLK
1149	BcaC01g03507	RLK
1150	BcaC01g03553	RLK
1151	BcaC01g03575	RLK
1152	BcaC01g03673	RLK
1153	BcaC01g03913	RLK
1154	BcaC01g04392	RLK
1155	BcaC01g04466	RLK
1156	BcaC01g04489	RLK
1157	BcaC01g04499	RLK
1158	BcaC01g04628	RLK
1159	BcaC01g04709	RLK
1160	BcaC01g04752	RLK
1161	BcaC01g04852	RLK
1162	BcaC01g04853	RLK
1163	BcaC01g04874	RLK
1164	BcaC01g04983	RLK
1165	BcaC01g05046	RLK
1166	BcaC01g05056	RLK
1167	BcaC01g05219	RLK
1168	BcaC01g05220	RLK
1169	BcaC01g05281	RLK
1170	BcaC01g05558	RLK
1171	BcaC01g05589	RLK
1172	BcaC01g05598	RLK
1173	BcaC01g05628	RLK
1174	BcaC01g05649	RLK
1175	BcaC01g05650	RLK
1176	BcaC01g05663	RLK

1177	BcaC01g05720	RLK
1178	BcaC01g05990	RLK
1179	BcaC01g05991	RLK
1180	BcaC01g06035	RLK
1181	BcaC01g06138	RLK
1182	BcaC01g06244	RLK
1183	BcaC01g06305	RLK
1184	BcaC01g06314	RLK
1185	BcaC01g06438	RLK
1186	BcaC01g06441	RLK
1187	BcaC01g06503	RLK
1188	BcaC01g06505	RLK
1189	BcaC01g06507	RLK
1190	BcaC01g06526	RLK
1191	BcaC01g06681	RLK
1192	BcaC01g06758	RLK
1193	BcaC01g06759	RLK
1194	BcaC01g06783	RLK
1195	BcaC01g06926	RLK
1196	BcaC02g07030	RLK
1197	BcaC02g07032	RLK
1198	BcaC02g07033	RLK
1199	BcaC02g07080	RLK
1200	BcaC02g07157	RLK
1201	BcaC02g07163	RLK
1202	BcaC02g07252	RLK
1203	BcaC02g07280	RLK
1204	BcaC02g07335	RLK
1205	BcaC02g07339	RLK
1206	BcaC02g07370	RLK
1207	BcaC02g07696	RLK
1208	BcaC02g07794	RLK
1209	BcaC02g07803	RLK
1210	BcaC02g07857	RLK
1211	BcaC02g07960	RLK
1212	BcaC02g08024	RLK
1213	BcaC02g08121	RLK
1214	BcaC02g08166	RLK
1215	BcaC02g08344	RLK
1216	BcaC02g08362	RLK
1217	BcaC02g08375	RLK
1218	BcaC02g08491	RLK
1219	BcaC02g08515	RLK
1220	BcaC02g08522	RLK
1221	BcaC02g08546	RLK
1222	BcaC02g08549	RLK
1223	BcaC02g08557	RLK
1224	BcaC02g08875	RLK
1225	BcaC02g08877	RLK
1226	BcaC02g08978	RLK
1227	BcaC02g09001	RLK
1228	BcaC02g09170	RLK
1229	BcaC02g09297	RLK
1230	BcaC02g09373	RLK
1231	BcaC02g09421	RLK
1232	BcaC02g09434	RLK
1233	BcaC02g09634	RLK
1234	BcaC02g09638	RLK
1235	BcaC02g09639	RLK
1236	BcaC02g09656	RLK
1237	BcaC02g09678	RLK
1238	BcaC02g09679	RLK

1239	BcaC02g09715	RLK
1240	BcaC02g09765	RLK
1241	BcaC02g09800	RLK
1242	BcaC02g09802	RLK
1243	BcaC02g10071	RLK
1244	BcaC02g10077	RLK
1245	BcaC02g10246	RLK
1246	BcaC02g10393	RLK
1247	BcaC02g10577	RLK
1248	BcaC02g10782	RLK
1249	BcaC02g10838	RLK
1250	BcaC02g10855	RLK
1251	BcaC02g10958	RLK
1252	BcaC02g11034	RLK
1253	BcaC02g11035	RLK
1254	BcaC02g11081	RLK
1255	BcaC02g11130	RLK
1256	BcaC02g11181	RLK
1257	BcaC02g11224	RLK
1258	BcaC02g11259	RLK
1259	BcaC02g11273	RLK
1260	BcaC02g11473	RLK
1261	BcaC02g11479	RLK
1262	BcaC02g11588	RLK
1263	BcaC02g11602	RLK
1264	BcaC02g11682	RLK
1265	BcaC02g11832	RLK
1266	BcaC02g11893	RLK
1267	BcaC02g12372	RLK
1268	BcaC02g12490	RLK
1269	BcaC02g12860	RLK
1270	BcaC02g12932	RLK
1271	BcaC03g13083	RLK
1272	BcaC03g13090	RLK
1273	BcaC03g13102	RLK
1274	BcaC03g13175	RLK
1275	BcaC03g13177	RLK
1276	BcaC03g13316	RLK
1277	BcaC03g13405	RLK
1278	BcaC03g13729	RLK
1279	BcaC03g13790	RLK
1280	BcaC03g13983	RLK
1281	BcaC03g13984	RLK
1282	BcaC03g13993	RLK
1283	BcaC03g13994	RLK
1284	BcaC03g13995	RLK
1285	BcaC03g14041	RLK
1286	BcaC03g14043	RLK
1287	BcaC03g14101	RLK
1288	BcaC03g14108	RLK
1289	BcaC03g14198	RLK
1290	BcaC03g14301	RLK
1291	BcaC03g14355	RLK
1292	BcaC03g14363	RLK
1293	BcaC03g14370	RLK
1294	BcaC03g14376	RLK
1295	BcaC03g14456	RLK
1296	BcaC03g14769	RLK
1297	BcaC03g14771	RLK
1298	BcaC03g14781	RLK
1299	BcaC03g14782	RLK
1300	BcaC03g14784	RLK

1301	BcaC03g14862	RLK
1302	BcaC03g15065	RLK
1303	BcaC03g15157	RLK
1304	BcaC03g15175	RLK
1305	BcaC03g15189	RLK
1306	BcaC03g15199	RLK
1307	BcaC03g15368	RLK
1308	BcaC03g15384	RLK
1309	BcaC03g15914	RLK
1310	BcaC03g15916	RLK
1311	BcaC03g15922	RLK
1312	BcaC03g15941	RLK
1313	BcaC03g16018	RLK
1314	BcaC03g16075	RLK
1315	BcaC03g16122	RLK
1316	BcaC03g16237	RLK
1317	BcaC03g16247	RLK
1318	BcaC03g16261	RLK
1319	BcaC03g16513	RLK
1320	BcaC03g16566	RLK
1321	BcaC03g16568	RLK
1322	BcaC03g16690	RLK
1323	BcaC03g16706	RLK
1324	BcaC03g16720	RLK
1325	BcaC03g16788	RLK
1326	BcaC03g16791	RLK
1327	BcaC03g16847	RLK
1328	BcaC03g16928	RLK
1329	BcaC03g17078	RLK
1330	BcaC03g17124	RLK
1331	BcaC03g17131	RLK
1332	BcaC03g17379	RLK
1333	BcaC03g17516	RLK
1334	BcaC03g17597	RLK
1335	BcaC03g17647	RLK
1336	BcaC03g17752	RLK
1337	BcaC03g17823	RLK
1338	BcaC03g17886	RLK
1339	BcaC03g18382	RLK
1340	BcaC03g18387	RLK
1341	BcaC03g18396	RLK
1342	BcaC03g18503	RLK
1343	BcaC03g18522	RLK
1344	BcaC04g18676	RLK
1345	BcaC04g18865	RLK
1346	BcaC04g18917	RLK
1347	BcaC04g19053	RLK
1348	BcaC04g19125	RLK
1349	BcaC04g19178	RLK
1350	BcaC04g19266	RLK
1351	BcaC04g19354	RLK
1352	BcaC04g19435	RLK
1353	BcaC04g19459	RLK
1354	BcaC04g19513	RLK
1355	BcaC04g19623	RLK
1356	BcaC04g19673	RLK
1357	BcaC04g19676	RLK
1358	BcaC04g19681	RLK
1359	BcaC04g19683	RLK
1360	BcaC04g19686	RLK
1361	BcaC04g19691	RLK
1362	BcaC04g19747	RLK

1363	BcaC04g19871	RLK
1364	BcaC04g20045	RLK
1365	BcaC04g20166	RLK
1366	BcaC04g20198	RLK
1367	BcaC04g20324	RLK
1368	BcaC04g20344	RLK
1369	BcaC04g20408	RLK
1370	BcaC04g20413	RLK
1371	BcaC04g20656	RLK
1372	BcaC04g20695	RLK
1373	BcaC04g20707	RLK
1374	BcaC04g20822	RLK
1375	BcaC04g20832	RLK
1376	BcaC04g21144	RLK
1377	BcaC04g21148	RLK
1378	BcaC04g21149	RLK
1379	BcaC04g21294	RLK
1380	BcaC04g21295	RLK
1381	BcaC04g21306	RLK
1382	BcaC04g21356	RLK
1383	BcaC04g21384	RLK
1384	BcaC04g21415	RLK
1385	BcaC04g21710	RLK
1386	BcaC04g21733	RLK
1387	BcaC04g21742	RLK
1388	BcaC04g21806	RLK
1389	BcaC04g21816	RLK
1390	BcaC04g21939	RLK
1391	BcaC04g22007	RLK
1392	BcaC04g22053	RLK
1393	BcaC04g22079	RLK
1394	BcaC04g22327	RLK
1395	BcaC04g22328	RLK
1396	BcaC04g22387	RLK
1397	BcaC04g22539	RLK
1398	BcaC04g22701	RLK
1399	BcaC04g22867	RLK
1400	BcaC04g22904	RLK
1401	BcaC04g23050	RLK
1402	BcaC04g23196	RLK
1403	BcaC04g23252	RLK
1404	BcaC04g23326	RLK
1405	BcaC04g23396	RLK
1406	BcaC04g23455	RLK
1407	BcaC04g23473	RLK
1408	BcaC04g23505	RLK
1409	BcaC04g23522	RLK
1410	BcaC04g23755	RLK
1411	BcaC04g23804	RLK
1412	BcaC04g23885	RLK
1413	BcaC04g23928	RLK
1414	BcaC04g23975	RLK
1415	BcaC04g24003	RLK
1416	BcaC04g24080	RLK
1417	BcaC04g24143	RLK
1418	BcaC04g24181	RLK
1419	BcaC04g24211	RLK
1420	BcaC05g24344	RLK
1421	BcaC05g24638	RLK
1422	BcaC05g24654	RLK
1423	BcaC05g24681	RLK
1424	BcaC05g24688	RLK

1425	BcaC05g24856	RLK
1426	BcaC05g24966	RLK
1427	BcaC05g24993	RLK
1428	BcaC05g24994	RLK
1429	BcaC05g24996	RLK
1430	BcaC05g25008	RLK
1431	BcaC05g25009	RLK
1432	BcaC05g25010	RLK
1433	BcaC05g25017	RLK
1434	BcaC05g25018	RLK
1435	BcaC05g25137	RLK
1436	BcaC05g25188	RLK
1437	BcaC05g25341	RLK
1438	BcaC05g25598	RLK
1439	BcaC05g25654	RLK
1440	BcaC05g25693	RLK
1441	BcaC05g25712	RLK
1442	BcaC05g25732	RLK
1443	BcaC05g25807	RLK
1444	BcaC05g25808	RLK
1445	BcaC05g26029	RLK
1446	BcaC05g26031	RLK
1447	BcaC05g26276	RLK
1448	BcaC05g26336	RLK
1449	BcaC05g26428	RLK
1450	BcaC05g26465	RLK
1451	BcaC05g26468	RLK
1452	BcaC05g26475	RLK
1453	BcaC05g26499	RLK
1454	BcaC05g26642	RLK
1455	BcaC05g26672	RLK
1456	BcaC05g26681	RLK
1457	BcaC05g26862	RLK
1458	BcaC05g26987	RLK
1459	BcaC05g26988	RLK
1460	BcaC05g27056	RLK
1461	BcaC05g27081	RLK
1462	BcaC05g27407	RLK
1463	BcaC05g27712	RLK
1464	BcaC05g27773	RLK
1465	BcaC05g28151	RLK
1466	BcaC05g28477	RLK
1467	BcaC05g28569	RLK
1468	BcaC05g28749	RLK
1469	BcaC05g28751	RLK
1470	BcaC05g28792	RLK
1471	BcaC05g28918	RLK
1472	BcaC05g29019	RLK
1473	BcaC05g29092	RLK
1474	BcaC05g29184	RLK
1475	BcaC05g29214	RLK
1476	BcaC05g29485	RLK
1477	BcaC05g29604	RLK
1478	BcaC05g29701	RLK
1479	BcaC05g29836	RLK
1480	BcaC05g29908	RLK
1481	BcaC05g30066	RLK
1482	BcaC05g30251	RLK
1483	BcaC05g30427	RLK
1484	BcaC06g30649	RLK
1485	BcaC06g30661	RLK
1486	BcaC06g30723	RLK

1487	BcaC06g30761	RLK
1488	BcaC06g30803	RLK
1489	BcaC06g30936	RLK
1490	BcaC06g31048	RLK
1491	BcaC06g31112	RLK
1492	BcaC06g31133	RLK
1493	BcaC06g31217	RLK
1494	BcaC06g31243	RLK
1495	BcaC06g31257	RLK
1496	BcaC06g31275	RLK
1497	BcaC06g31324	RLK
1498	BcaC06g31501	RLK
1499	BcaC06g31510	RLK
1500	BcaC06g31513	RLK
1501	BcaC06g31514	RLK
1502	BcaC06g31516	RLK
1503	BcaC06g31594	RLK
1504	BcaC06g31712	RLK
1505	BcaC06g31731	RLK
1506	BcaC06g31814	RLK
1507	BcaC06g31821	RLK
1508	BcaC06g31835	RLK
1509	BcaC06g31843	RLK
1510	BcaC06g31844	RLK
1511	BcaC06g31852	RLK
1512	BcaC06g31853	RLK
1513	BcaC06g31944	RLK
1514	BcaC06g31958	RLK
1515	BcaC06g32071	RLK
1516	BcaC06g32390	RLK
1517	BcaC06g32398	RLK
1518	BcaC06g32408	RLK
1519	BcaC06g32420	RLK
1520	BcaC06g32518	RLK
1521	BcaC06g32519	RLK
1522	BcaC06g32531	RLK
1523	BcaC06g32539	RLK
1524	BcaC06g32547	RLK
1525	BcaC06g32701	RLK
1526	BcaC06g32814	RLK
1527	BcaC06g33028	RLK
1528	BcaC06g33127	RLK
1529	BcaC06g33153	RLK
1530	BcaC06g33204	RLK
1531	BcaC06g33273	RLK
1532	BcaC06g33492	RLK
1533	BcaC06g33737	RLK
1534	BcaC06g33759	RLK
1535	BcaC06g33981	RLK
1536	BcaC06g34041	RLK
1537	BcaC06g34067	RLK
1538	BcaC06g34092	RLK
1539	BcaC06g34113	RLK
1540	BcaC06g34126	RLK
1541	BcaC06g34176	RLK
1542	BcaC06g34208	RLK
1543	BcaC06g34293	RLK
1544	BcaC06g34381	RLK
1545	BcaC06g34391	RLK
1546	BcaC06g34396	RLK
1547	BcaC06g34397	RLK
1548	BcaC06g34398	RLK

1549	BcaC06g34401	RLK
1550	BcaC06g34521	RLK
1551	BcaC06g34667	RLK
1552	BcaC06g34745	RLK
1553	BcaC06g34802	RLK
1554	BcaC06g34811	RLK
1555	BcaC06g34877	RLK
1556	BcaC06g34878	RLK
1557	BcaC06g35216	RLK
1558	BcaC06g35243	RLK
1559	BcaC06g35370	RLK
1560	BcaC06g35453	RLK
1561	BcaC06g35460	RLK
1562	BcaC06g35462	RLK
1563	BcaC06g35466	RLK
1564	BcaC06g35498	RLK
1565	BcaC06g35564	RLK
1566	BcaC06g35592	RLK
1567	BcaC06g35721	RLK
1568	BcaC06g35978	RLK
1569	BcaC06g35998	RLK
1570	BcaC06g36133	RLK
1571	BcaC06g36154	RLK
1572	BcaC06g36251	RLK
1573	BcaC06g36300	RLK
1574	BcaC06g36415	RLK
1575	BcaC06g36416	RLK
1576	BcaC06g36713	RLK
1577	BcaC07g36844	RLK
1578	BcaC07g36871	RLK
1579	BcaC07g37132	RLK
1580	BcaC07g37539	RLK
1581	BcaC07g37666	RLK
1582	BcaC07g38129	RLK
1583	BcaC07g38326	RLK
1584	BcaC07g38374	RLK
1585	BcaC07g38402	RLK
1586	BcaC07g38566	RLK
1587	BcaC07g38578	RLK
1588	BcaC07g38595	RLK
1589	BcaC07g38615	RLK
1590	BcaC07g38653	RLK
1591	BcaC07g38658	RLK
1592	BcaC07g38664	RLK
1593	BcaC07g38870	RLK
1594	BcaC07g39085	RLK
1595	BcaC07g39086	RLK
1596	BcaC07g39088	RLK
1597	BcaC07g39089	RLK
1598	BcaC07g39150	RLK
1599	BcaC07g39204	RLK
1600	BcaC07g39722	RLK
1601	BcaC07g39738	RLK
1602	BcaC07g39912	RLK
1603	BcaC07g39929	RLK
1604	BcaC07g39951	RLK
1605	BcaC07g40151	RLK
1606	BcaC07g40159	RLK
1607	BcaC07g40217	RLK
1608	BcaC07g40286	RLK
1609	BcaC07g40335	RLK
1610	BcaC07g40826	RLK

1611	BcaC07g40854	RLK
1612	BcaC07g40861	RLK
1613	BcaC07g40936	RLK
1614	BcaC07g40957	RLK
1615	BcaC07g40984	RLK
1616	BcaC07g40996	RLK
1617	BcaC07g41092	RLK
1618	BcaC07g41221	RLK
1619	BcaC07g41332	RLK
1620	BcaC07g41495	RLK
1621	BcaC07g41783	RLK
1622	BcaC07g41784	RLK
1623	BcaC07g41785	RLK
1624	BcaC07g41792	RLK
1625	BcaC07g41850	RLK
1626	BcaC07g41986	RLK
1627	BcaC07g42355	RLK
1628	BcaC07g42423	RLK
1629	BcaC07g42484	RLK
1630	BcaC07g42609	RLK
1631	BcaC07g42616	RLK
1632	BcaC07g42617	RLK
1633	BcaC08g43124	RLK
1634	BcaC08g43158	RLK
1635	BcaC08g43330	RLK
1636	BcaC08g43349	RLK
1637	BcaC08g43350	RLK
1638	BcaC08g43353	RLK
1639	BcaC08g43354	RLK
1640	BcaC08g43552	RLK
1641	BcaC08g43680	RLK
1642	BcaC08g43788	RLK
1643	BcaC08g43872	RLK
1644	BcaC08g43940	RLK
1645	BcaC08g43942	RLK
1646	BcaC08g43943	RLK
1647	BcaC08g43946	RLK
1648	BcaC08g43948	RLK
1649	BcaC08g44092	RLK
1650	BcaC08g44370	RLK
1651	BcaC08g44409	RLK
1652	BcaC08g44452	RLK
1653	BcaC08g44512	RLK
1654	BcaC08g44741	RLK
1655	BcaC08g44760	RLK
1656	BcaC08g44912	RLK
1657	BcaC08g44938	RLK
1658	BcaC08g44959	RLK
1659	BcaC08g45001	RLK
1660	BcaC08g45675	RLK
1661	BcaC08g45708	RLK
1662	BcaC08g45722	RLK
1663	BcaC08g45754	RLK
1664	BcaC08g45843	RLK
1665	BcaC08g45855	RLK
1666	BcaC08g45936	RLK
1667	BcaC08g45945	RLK
1668	BcaC08g46076	RLK
1669	BcaC08g46098	RLK
1670	BcaC08g46099	RLK
1671	BcaC08g46117	RLK
1672	BcaC08g46118	RLK

1673	BcaC08g46123	RLK
1674	BcaC08g46124	RLK
1675	BcaC08g46125	RLK
1676	BcaC08g46130	RLK
1677	BcaC08g46156	RLK
1678	BcaC08g46192	RLK
1679	BcaC08g46193	RLK
1680	BcaC08g46321	RLK
1681	BcaC08g46449	RLK
1682	BcaC08g46461	RLK
1683	BcaC08g46462	RLK
1684	BcaC08g46488	RLK
1685	BcaC08g46501	RLK
1686	BcaC08g46502	RLK
1687	BcaC08g46614	RLK
1688	BcaC08g46665	RLK
1689	BcaC08g46685	RLK
1690	BcaC08g46786	RLK
1691	BcaC08g46866	RLK
1692	BcaC08g46900	RLK
1693	BcaC08g47016	RLK
1694	BcaC08g47070	RLK
1695	BcaC08g47324	RLK
1696	BcaC08g47355	RLK
1697	BcaC08g47436	RLK
1698	BcaC08g47526	RLK
1699	BcaC08g47527	RLK
1700	BcaC09g47686	RLK
1701	BcaC09g47996	RLK
1702	BcaC09g48037	RLK
1703	BcaC09g48039	RLK
1704	BcaC09g48404	RLK
1705	BcaC09g48434	RLK
1706	BcaC09g48450	RLK
1707	BcaC09g48451	RLK
1708	BcaC09g48704	RLK
1709	BcaC09g48745	RLK
1710	BcaC09g48850	RLK
1711	BcaC09g48931	RLK
1712	BcaC09g48968	RLK
1713	BcaC09g48995	RLK
1714	BcaC09g49368	RLK
1715	BcaC09g49736	RLK
1716	BcaC09g49738	RLK
1717	BcaC09g49741	RLK
1718	BcaC09g50018	RLK
1719	BcaC09g50102	RLK
1720	BcaC09g50352	RLK
1721	BcaC09g50588	RLK
1722	BcaC09g50589	RLK
1723	BcaC09g50653	RLK
1724	BcaC09g50950	RLK
1725	BcaC09g51013	RLK
1726	BcaC09g51014	RLK
1727	BcaC09g51016	RLK
1728	BcaC09g51017	RLK
1729	BcaC09g51018	RLK
1730	BcaC09g51019	RLK
1731	BcaC09g51021	RLK
1732	BcaC09g51022	RLK
1733	BcaC09g51023	RLK
1734	BcaC09g51024	RLK

1735	BcaC09g51025	RLK
1736	BcaC09g51059	RLK
1737	BcaC09g51061	RLK
1738	BcaC09g51094	RLK
1739	BcaC09g51159	RLK
1740	BcaC09g51178	RLK
1741	BcaC09g51230	RLK
1742	BcaC09g51231	RLK
1743	BcaC09g51232	RLK
1744	BcaC09g51290	RLK
1745	BcaC09g51309	RLK
1746	BcaC09g51325	RLK
1747	BcaC09g51468	RLK
1748	BcaC09g51540	RLK
1749	BcaC09g51541	RLK
1750	BcaC09g51651	RLK
1751	BcaC09g51685	RLK
1752	BcaC09g51731	RLK
1753	BcaC09g51732	RLK
1754	BcaC09g51763	RLK
1755	BcaC09g51777	RLK
1756	BcaC09g51849	RLK
1757	BcaC09g51882	RLK
1758	BcaC09g51883	RLK
1759	BcaC09g51884	RLK
1760	BcaC09g51893	RLK
1761	BcaC09g51894	RLK
1762	BcaC09g51904	RLK
1763	BcaC09g51988	RLK
1764	BcaC09g52006	RLK
1765	BcaC09g52013	RLK
1766	BcaC09g52014	RLK
1767	BcaC09g52064	RLK
1768	BcaC09g52155	RLK
1769	BcaNung00373	RLK
1770	BcaNung00455	RLK
1771	BcaNung00563	RLK
1772	BcaNung00752	RLK
1773	BcaNung00824	RLK
1774	BcaNung00859	RLK
1775	BcaNung00930	RLK
1776	BcaNung00986	RLK
1777	BcaNung00989	RLK
1778	BcaNung00990	RLK
1779	BcaNung00992	RLK
1780	BcaNung00993	RLK
1781	BcaNung00994	RLK
1782	BcaNung01002	RLK
1783	BcaNung01012	RLK
1784	BcaNung01013	RLK
1785	BcaNung01014	RLK
1786	BcaNung01015	RLK
1787	BcaNung01016	RLK
1788	BcaNung01041	RLK
1789	BcaNung01073	RLK
1790	BcaNung01083	RLK
1791	BcaNung01165	RLK
1792	BcaNung01200	RLK
1793	BcaNung01207	RLK
1794	BcaNung01227	RLK
1795	BcaNung01279	RLK
1796	BcaNung01335	RLK

1797	BcaNung01451	RLK
1798	BcaNung01452	RLK
1799	BcaNung01458	RLK
1800	BcaNung01459	RLK
1801	BcaNung01460	RLK
1802	BcaNung01461	RLK
1803	BcaNung01463	RLK
1804	BcaNung01464	RLK
1805	BcaNung01465	RLK
1806	BcaNung01466	RLK
1807	BcaNung01469	RLK
1808	BcaNung01470	RLK
1809	BcaNung01551	RLK
1810	BcaNung01675	RLK
1811	BcaNung01722	RLK
1812	BcaNung01823	RLK
1813	BcaNung01830	RLK
1814	BcaNung01876	RLK
1815	BcaNung01965	RLK
1816	BcaNung02038	RLK
1817	BcaNung02107	RLK
1818	BcaNung02172	RLK
1819	BcaNung02295	RLK
1820	BcaNung02339	RLK
1821	BcaNung02344	RLK
1822	BcaNung02387	RLK
1823	BcaNung02405	RLK
1824	BcaNung02531	RLK
1825	BcaNung02608	RLK
1826	BcaNung02724	RLK
1827	BcaNung02731	RLK
1828	BcaNung02802	RLK
1829	BcaNung02809	RLK
1830	BcaNung02839	RLK
1831	BcaNung02866	RLK
1832	BcaNung02978	RLK
1833	BcaNung03045	RLK
1834	BcaNung03046	RLK
1835	BcaNung03062	RLK
1836	BcaNung03128	RLK
1837	BcaNung03145	RLK
1838	BcaNung03180	RLK
1839	BcaNung03189	RLK
1840	BcaNung03201	RLK
1841	BcaNung03253	RLK
1842	BcaNung03255	RLK
1843	BcaNung03256	RLK
1844	BcaNung03257	RLK
1845	BcaNung03258	RLK
1846	BcaNung03260	RLK
1847	BcaNung03261	RLK
1848	BcaNung03263	RLK
1849	BcaNung03414	RLK
1850	BcaNung03415	RLK
1851	BcaNung03417	RLK
1852	BcaNung03425	RLK
1853	BcaNung03442	RLK
1854	BcaNung03498	RLK
1855	BcaNung03587	RLK
1856	BcaNung03761	RLK
1857	BcaNung03805	RLK
1858	BcaNung03880	RLK

1859	BcaNung04051	RLK
1860	BcaNung04052	RLK
1861	BcaNung04152	RLK
1862	BcaNung04172	RLK
1863	BcaNung04197	RLK
1864	BcaNung04198	RLK
1865	BcaNung04293	RLK
1866	BcaNung04313	RLK
1867	BcaNung04453	RLK
1868	BcaNung04548	RLK
1869	BcaNung04695	RLK
1870	BcaNung04697	RLK
1871	BcaNung04716	RLK
1872	BcaNung04758	RLK
1873	BcaNung04794	RLK
1874	BcaNung04948	RLK
1875	BcaNung05059	RLK
1876	BcaNung05061	RLK
1877	BcaNung05121	RLK
1878	BcaNung05256	RLK
1879	BcaNung05257	RLK
1880	BcaNung05294	RLK
1881	BcaNung05315	RLK
1882	BcaNung05431	RLK
1883	BcaNung05502	RLK
1884	BcaNung05598	RLK
1885	BcaNung05672	RLK
1886	BcaNung05682	RLK
1887	BcaNung05819	RLK
1888	BcaNung05995	RLK
1889	BcaNung06086	RLK
1890	BcaNung06261	RLK
1891	BcaNung06286	RLK
1892	BcaNung06417	RLK
1893	BcaNung06590	RLK
1894	BcaNung06704	RLK
1895	BcaNung06807	RLK
1896	BcaNung06919	RLK
1897	BcaNung06943	RLK
1898	BcaNung07049	RLK
1899	BcaNung07108	RLK
1900	BcaNung07267	RLK
1901	BcaNung07268	RLK
1902	BcaNung07371	RLK
1903	BcaB01g00056	RLP
1904	BcaB01g00093	RLP
1905	BcaB01g00418	RLP
1906	BcaB01g00676	RLP
1907	BcaB01g00878	RLP
1908	BcaB01g02395	RLP
1909	BcaB01g02397	RLP
1910	BcaB01g02406	RLP
1911	BcaB01g02901	RLP
1912	BcaB01g03008	RLP
1913	BcaB01g03827	RLP
1914	BcaB01g04059	RLP
1915	BcaB01g04582	RLP
1916	BcaB01g05251	RLP
1917	BcaB01g05876	RLP
1918	BcaB01g06010	RLP
1919	BcaB01g06403	RLP
1920	BcaB02g06676	RLP

1921	BcaB02g07056	RLP
1922	BcaB02g07296	RLP
1923	BcaB02g07607	RLP
1924	BcaB02g07799	RLP
1925	BcaB02g08646	RLP
1926	BcaB02g08649	RLP
1927	BcaB02g10496	RLP
1928	BcaB02g10521	RLP
1929	BcaB02g10522	RLP
1930	BcaB02g10542	RLP
1931	BcaB02g11384	RLP
1932	BcaB02g11438	RLP
1933	BcaB03g13011	RLP
1934	BcaB03g14600	RLP
1935	BcaB03g14696	RLP
1936	BcaB03g14898	RLP
1937	BcaB03g14899	RLP
1938	BcaB03g15370	RLP
1939	BcaB03g15377	RLP
1940	BcaB03g15604	RLP
1941	BcaB03g15606	RLP
1942	BcaB03g16661	RLP
1943	BcaB03g16713	RLP
1944	BcaB04g17640	RLP
1945	BcaB04g17911	RLP
1946	BcaB04g17947	RLP
1947	BcaB04g18455	RLP
1948	BcaB04g18487	RLP
1949	BcaB04g18496	RLP
1950	BcaB04g18620	RLP
1951	BcaB04g18720	RLP
1952	BcaB04g19351	RLP
1953	BcaB04g19411	RLP
1954	BcaB04g20032	RLP
1955	BcaB04g20190	RLP
1956	BcaB04g20561	RLP
1957	BcaB04g20959	RLP
1958	BcaB04g21147	RLP
1959	BcaB04g21153	RLP
1960	BcaB04g21157	RLP
1961	BcaB05g21858	RLP
1962	BcaB05g23124	RLP
1963	BcaB05g23149	RLP
1964	BcaB05g24110	RLP
1965	BcaB05g24656	RLP
1966	BcaB05g24670	RLP
1967	BcaB05g24671	RLP
1968	BcaB05g24837	RLP
1969	BcaB05g24838	RLP
1970	BcaB05g24839	RLP
1971	BcaB05g24841	RLP
1972	BcaB05g24842	RLP
1973	BcaB05g25064	RLP
1974	BcaB05g25181	RLP
1975	BcaB06g26446	RLP
1976	BcaB06g26536	RLP
1977	BcaB06g27884	RLP
1978	BcaB06g28018	RLP
1979	BcaB06g28060	RLP
1980	BcaB06g28266	RLP
1981	BcaB06g28624	RLP
1982	BcaB06g28673	RLP

1983	BcaB06g28933	RLP
1984	BcaB06g29240	RLP
1985	BcaB06g29404	RLP
1986	BcaB07g29977	RLP
1987	BcaB07g30013	RLP
1988	BcaB07g30126	RLP
1989	BcaB07g30482	RLP
1990	BcaB07g30725	RLP
1991	BcaB07g30869	RLP
1992	BcaB07g31134	RLP
1993	BcaB07g31836	RLP
1994	BcaB07g31878	RLP
1995	BcaB07g31881	RLP
1996	BcaB07g31882	RLP
1997	BcaB07g32183	RLP
1998	BcaB07g32337	RLP
1999	BcaB07g32338	RLP
2000	BcaB07g32450	RLP
2001	BcaB07g32846	RLP
2002	BcaB07g32911	RLP
2003	BcaB07g34037	RLP
2004	BcaB08g34403	RLP
2005	BcaB08g34433	RLP
2006	BcaB08g34437	RLP
2007	BcaB08g34570	RLP
2008	BcaB08g35168	RLP
2009	BcaB08g35731	RLP
2010	BcaB08g36824	RLP
2011	BcaB08g36876	RLP
2012	BcaC01g00553	RLP
2013	BcaC01g00554	RLP
2014	BcaC01g00565	RLP
2015	BcaC01g01407	RLP
2016	BcaC01g01646	RLP
2017	BcaC01g03088	RLP
2018	BcaC01g03245	RLP
2019	BcaC01g03345	RLP
2020	BcaC01g03463	RLP
2021	BcaC01g03567	RLP
2022	BcaC01g04278	RLP
2023	BcaC01g04816	RLP
2024	BcaC01g04817	RLP
2025	BcaC01g04901	RLP
2026	BcaC01g05860	RLP
2027	BcaC01g05928	RLP
2028	BcaC01g06522	RLP
2029	BcaC02g07120	RLP
2030	BcaC02g07787	RLP
2031	BcaC02g07848	RLP
2032	BcaC02g07894	RLP
2033	BcaC02g08186	RLP
2034	BcaC02g08350	RLP
2035	BcaC02g08373	RLP
2036	BcaC02g08634	RLP
2037	BcaC02g09153	RLP
2038	BcaC02g09337	RLP
2039	BcaC02g09381	RLP
2040	BcaC02g09387	RLP
2041	BcaC02g11349	RLP
2042	BcaC02g11370	RLP
2043	BcaC02g11504	RLP
2044	BcaC03g13897	RLP

2045	BcaC03g14695	RLP
2046	BcaC03g14697	RLP
2047	BcaC03g15293	RLP
2048	BcaC03g15487	RLP
2049	BcaC03g15490	RLP
2050	BcaC03g15755	RLP
2051	BcaC03g16859	RLP
2052	BcaC03g17646	RLP
2053	BcaC03g18510	RLP
2054	BcaC04g21745	RLP
2055	BcaC04g22095	RLP
2056	BcaC04g22543	RLP
2057	BcaC04g22948	RLP
2058	BcaC04g23205	RLP
2059	BcaC04g23245	RLP
2060	BcaC04g23512	RLP
2061	BcaC04g23871	RLP
2062	BcaC05g24412	RLP
2063	BcaC05g24568	RLP
2064	BcaC05g24569	RLP
2065	BcaC05g24872	RLP
2066	BcaC05g24932	RLP
2067	BcaC05g25653	RLP
2068	BcaC05g25698	RLP
2069	BcaC05g25757	RLP
2070	BcaC05g26105	RLP
2071	BcaC05g26263	RLP
2072	BcaC05g26437	RLP
2073	BcaC05g26651	RLP
2074	BcaC05g26843	RLP
2075	BcaC05g26990	RLP
2076	BcaC05g29053	RLP
2077	BcaC05g29054	RLP
2078	BcaC06g30593	RLP
2079	BcaC06g30915	RLP
2080	BcaC06g31456	RLP
2081	BcaC06g31469	RLP
2082	BcaC06g32224	RLP
2083	BcaC06g32455	RLP
2084	BcaC06g32468	RLP
2085	BcaC06g32472	RLP
2086	BcaC06g33221	RLP
2087	BcaC06g33362	RLP
2088	BcaC06g34468	RLP
2089	BcaC06g36310	RLP
2090	BcaC07g37916	RLP
2091	BcaC07g38114	RLP
2092	BcaC07g38494	RLP
2093	BcaC07g38607	RLP
2094	BcaC07g38838	RLP
2095	BcaC07g39438	RLP
2096	BcaC07g39907	RLP
2097	BcaC07g40441	RLP
2098	BcaC07g40476	RLP
2099	BcaC07g41409	RLP
2100	BcaC07g41431	RLP
2101	BcaC07g41565	RLP
2102	BcaC07g41622	RLP
2103	BcaC07g42003	RLP
2104	BcaC07g42004	RLP
2105	BcaC07g42005	RLP
2106	BcaC07g42006	RLP

2107	BcaC07g42153	RLP
2108	BcaC07g42463	RLP
2109	BcaC07g42465	RLP
2110	BcaC08g43816	RLP
2111	BcaC08g43945	RLP
2112	BcaC08g44826	RLP
2113	BcaC08g45291	RLP
2114	BcaC08g45538	RLP
2115	BcaC08g45603	RLP
2116	BcaC08g45607	RLP
2117	BcaC08g45777	RLP
2118	BcaC08g46033	RLP
2119	BcaC08g46034	RLP
2120	BcaC08g46574	RLP
2121	BcaC08g46575	RLP
2122	BcaC08g46821	RLP
2123	BcaC08g46822	RLP
2124	BcaC08g46848	RLP
2125	BcaC09g49504	RLP
2126	BcaC09g49968	RLP
2127	BcaC09g50127	RLP
2128	BcaC09g50129	RLP
2129	BcaC09g51161	RLP
2130	BcaC09g51473	RLP
2131	BcaC09g51655	RLP
2132	BcaC09g51905	RLP
2133	BcaC09g52054	RLP
2134	BcaNung00356	RLP
2135	BcaNung00359	RLP
2136	BcaNung00360	RLP
2137	BcaNung00714	RLP
2138	BcaNung02642	RLP
2139	BcaNung04289	RLP
2140	BcaNung04550	RLP
2141	BcaNung04688	RLP
2142	BcaNung04728	RLP
2143	BcaNung05501	RLP
2144	BcaNung05916	RLP
2145	BcaNung05943	RLP
2146	BcaB01g00219	TM-CC
2147	BcaB01g00271	TM-CC
2148	BcaB01g00308	TM-CC
2149	BcaB01g00312	TM-CC
2150	BcaB01g00549	TM-CC
2151	BcaB01g00612	TM-CC
2152	BcaB01g00638	TM-CC
2153	BcaB01g00921	TM-CC
2154	BcaB01g01628	TM-CC
2155	BcaB01g01730	TM-CC
2156	BcaB01g02162	TM-CC
2157	BcaB01g02172	TM-CC
2158	BcaB01g02365	TM-CC
2159	BcaB01g02369	TM-CC
2160	BcaB01g02593	TM-CC
2161	BcaB01g02644	TM-CC
2162	BcaB01g03051	TM-CC
2163	BcaB01g03289	TM-CC
2164	BcaB01g03290	TM-CC
2165	BcaB01g03953	TM-CC
2166	BcaB01g04671	TM-CC
2167	BcaB01g04773	TM-CC
2168	BcaB01g04792	TM-CC

2169	BcaB01g04936	TM-CC
2170	BcaB01g05092	TM-CC
2171	BcaB01g05203	TM-CC
2172	BcaB01g05735	TM-CC
2173	BcaB01g05921	TM-CC
2174	BcaB01g05984	TM-CC
2175	BcaB01g06056	TM-CC
2176	BcaB01g06142	TM-CC
2177	BcaB02g06783	TM-CC
2178	BcaB02g06896	TM-CC
2179	BcaB02g06918	TM-CC
2180	BcaB02g07392	TM-CC
2181	BcaB02g07403	TM-CC
2182	BcaB02g07416	TM-CC
2183	BcaB02g07495	TM-CC
2184	BcaB02g08207	TM-CC
2185	BcaB02g08442	TM-CC
2186	BcaB02g09115	TM-CC
2187	BcaB02g09310	TM-CC
2188	BcaB02g09329	TM-CC
2189	BcaB02g09353	TM-CC
2190	BcaB02g09561	TM-CC
2191	BcaB02g09643	TM-CC
2192	BcaB02g09653	TM-CC
2193	BcaB02g09796	TM-CC
2194	BcaB02g09819	TM-CC
2195	BcaB02g09843	TM-CC
2196	BcaB02g10082	TM-CC
2197	BcaB03g11764	TM-CC
2198	BcaB03g12711	TM-CC
2199	BcaB03g12815	TM-CC
2200	BcaB03g12888	TM-CC
2201	BcaB03g13033	TM-CC
2202	BcaB03g13263	TM-CC
2203	BcaB03g13771	TM-CC
2204	BcaB03g14321	TM-CC
2205	BcaB03g14393	TM-CC
2206	BcaB03g14716	TM-CC
2207	BcaB03g14878	TM-CC
2208	BcaB03g15031	TM-CC
2209	BcaB03g15332	TM-CC
2210	BcaB03g15960	TM-CC
2211	BcaB03g16036	TM-CC
2212	BcaB03g16146	TM-CC
2213	BcaB03g16416	TM-CC
2214	BcaB03g16631	TM-CC
2215	BcaB04g17096	TM-CC
2216	BcaB04g17891	TM-CC
2217	BcaB04g18002	TM-CC
2218	BcaB04g18072	TM-CC
2219	BcaB04g18367	TM-CC
2220	BcaB04g18518	TM-CC
2221	BcaB04g18667	TM-CC
2222	BcaB04g18869	TM-CC
2223	BcaB04g19137	TM-CC
2224	BcaB04g19461	TM-CC
2225	BcaB04g19466	TM-CC
2226	BcaB04g19810	TM-CC
2227	BcaB04g19811	TM-CC
2228	BcaB04g19990	TM-CC
2229	BcaB04g20114	TM-CC
2230	BcaB04g20166	TM-CC

2231	BcaB04g20230	TM-CC
2232	BcaB04g20329	TM-CC
2233	BcaB04g20337	TM-CC
2234	BcaB04g20473	TM-CC
2235	BcaB05g21599	TM-CC
2236	BcaB05g21641	TM-CC
2237	BcaB05g21728	TM-CC
2238	BcaB05g21742	TM-CC
2239	BcaB05g22020	TM-CC
2240	BcaB05g22145	TM-CC
2241	BcaB05g22170	TM-CC
2242	BcaB05g22405	TM-CC
2243	BcaB05g22738	TM-CC
2244	BcaB05g24075	TM-CC
2245	BcaB05g24144	TM-CC
2246	BcaB05g24166	TM-CC
2247	BcaB05g24908	TM-CC
2248	BcaB05g25320	TM-CC
2249	BcaB06g25429	TM-CC
2250	BcaB06g25494	TM-CC
2251	BcaB06g25608	TM-CC
2252	BcaB06g25837	TM-CC
2253	BcaB06g25889	TM-CC
2254	BcaB06g26096	TM-CC
2255	BcaB06g26113	TM-CC
2256	BcaB06g26114	TM-CC
2257	BcaB06g26271	TM-CC
2258	BcaB06g26494	TM-CC
2259	BcaB06g26748	TM-CC
2260	BcaB06g27534	TM-CC
2261	BcaB06g28084	TM-CC
2262	BcaB06g28252	TM-CC
2263	BcaB06g28622	TM-CC
2264	BcaB06g28868	TM-CC
2265	BcaB06g28870	TM-CC
2266	BcaB06g28985	TM-CC
2267	BcaB06g29306	TM-CC
2268	BcaB07g29755	TM-CC
2269	BcaB07g29855	TM-CC
2270	BcaB07g29935	TM-CC
2271	BcaB07g29939	TM-CC
2272	BcaB07g30661	TM-CC
2273	BcaB07g30776	TM-CC
2274	BcaB07g30789	TM-CC
2275	BcaB07g30832	TM-CC
2276	BcaB07g30861	TM-CC
2277	BcaB07g30920	TM-CC
2278	BcaB07g31268	TM-CC
2279	BcaB07g31397	TM-CC
2280	BcaB07g31536	TM-CC
2281	BcaB07g31565	TM-CC
2282	BcaB07g31602	TM-CC
2283	BcaB07g31663	TM-CC
2284	BcaB07g31730	TM-CC
2285	BcaB07g31841	TM-CC
2286	BcaB07g31945	TM-CC
2287	BcaB07g32404	TM-CC
2288	BcaB07g32721	TM-CC
2289	BcaB07g33207	TM-CC
2290	BcaB07g33875	TM-CC
2291	BcaB08g34456	TM-CC
2292	BcaB08g34855	TM-CC

2293	BcaB08g35560	TM-CC
2294	BcaB08g35680	TM-CC
2295	BcaB08g35758	TM-CC
2296	BcaB08g36167	TM-CC
2297	BcaB08g36492	TM-CC
2298	BcaB08g36518	TM-CC
2299	BcaB08g36545	TM-CC
2300	BcaB08g36589	TM-CC
2301	BcaB08g36937	TM-CC
2302	BcaB08g37094	TM-CC
2303	BcaB08g37095	TM-CC
2304	BcaB08g37449	TM-CC
2305	BcaC01g00169	TM-CC
2306	BcaC01g00171	TM-CC
2307	BcaC01g00337	TM-CC
2308	BcaC01g00819	TM-CC
2309	BcaC01g00982	TM-CC
2310	BcaC01g01373	TM-CC
2311	BcaC01g01822	TM-CC
2312	BcaC01g01853	TM-CC
2313	BcaC01g02250	TM-CC
2314	BcaC01g02297	TM-CC
2315	BcaC01g02332	TM-CC
2316	BcaC01g02465	TM-CC
2317	BcaC01g02657	TM-CC
2318	BcaC01g02686	TM-CC
2319	BcaC01g02705	TM-CC
2320	BcaC01g02725	TM-CC
2321	BcaC01g02987	TM-CC
2322	BcaC01g03222	TM-CC
2323	BcaC01g03719	TM-CC
2324	BcaC01g03970	TM-CC
2325	BcaC01g04189	TM-CC
2326	BcaC01g04268	TM-CC
2327	BcaC01g04863	TM-CC
2328	BcaC01g04924	TM-CC
2329	BcaC01g05156	TM-CC
2330	BcaC01g05876	TM-CC
2331	BcaC01g06641	TM-CC
2332	BcaC01g06877	TM-CC
2333	BcaC01g06885	TM-CC
2334	BcaC02g07188	TM-CC
2335	BcaC02g07219	TM-CC
2336	BcaC02g07673	TM-CC
2337	BcaC02g08333	TM-CC
2338	BcaC02g09020	TM-CC
2339	BcaC02g09259	TM-CC
2340	BcaC02g09266	TM-CC
2341	BcaC02g09470	TM-CC
2342	BcaC02g09906	TM-CC
2343	BcaC02g10050	TM-CC
2344	BcaC02g10089	TM-CC
2345	BcaC02g10163	TM-CC
2346	BcaC02g10201	TM-CC
2347	BcaC02g10580	TM-CC
2348	BcaC02g10867	TM-CC
2349	BcaC02g11017	TM-CC
2350	BcaC02g11496	TM-CC
2351	BcaC02g11826	TM-CC
2352	BcaC03g12936	TM-CC
2353	BcaC03g13137	TM-CC
2354	BcaC03g13149	TM-CC

2355	BcaC03g13264	TM-CC
2356	BcaC03g13872	TM-CC
2357	BcaC03g14113	TM-CC
2358	BcaC03g14448	TM-CC
2359	BcaC03g14493	TM-CC
2360	BcaC03g14593	TM-CC
2361	BcaC03g15111	TM-CC
2362	BcaC03g15169	TM-CC
2363	BcaC03g15359	TM-CC
2364	BcaC03g15576	TM-CC
2365	BcaC03g15832	TM-CC
2366	BcaC03g16948	TM-CC
2367	BcaC03g17279	TM-CC
2368	BcaC03g17490	TM-CC
2369	BcaC03g17498	TM-CC
2370	BcaC03g17514	TM-CC
2371	BcaC03g17521	TM-CC
2372	BcaC03g17673	TM-CC
2373	BcaC03g17925	TM-CC
2374	BcaC03g18008	TM-CC
2375	BcaC03g18155	TM-CC
2376	BcaC03g18160	TM-CC
2377	BcaC03g18274	TM-CC
2378	BcaC03g18341	TM-CC
2379	BcaC03g18370	TM-CC
2380	BcaC03g18416	TM-CC
2381	BcaC03g18422	TM-CC
2382	BcaC03g18481	TM-CC
2383	BcaC04g18924	TM-CC
2384	BcaC04g18999	TM-CC
2385	BcaC04g19406	TM-CC
2386	BcaC04g19736	TM-CC
2387	BcaC04g19903	TM-CC
2388	BcaC04g20325	TM-CC
2389	BcaC04g20395	TM-CC
2390	BcaC04g20491	TM-CC
2391	BcaC04g20608	TM-CC
2392	BcaC04g20925	TM-CC
2393	BcaC04g21128	TM-CC
2394	BcaC04g21292	TM-CC
2395	BcaC04g21629	TM-CC
2396	BcaC04g21642	TM-CC
2397	BcaC04g21855	TM-CC
2398	BcaC04g22142	TM-CC
2399	BcaC04g22249	TM-CC
2400	BcaC04g22317	TM-CC
2401	BcaC04g22408	TM-CC
2402	BcaC04g22482	TM-CC
2403	BcaC04g22667	TM-CC
2404	BcaC04g22838	TM-CC
2405	BcaC04g22901	TM-CC
2406	BcaC04g22974	TM-CC
2407	BcaC04g23138	TM-CC
2408	BcaC04g23144	TM-CC
2409	BcaC04g23289	TM-CC
2410	BcaC04g23572	TM-CC
2411	BcaC04g23591	TM-CC
2412	BcaC04g23853	TM-CC
2413	BcaC04g23872	TM-CC
2414	BcaC05g24468	TM-CC
2415	BcaC05g24522	TM-CC
2416	BcaC05g24984	TM-CC

2417	BcaC05g25826	TM-CC
2418	BcaC05g26316	TM-CC
2419	BcaC05g26379	TM-CC
2420	BcaC05g26971	TM-CC
2421	BcaC05g27409	TM-CC
2422	BcaC05g27933	TM-CC
2423	BcaC05g27976	TM-CC
2424	BcaC05g28333	TM-CC
2425	BcaC05g28335	TM-CC
2426	BcaC05g28360	TM-CC
2427	BcaC05g28897	TM-CC
2428	BcaC05g29086	TM-CC
2429	BcaC05g29152	TM-CC
2430	BcaC05g29218	TM-CC
2431	BcaC05g29355	TM-CC
2432	BcaC05g29423	TM-CC
2433	BcaC05g29438	TM-CC
2434	BcaC05g29690	TM-CC
2435	BcaC05g29949	TM-CC
2436	BcaC05g30119	TM-CC
2437	BcaC05g30199	TM-CC
2438	BcaC05g30203	TM-CC
2439	BcaC05g30442	TM-CC
2440	BcaC06g30804	TM-CC
2441	BcaC06g30838	TM-CC
2442	BcaC06g30911	TM-CC
2443	BcaC06g30912	TM-CC
2444	BcaC06g30924	TM-CC
2445	BcaC06g30937	TM-CC
2446	BcaC06g31072	TM-CC
2447	BcaC06g31128	TM-CC
2448	BcaC06g31410	TM-CC
2449	BcaC06g31571	TM-CC
2450	BcaC06g31606	TM-CC
2451	BcaC06g31799	TM-CC
2452	BcaC06g31840	TM-CC
2453	BcaC06g32048	TM-CC
2454	BcaC06g32087	TM-CC
2455	BcaC06g32196	TM-CC
2456	BcaC06g32347	TM-CC
2457	BcaC06g32360	TM-CC
2458	BcaC06g32503	TM-CC
2459	BcaC06g32875	TM-CC
2460	BcaC06g32938	TM-CC
2461	BcaC06g33093	TM-CC
2462	BcaC06g33104	TM-CC
2463	BcaC06g33106	TM-CC
2464	BcaC06g33107	TM-CC
2465	BcaC06g33181	TM-CC
2466	BcaC06g33285	TM-CC
2467	BcaC06g33288	TM-CC
2468	BcaC06g33398	TM-CC
2469	BcaC06g33482	TM-CC
2470	BcaC06g33621	TM-CC
2471	BcaC06g33732	TM-CC
2472	BcaC06g33745	TM-CC
2473	BcaC06g33749	TM-CC
2474	BcaC06g33913	TM-CC
2475	BcaC06g34279	TM-CC
2476	BcaC06g34486	TM-CC
2477	BcaC06g34629	TM-CC
2478	BcaC06g34724	TM-CC

2479	BcaC06g34990	TM-CC
2480	BcaC06g35097	TM-CC
2481	BcaC06g35110	TM-CC
2482	BcaC06g35258	TM-CC
2483	BcaC06g36126	TM-CC
2484	BcaC06g36187	TM-CC
2485	BcaC06g36724	TM-CC
2486	BcaC06g36775	TM-CC
2487	BcaC07g36884	TM-CC
2488	BcaC07g36931	TM-CC
2489	BcaC07g36955	TM-CC
2490	BcaC07g37241	TM-CC
2491	BcaC07g37636	TM-CC
2492	BcaC07g37725	TM-CC
2493	BcaC07g37956	TM-CC
2494	BcaC07g38250	TM-CC
2495	BcaC07g39212	TM-CC
2496	BcaC07g39479	TM-CC
2497	BcaC07g39586	TM-CC
2498	BcaC07g39672	TM-CC
2499	BcaC07g40408	TM-CC
2500	BcaC07g40581	TM-CC
2501	BcaC07g40953	TM-CC
2502	BcaC07g41003	TM-CC
2503	BcaC07g41146	TM-CC
2504	BcaC07g41925	TM-CC
2505	BcaC07g41926	TM-CC
2506	BcaC07g42242	TM-CC
2507	BcaC07g42251	TM-CC
2508	BcaC07g42310	TM-CC
2509	BcaC07g42449	TM-CC
2510	BcaC08g43217	TM-CC
2511	BcaC08g43634	TM-CC
2512	BcaC08g44139	TM-CC
2513	BcaC08g44665	TM-CC
2514	BcaC08g45055	TM-CC
2515	BcaC08g45062	TM-CC
2516	BcaC08g45096	TM-CC
2517	BcaC08g45122	TM-CC
2518	BcaC08g45326	TM-CC
2519	BcaC08g45402	TM-CC
2520	BcaC08g45440	TM-CC
2521	BcaC08g45682	TM-CC
2522	BcaC08g45812	TM-CC
2523	BcaC08g46686	TM-CC
2524	BcaC08g46875	TM-CC
2525	BcaC08g46996	TM-CC
2526	BcaC08g47454	TM-CC
2527	BcaC09g49244	TM-CC
2528	BcaC09g49398	TM-CC
2529	BcaC09g49517	TM-CC
2530	BcaC09g49535	TM-CC
2531	BcaC09g49768	TM-CC
2532	BcaC09g49832	TM-CC
2533	BcaC09g50062	TM-CC
2534	BcaC09g50066	TM-CC
2535	BcaC09g50203	TM-CC
2536	BcaC09g50354	TM-CC
2537	BcaC09g50395	TM-CC
2538	BcaC09g50754	TM-CC
2539	BcaC09g50830	TM-CC
2540	BcaC09g50853	TM-CC

2541	BcaC09g51227	TM-CC
2542	BcaC09g51669	TM-CC
2543	BcaC09g51889	TM-CC
2544	BcaC09g52026	TM-CC
2545	BcaNung00166	TM-CC
2546	BcaNung00190	TM-CC
2547	BcaNung00399	TM-CC
2548	BcaNung01062	TM-CC
2549	BcaNung01287	TM-CC
2550	BcaNung01529	TM-CC
2551	BcaNung01838	TM-CC
2552	BcaNung02293	TM-CC
2553	BcaNung02306	TM-CC
2554	BcaNung02402	TM-CC
2555	BcaNung02849	TM-CC
2556	BcaNung02876	TM-CC
2557	BcaNung03202	TM-CC
2558	BcaNung03317	TM-CC
2559	BcaNung03401	TM-CC
2560	BcaNung03590	TM-CC
2561	BcaNung03950	TM-CC
2562	BcaNung04176	TM-CC
2563	BcaNung04291	TM-CC
2564	BcaNung04475	TM-CC
2565	BcaNung04965	TM-CC
2566	BcaNung05155	TM-CC
2567	BcaNung06438	TM-CC
2568	BcaNung06850	TM-CC
2569	BcaNung06890	TM-CC
2570	BcaNung07128	TM-CC

Reference:

Song X, Wei Y, Xiao D, *et al.* , 2021. *Brassica carinata* genome characterization clarifies U's triangle model of evolution and polyploidy in Brassica. *Plant physiology* **186**, 388-406.

Li P, Quan X, Jia G, Xiao J, Cloutier S, You FM, 2016. RGAugury: a pipeline for genome-wide prediction of resistance gene analogs (RGAs) in plants. BMC Genomics **17**, 852.
